# A unified view on enzyme catalysis by cryo-EM study of a DNA topoisomerase

**DOI:** 10.1038/s42004-024-01129-y

**Published:** 2024-02-28

**Authors:** Chiung-Wen Mary Chang, Shun-Chang Wang, Chun-Hsiung Wang, Allan H. Pang, Cheng-Han Yang, Yao-Kai Chang, Wen-Jin Wu, Ming-Daw Tsai

**Affiliations:** 1 Institute of Biological Chemistry, Academia Sinica, Taipei, 115 Taiwan; 2 Institute of Biochemical Sciences, National Taiwan University, Taipei, 106 Taiwan; 3 Present Address: Institute of Biochemistry and Molecular Biology, China Medical University, Taichung, Taiwan; 4 Present Address: Department of Biological Sciences, National University of Singapore, Singapore, Singapore

**Keywords:** Cryoelectron microscopy, Enzyme mechanisms, DNA-binding proteins, Viral proteins

## Abstract

The theories for substrate recognition in enzyme catalysis have evolved from lock-key to induced fit, then conformational selection, and conformational selection followed by induced fit. However, the prevalence and consensus of these theories require further examination. Here we use cryogenic electron microscopy and African swine fever virus type 2 topoisomerase (*Asfv*Top2) to demonstrate substrate binding theories in a joint and ordered manner: catalytic selection by the enzyme, conformational selection by the substrates, then induced fit. The *apo*-*Asfv*Top2 pre-exists in six conformers that comply with the two-gate mechanism directing DNA passage and release in the Top2 catalytic cycle. The structures of *Asfv*Top2-DNA-inhibitor complexes show that substantial induced-fit changes occur locally from the closed *apo*-conformer that however is too far-fetched for the open *apo*-conformer. Furthermore, the ATPase domain of *Asfv*Top2 in the MgAMP-PNP-bound crystal structures coexist in reduced and oxidized forms involving a disulfide bond, which can regulate the *Asfv*Top2 function.

## Abstract (web summary)

African swine fever virus type II DNA topoisomerase (*Asfv*Top2) is known to change DNA topology by employing a complex catalytic cycle and is involved in the early stages of viral gene replication, however, the structural and regulatory functions of *Asfv*Top2 remain underexplored. Here, the authors use cryo-EM to demonstrate six conformers of *apo*-*Asfv*Top2 as well as the structures of *Asfv*Top2-DNA-inhibitor complexes, revealing experimental evidence of their substrate binding mechanism that also shed light to other enzymes.

## Introduction

The past half century has seen major advancements in the theories for substrate recognition in enzyme catalysis from lock-key^1^ to induced fit^2^, then conformational selection^3–5^, and recently conformational selection followed by induced fit^4,6,7^. Furthermore, the *apo*-enzyme (ligand-free) was suggested to pre-exist as a subset of catalytically relevant conformers (termed catalytic selection here) for facilitating the conformational selection^7–9^. The concept of conformational selection started from the early report of multiple conformational states of myoglobin^10^ to the recent emphasis of conformational landscapes and conformational selection^4,6,7,11–16^. Of particular interest is the finding by Kern and coworkers that, even in the absence of ligands, adenylate kinase (Adk) in crystals pre-exists in three conformers with varying degrees of lid openness along the catalytic trajectory, suggesting that the structure of the *apo* enzyme has evolved to adopt conformations ready to bind its substrates and reaction intermediates^8^, a property termed catalytic selection herein. A recent study showed that this property is coupled with the conformational selection and induced fit mechanisms upon ligand binding, based on an advanced nuclear magnetic resonance (NMR) relaxation approach that enabled the structure determination of Adk at high-energy and minor populated states^7^. Related properties were also found in several other proteins^17^, though Adk has been most extensively investigated. In fact, the catalytic selection mechanism could be widespread, but only one structure is solved a time. For example, in the *apo* state of the four members of the X-family DNA polymerases, Pol β exists in an open form^18^ while Pol λ^19^, Pol μ^20^ and TdT^21^ pre-exist in the closed form.

**Fig. 1:**
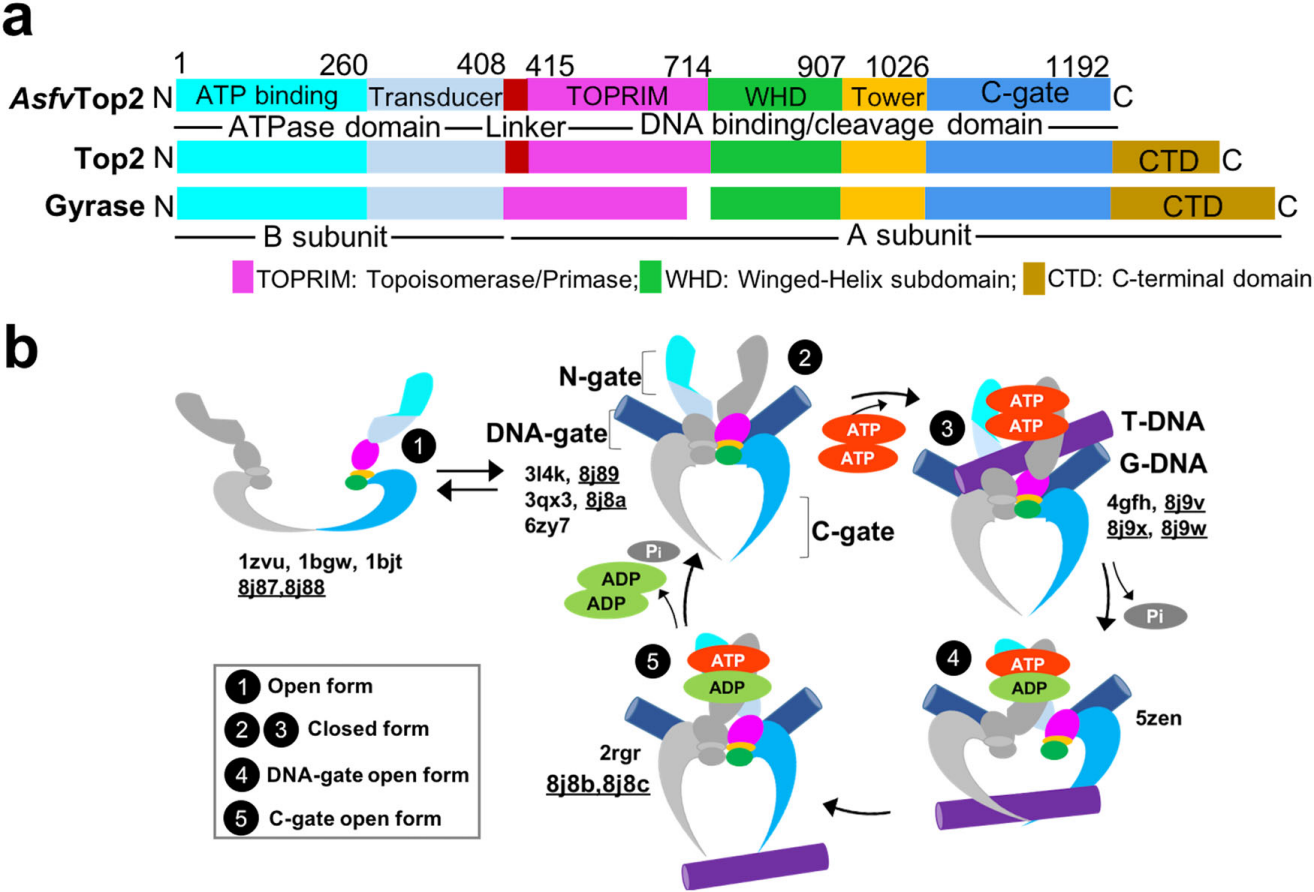
Top2 domain composition and the main conformational states in its catalytic cycle. **a** Schematic illustration for the subdomains of *Asfv*Top2, Top2 and gyrase. **b** Cartoon model illustration of the proposed Top2 conformational states (numbered in black circles)^40,41,44,47–51,58,59,64^ with corresponding subdomains colored as indicated in the scheme above. The cycle starts with the *apo*-Top2 existing in an open dimeric form (state 1), which closes upon Gate (G) DNA binding to the DNA cleavage core (state 2). Next, the N-terminal ATPase (N-gate) is closed in the presence of ATP to capture the Transport (T) DNA segment (state 3). Then a double-strand G-DNA break occurs transiently to enable the T-DNA passage through the open DNA-gate (state 4), with one ATP molecule concurrently hydrolyzed. Next, re-ligation of the cleaved G-DNA segment triggers opening of the C-gate, leading to the release of T-DNA (state 5). Finally, the enzyme returns to the state 2 by closing the C-gate and releasing ADP. These conformational states or their variants are often referred to as the open form (state 1) or closed form (states 2–3) based on the status of the DNA-gate, while states 4 and 5 are respectively described as DNA-gate open and C-gate open forms here. The PDB codes for the previously reported structures and those from this study (Table 1) are listed, with the latter underlined.

On the other hand, the relationship between protein dynamics and functions was often examined for relatively small monomeric proteins by NMR primarily, as in the signaling protein NtrC^22^, dihydrofolate reductase (DHFR)^11^ and tyrosine phosphatases^12^. The Adk study used X-ray crystallography, NMR, and MD simulation^7,8,23^, which well encapsulated the subtle yet significant local differences between the conformational sub-states. However, for these theories to be broadly applicable, they need to be demonstrated for large and multimeric enzymes that catalyze complicated reactions. The enzyme used in this study is a type II DNA topoisomerase (Top2) from African swine fever virus (Asfv), a dimeric multi-subdomain enzyme (271 kDa) that catalyzes multistep reactions to modulate DNA topology (Fig. 1).

It is now common to identify multiple structures from a single dataset in cryogenic-electron microscopy (cryo-EM)^24,25^. A vast majority of such conformers involve different ligand binding states or reaction intermediates. Only limited examples were found to address conformational variations in an *apo* enzyme^26–30^. In this work, we use cryo-EM to evaluate the current catalytic theories with *Asfv*Top2 and examine its induced fit conformational changes upon binding of ligands. The results allow us to propose a three-stage mechanism that encapsulates the substrate binding theories in a joint and order fashion. Furthermore, unique structural features and drug binding pocket of *Asfv*Top2 are unraveled for future antiviral innovation. The characterization of the disulfide bond formation in *Asfv*Top2 ATPase domain addresses a redox regulatory mechanism unique to topoisomerases.

## Results

### *Asfv*Top2 with complex gating mechanisms

Asfv is the only member of the *Asfarviridae* family, which belongs to the genetically and structurally complex eukaryotic large DNA viruses^31^. Albeit it poses a threat to worldwide agriculture with very high socioeconomic impact^32,33^, there is currently no effective vaccine or therapeutics. *Asfv*Top2 has been found to be involved in the early stage of viral gene replication^34–38^, and also detected at the intermediate and late stages of infection^34^. Although *Asfv*Top2 has low sequence identity to its eukaryotic and bacterial counterparts^38,39^, it harbors the general Top2 domain composition (Fig. 1a).

The domain composition of *Asfv*Top2 resembles that of the canonical Type IIA DNA topoisomerases (Top2), including the well-studied eukaryotic counterparts^40–42^, which form homodimers (Fig. 1a). Some other Top2 members, such as Topo IV and gyrase, are divided into two subunits and form heterotetramers^43,44^. Top2s change DNA topology by employing a complex catalytic cycle. Figure 1b outlines the major conformational states (1–5) of the ATP-fueled catalytic process, involving the proposed mechanisms based on a wealth of biochemical and structural studies for the truncated forms or subunits of Top2 from different species^40,41,45–52^. The catalytic process not only involves well-coordinated changes in protein quaternary structures to allow timely opening and closing of the N-, DNA-, and C-gates, but also ATP binding and sequential hydrolysis that relate the dimerization and catalytic activity of the ATPase domain to the mechanistic steps of the cycle^49,51,53–59^. However, structural and regulatory studies concerning how *Asfv*Top2 functions in the catalytic cycle and its drug binding modes have never been reported. Thus, it is an ideal system to test the recent theories of substrate recognition in catalysis, as well as to examine the catalytic mechanism in comparison with other Top2 enzymes. The structures we have obtained and their main properties are summarized in Table 1. Detailed structural data are listed in Tables 2–4. The detailed flow charts for solving the cryo-EM structures are shown in Supplementary Figs. 1–4.

**Table 1:** Summary of key structural information

Sample no.	Structure name	MgAMP-PNP	DNA	Inhibitor		Conf no.	DNAgate	C-gate	Resoln (Å)	PDB code	EMDB code
X-ray crystallography of *Asfv*Top2 ATPase domain											
1	ATPase-red	+	-	-		-	-	-	1.73	8JA2	-
2	ATPase-ox	+	-	-		-	-	-	1.14	8JA1	-
Cryo-EM structures of full-length *Asfv*Top2											
3	*Apo*-*Asfv*Top2	-	-	-		Ia	more open	closed	3.42	8J87	EMD-36062
						Ib	open	closed	3.49	8J88	EMD-36063
						IIa	closed	closed	2.31	8J89	EMD-36064
						IIb	closed	closed	2.51	8J8A	EMD-36065
						IIIa	closed	more open	2.43	8J8B	EMD-36066
						IIIb	closed	open	2.69	8J8C	EMD-36067
Cryo-EM reconstruction of cleavage core domain with DNA/drug bound											
4	EDI-1	+	Cut02a	etoposide	II		closed	closed	2.70	8J9V	EMD-36116
5	EDI-2	+	Cut02b	etoposide	II		closed	closed	2.74	8J9W	EMD-36117
6	EDI-3	+	Cut02a	*m*-AMSA		II	closed	closed	3.0	8J9X	EMD-36118
Cryo-EM reconstruction of entire *Asfv*Top2 with DNA/drug bound											
4	full complex	+	Cut02a	etoposide		II	closed	closed	3.68	-	EMD-36473

The formal PDB validation reports of all deposited structures and maps are provided in Supplementary Data 7–18.

**Table 2:** The statistics of cryo-EM data collection, structure refinement, and validation for the *apo-Asfv*Top2 structure in three states, containing six conformers

*apo*-*Asfv*Top2						
Data collection and processing						
Micrographs	8900					
Magnification	105,000					
Voltage (kV)	300					
Electron exposure (e^–^/Å^2^)	50					
Defocus range (μm)	−1.5 ~-2.5					
Pixel size (Å)	0.83					
Symmetry imposed	C2					
Initial particle images (no.)	3,260,785					
Conformer	Ia	Ib	IIa	IIb	IIIa	IIIb
PDB/EMDB code	8J87/EMD-36062	8J88/EMD-36063	8J89/EMD-36064	8J8A/EMD-36065	8J8B/EMD-36066	8J8C/EMD-36067
Final particle images (no.)	79,500	75,839	567,069	393,995	341,283	180,474
Map resolution (Å)	3.42	3.49	2.31	2.51	2.43	2.69
FSC threshold	0.143	0.143	0.143	0.143	0.143	0.143
Map resolution range (Å)	3.0–15.0	3.0–15.0	2.0–8.0	2.0–9.0	2.0–8.0	2.0–10.0
Refinement						
Initial model used (PDB code)	1ZVU^47^	Ia(8J87)	Robetta prediction^81^	IIa(8J89)	IIa(8J89)	IIIa(8J8B)
Model resolution (Å)	3.42	3.49	2.31	2.51	2.43	2.69
Model resolution range (Å)	3.0–15.0	3.0–15.0	2.0–8.0	2.0–9.0	2.0–8.0	2.0–10.0
Map sharpening *B* factor (Å^2^)	−96.5	−111.5	−80.8	−86.7	−79.7	−79.7
Model composition						
Protein residues	954	954	1502	1502	1518	1434
Non-hydrogen atoms	7846	7846	12,222	12,222	12,330	11,650
Ligand	0	0	0	0	0	0
*B* factors (Å^2^)						
Protein	157.69	194.85	96.39	110.18	109.82	104.39
R.m.s. deviations						
Bond lengths (Å)	0.002	0.002	0.002	0.003	0.002	0.002
Bond angles (°)	0.562	0.562	0.457	0.498	0.432	0.432
Validation						
MolProbity score	1.66	1.64	1.56	1.41	1.50	1.54
Clash score	7.20	5.61	5.77	7.45	5.43	4.89
Poor rotamers (%)	0.00	0.00	0.00	0.00	0.00	0.00
Ramachandran plot (%)						
Favored	96.11	95.16	96.39	97.99	96.49	95.91
Allowed	3.89	4.84	3.61	2.01	3.51	4.09
Disallowed	0.0	0.0	0.00	0.00	0.00	0.00

**Table 3:** The statistics of cryo-EM data collection, structure refinement, and validation for *Asfv*Top2/DNA/inhibitor structures (EDI-1, -2, -3) and the reconstruction of the full EDI-1 complex

	*Asfv*Top2:Cut02aDNA:etoposide (EDI-1)		*Asfv*Top2:Cut02bDNA:etoposide (EDI-2)	*Asfv*Top2:Cut02aDNA:*m-*AMSA (EDI-3)
Data collection and processing				
Micrographs	11786		10919	9846
Magnification	105,000		105,000	105,000
Voltage (kV)	300		300	300
Electron exposure (e^–^/Å^2^)	50		42	42
Defocus range (μm)	−1.5 ~−2.5		−1.5 ~-2.5	−1.5~−2.5
Pixel size (Å)	0.83		0.83	0.83
Reconstructions	Full complex	DNA binding/ cleavage domain	DNA binding/ cleavage domain	DNA binding/ cleavage domain
Symmetry imposed	C1	C2	C2	C2
Initial particle images (no.)	632,007	2,827,913	5,665,983	5,465,423
Final particle images (no.)	80,745	632,007	650,266	351,979
EMDB accession code	EMD-36473	EMD-36116	EMD-36117	EMD-36118
PDB accession code	_	8J9V	8J9W	8J9X
Map resolution (Å)	3.68	2.70	2.74	3.0
FSC threshold	0.143	0.143	0.143	0.413
Map resolution range (Å)	3.0–7.0	2.70–7.5	2.74–7.5	3.0–8.0
Refinement				
Initial model used (PDB code)	IIa(8J89)/6ZY7^69^		EDI-1(8J9V)	EDI-1(8J9V)
Model resolution (Å)	2.70		2.74	3.0
Model resolution range (Å)	2.70–7.5		2.74–7.5	3.0–8.0
Map sharpening *B* factor (Å^2^)	−116.5		−116.2	−144.9
Model composition				
Non-hydrogen atoms	13,918		13,859	13,890
Protein residues	1556		1550	1556
Ligands	Etoposide:2 nucleotide:60 Mg^2+^:2		Etoposide:2 nucleotide:60 Mg^2+^:2	*m*-AMSA:2 nucleotide:60 Mg^2+^:2
*B* factors (Å^2^)				
Protein	135.86		145.02	164.51
Nucleotide	159.88		178.38	213.38
Ligand	127.46		170.85	171.42
R.m.s. deviations				
Bond lengths (Å)	0.003		0.004	0.003
Bond angles (°)	0.551		0.797	0.743
Validation				
MolProbity score	1.80		1.89	1.77
Clashscore	8.0		8.0	10.0
Poor rotamers (%)	0.00		0.00	0.00
Ramachandran plot				
Favored (%)	95.49		93.86	94.59
Allowed (%)	4.51		6.14	5.41
Disallowed (%)	0.00		0.00	0.00

**Table 4:** The statistics of X-ray structure data collection, processing and structure refinement and validation of *Asfv*Top2 ATPase/MgAMP-PNP in reduced and oxidized states

	Reduced	Oxidized
PDB code	8JA2	8JA1
Data Collection		
Space group	P6_1_22	P6_1_22
Unit cell dimensions		
*a*, *b*, *c* (Å)	85.92, 85.92, 212.01	85.76, 85.76, 212.30
α, β, γ (°)	90, 90, 120	90.00, 90.00, 120.00
Resolution (Å)	43.21–1.73 (1.82–1.73)^a^	43.18–1.14 (1.20–1.14)^a^
*R*_merge_	0.069 (0.404)^a^	0.098 (1.210)^a^
*I*/σ*I*	18.6 (4.9)^a^	15.1 (2.1)^a^
Completeness (%)	99.0 (98.7)^a^	99.8 (98.6)^a^
Multiplicity	8.6 (8.4)^a^	20.1(13.2)^a^
Total reflections / Unique reflections	417449 (58802)^a^ / 48808 (6982)^a^	3356924 (312727)^a^ / 166668 (23634)^a^
CC_½_	0.999 (0.934)^a^	0.999 (0.696)^a^
Search model (PDB)	1PVG^55^	Reduced form (8JA2)
Structure refinement statistics		
*R*_work_/*R*_free_	0.1979 / 0.2163	0.1838 / 0.1870
No. of atoms		
Protein	3099	3078
AMP-PNP/Mg^2+^	31 / 1	31 / 1
Water	227	352
B-factors (Å^2^)^b^		
Protein	21.4	21.23
AMP-PNP/Mg^2+^	12.3 / 12.6	9.0 / 7.4
Water	25.5	21.5
r.m.s. deviations		
Bond lengths (Å)	0.05	0.007
Bond angles (°)	1.703	1.037
Ramachandran plot (%)^c^		
favored region	98.17	97.61
allowed region	1.83	2.39
disallowed region	0	0

^a^Numbers in parenthesis indicate the values in the highest resolution shell.

^b^Average B-factor calculated using the average B script in PyMOL Molecular Graphic System (Version 2.2.2).

^c^Ramachandran statistics generated from Phenix v.1.19^82^.

### Cryo-EM structures of *apo-Asfv*Top2

**Fig. 2:**
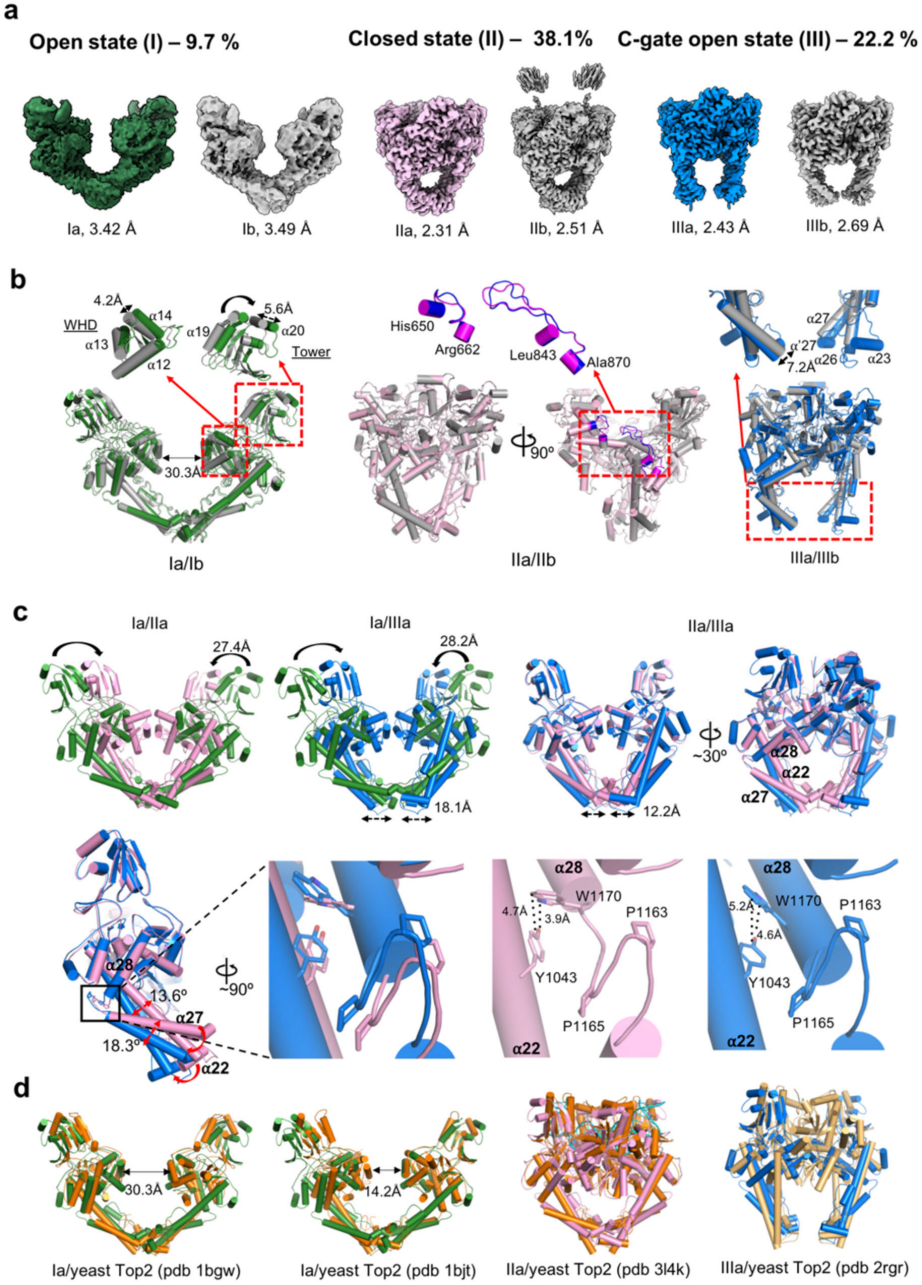
Structures of six conformers from *apo*-*Asfv*Top2. **a** Cryo-EM density maps for the six conformers of the full-length *Asfv*Top2. The selected particle percentages of conformer I, II and III are labeled. The densities for the subdomains were clearly defined at a resolution of 2.31–3.49 Å, except for the ATPase domain. Sparse densities for ATPase of conformer IIb can be seen, which indicates that these domains were not cleaved in the process of obtaining structures, but were flexible instead. The TOPRIM subdomain is not modeled in the conformer I. **b** High resolution cryo-EM reconstructions of the *apo*-*Asfv*Top2 cleavage core domain for the three states (I-III), with the two sub-conformers of each state superposed with each other. The distance between the Cα atoms of the Y744/Y’744 from the two α-helices 13 on the WHD subdomains of the two subunits is shown for Ia (Ia: 30.3 Å; Ib: 27.6 Å). The detailed differences between sub-conformers are shown in insets. Annotation of the helices is based on the structure-based alignment (Supplementary Figs. 5, 6). **c** Upper panel: Superposed structures showing major conformational changes between conformers Ia and IIa (left); Ia and IIIa (center), and IIa and IIIa (right). The TOPRIM subdomain is omitted for clarity. Lower panel: The hinge connecting helix α27 and α28 contains two non-conserved proline residues. The residues and contacts that contribute to the structural differences are labeled. **d** Superposition of Ia, Ib, IIa, and IIIa with the comparable crystal structures from the yeast Top2 cleavage core: pdb 1bgw (*apo*, TOPRIM omitted)^40^, 1bjt (*apo*, TOPRIM omitted)^46^, 3l4k (crossed-linked with dsDNA, closed state)^64^, and 2rgr (complexed with dsDNA, closed state with C-gate open)^48^, respectively.

We first performed the cryo-EM reconstruction of the full-length *apo*-*Asfv*Top2 without DNA or nucleotide. As shown in the flowchart of data processing (Supplementary Fig. 1), one single dataset produced three globally distinct conformational states I, II, and III, instead of two states reported recently^60^ (Fig. 2a and Table 2). These three conformational states resemble the open (**1**), closed (**2**-**3**), and C-gate open (**5**) conformational states, respectively, in Fig. 1b. The initial 3D volumes of *apo*-*Asfv*Top2 conformers II and III are contributed by more than half of the total selected particle population (Fig. 2a and Supplementary Fig. 1). Furthermore, these primary conformational states are each sub-classified into two conformers, a and b, exhibiting local differences. The superimposed pairs show subtle global conformational changes and dynamic motions (Supplementary Movie 1: 3D variability of *apo-Asfv*Top2). These six structures are resolved at resolutions of 2.31–3.49 Å, and their structural models are shown in superposed pairs in Fig. 2b with the variations between a and b conformers highlighted.

The distribution of the three primary co-existing *apo*-conformers is approximately 1:4:2 (Supplementary Fig. 1b). Even though such differences are modest energetically, they do suggest that the closed state II is the most stable state, followed by the C-gate open state III and then the open state I. These thermodynamic properties are reflected in their structural properties described in the following sections.

### Catalytic selection of *apo-Asfv*Top2

Global conformational changes between different conformational states are illustrated by overlaying the conformers Ia/IIa, Ia/IIIa, and IIa/IIIa (Fig. 2c). The changes in the Ia/IIa pair are mainly in the closing of the DNA-gate via the WHD subdomain. The major difference between IIa and IIIa is hinge-like movements of the coiled-coils (α22 and α27) with approximately 13.6° and 18.3° tilts, respectively, governing the mobile states of the C-gate (Fig. 2c). The key determinants of the movements likely involve the flexible turn (residues 1158-1167) connecting the helices α27 and α28. Of which, two proline residues (P1163/P1165) provide certain degree of flexibility^61^ to this joint region, which was previously defined as an elbow^46^. Additionally, the indole ring side chain of W1170 from *apo*-IIIa turns clockwise with the nitrogen atom of its pyrrole ring shifted away from the hydroxyl group of Y1043. The interaction between the two aromatic residues is weakened in the *apo*-IIIa, therefore the helix α22 is less engaged with the helix α28 and attains flexibility to introduce the swing motion at the coiled-coil region. The continuum of structural conversion from conformers Ia to IIIb is shown in Supplementary Movie 2 (Structure conversion of *apo-Asfv*Top2 conformers), which not only highlights the inherent dynamic nature of *apo*-Top2s, but also illustrates the unexpected yet functionally relevant conformational changes reminiscent of the process for the unidirectional strand passage in the Top2 catalytic cycle (Fig. 1b)^46,59,62,63^.

To further provide the structural basis and functional relevance, we compared the conformers from the *apo* form of *Asfv*Top2 with individual structures of the cleavage core domain of yeast Top2, where each structure was solved separately under different conditions. As shown in Fig. 2d, conformers Ia/Ib were aligned globally with two *apo*-yeast Top2 structures^45,46^. Subsequently, the TOPRIM subdomain is posited downwards for constituting the DNA binding groove, as shown by aligning conformer IIa and the DNA bound structure of yeast Top2 (pdb 3l4k^64^, closed form). Furthermore, conformer IIIa resembles the C-gate open structure of yeast Top2 (pdb 2rgr^48^), one of the key conformations for DNA strand transport^48,59^. The parallel conformational changes between Asfv IIa-to-IIIa in the absence of DNA and yeast 3l4k^64^-to-2rgr^48^ in the presence of DNA provide strong support for the catalytic selection mechanism of *Asfv*Top2.

### Conformational selection by ligands

In the conventional concept, the structural change between the *apo* form (yeast Top2 structure 1bgw^45^ or 1bjt^46^) and the DNA complex (2rgr^48^ or 3l4k^64^) can be attributed to induced fit, as has been addressed for 1bgw^45^ and 2rgr^48^ previously. However, it has not been addressed how DNA is able to bind a wide-open structure and induce large conformational changes, both globally and locally. Since the *apo*-state can pre-exist in multiple conformational states, the ligands are expected to select a conformer that is competent for ligand binding, according to the conformational selection theory^3–5^. Comparison with yeast Top2 structures suggested that the DNA complexes resemble *apo*-*Asfv*Top2 conformers II or III (*apo*-II or *apo*-III, respectively, Fig. 2d).

**Fig. 3:**
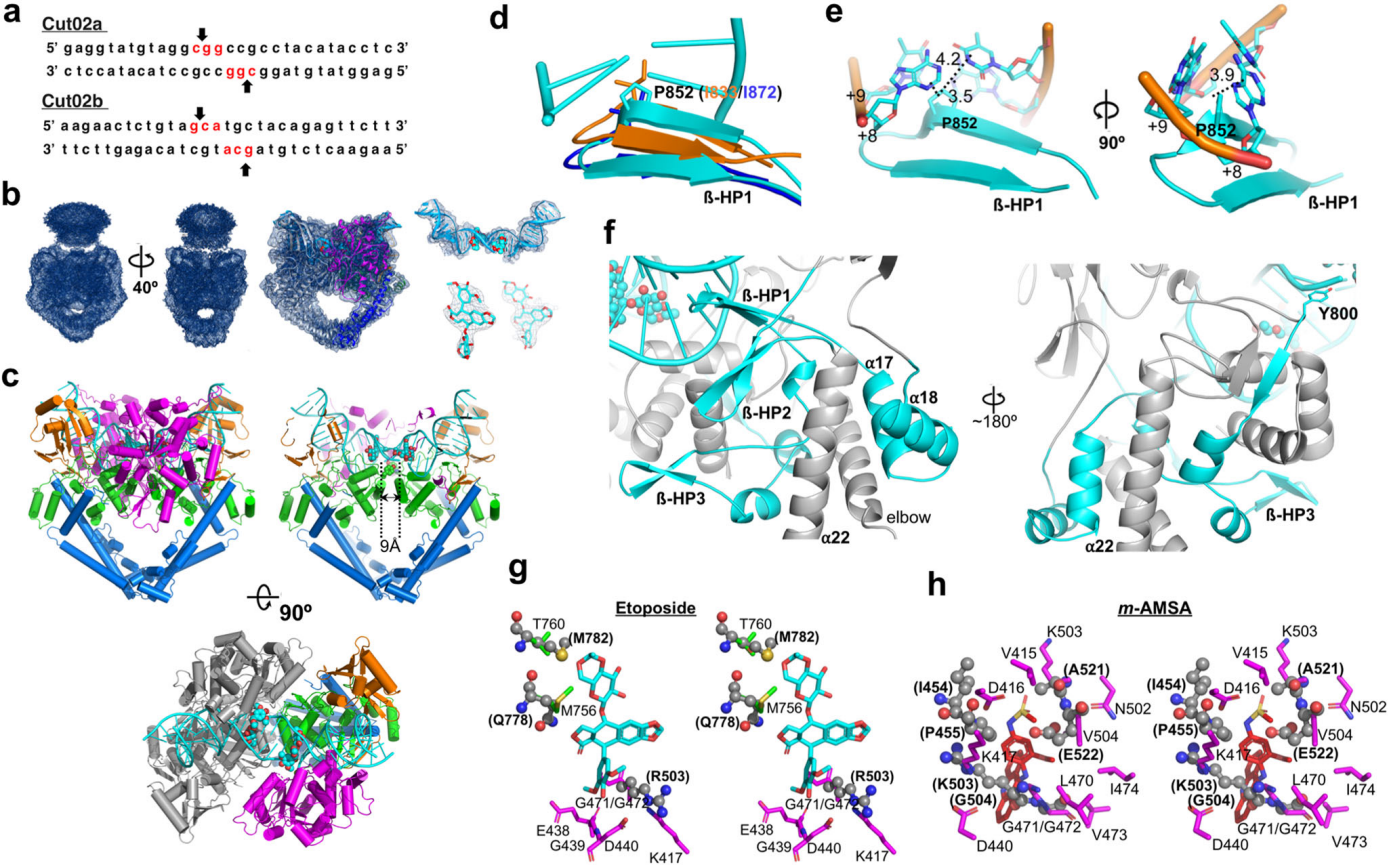
Cryo-EM structure of *Asfv*Top2-DNA-drug complex showing unique DNA interactions and drug binding modes. **a** The DNA sequences of the two G-DNA segments (Cut02a and Cut02b). The cleavage sites are indicated with arrows. **b** Left: The representative global cryo-EM density of the full-length complexes, *Asfv*Top2 Cut02a/inhibitor (EDI-1, resolution 2.70 Å, contoured at 0.034 σ). Center: The global map fitted with the modeled cartoon structure, contoured at 0.198 σ. Right: Maps of bound DNA and inhibitor molecules. **c** Left: The EDI-1 complex is shown in cartoon cylinders and colored based on the color scheme in Fig. 1a. Center/right: The structure is slabbed through to see the two α-helices 13 clearly with their distance labeled. The DNA and inhibitor are colored in cyan with cartoon and sphere presentations, respectively. **d** Enlarged view of the overlaid ß-HP1 region (colored in cyan, residues 846–861). The conserved isoleucine in yeast (orange)/human (blue) Top2s^64,66^ and *Asfv*Top2 P852 (shown in stick) emanate towards DNA to different extents. **e** The intercalation of P852 and its distances (Å) to adjacent DNA bases are labeled. **f** Left: the spatial location of the ß-HP1, adjacent to ß-HP2 (residues 822-834) and ß-HP3 (residues 1012-1025), and the elbow region. Right: ß-HP1 is linked to the catalytic Y800 through ß-HP3. The structural moieties mentioned are highlighted in cyan. **g**, **h** Structural superimposition between *Asfv*Top2 and human Top2 showing the differences in drug binding in stereo views. The corresponding human Top2 residues (pdb 3qx3, etoposide bound^66^, pdb 4g0u, *m*-AMSA bound^68^) are shown in spheres and labeled in bold letters in parentheses.

To obtain direct evidence for conformational selection, we performed structural analyses of *Asfv*Top2 complexes with DNA and an inhibitor. The sequences of DNA for the structural study are shown in Fig. 3a (Cut02a and Cut02b), which were chosen based on the DNA cleavage specificity of the active full-length *Asfv*Top2 (Supplementary Fig. 7) and described in Supplementary Note 1, Supplementary Fig. 8, and Supplementary Tables 1–3. Then we solved the cryo-EM structures of the full-length *Asfv*Top2 complexed with MgAMP-PNP (adenylyl-imidodiphosphate, a non-hydrolysable ATP analogue), DNA, and the Top2-targeting drug etoposide (a podophyllotoxin, with both Cut02a and Cut02b) (enzyme-DNA-inhibitor complexes EDI-1 and EDI-2, respectively) or *m*-AMSA (an aminoacridine, with Cut02a) (EDI-3) (2.70–3.0 Å, Tables 1 and 3) to dissect the specific binding of the DNA segments to the enzyme. Both etoposide and *m*-AMSA have been approved as anti-cancer drugs for human^65^, and have been shown to decrease the DNA decatenation activity of *Asfv*Top2 in vitro^35^.

The global structures of all three complexes resemble that of *apo*-II. As an example, the 3D maps of the complex with etoposide and Cut02a DNA (EDI-1) and those of the bound DNA and inhibitor are shown in Fig. 3b. The corresponding maps for EDI-2 and EDI-3 are shown in Supplementary Figs. 3 and 4, respectively. In all three structures, the DNA segment adopts a U-shaped conformation positioned on the DNA binding surface constituted of the TOPRIM, WHD, and Tower subdomains from both subunits (Fig. 3c). Importantly, despite the global similarity to *apo*-II by comparison Figs. 3c and Fig. 2b, there are substantial local changes as a result of induced fit. In the next two sections we first address the unique features of the *Asfv*Top2-DNA-inhibitor complexes relative to Top2s from other organisms, and then the induced fit conformational changes in the *Asfv*Top2.

### Uniqueness of *Asfv*Top2-DNA-drug complex

Specific enzyme-DNA interactions in the three complexes are shown by schematic diagrams (Supplementary Fig. 9). In comparison with the corresponding diagram for eukaryotic Top2^48,66^, the conserved DNA-intercalating residue (I872 in human or I833 in yeast Top2) is replaced by the P852 in *Asfv*Top2 (Supplementary Fig. 5b). The well-conserved isoleucine across eukaryotic and bacterial Top2s is involved in the DNA bending, and DNA-stimulated ATPase activity^48,67^. The alteration of the isoleucine to a proline by site-specific mutagenesis has been shown to dampen the *E. coli* Top2 (Top IV) functions in DNA bending and thus reduce ATPase activity^47^. Figure 3d shows that P852 and the conserved isoleucine from other Top2s residing at the ß-hairpin loop (annotated as ß-HP1 hereafter), protrude into DNA to different extents. The five-membered ring structure of P852 is intercalated between the DNA bases by an average distance of 4.0 Å (Fig. 3e). The ß-HP1 is buttressed by another two ß-HPs, and linked to the catalytic Y800 at the DNA-gate and helix α17 adjacent to the elbow (Fig. 3f). The potential functional relevance of this structural organization is elaborated in the following section. Additionally, *Asfv*Top2 lacks the essential conserved basic residue (K700 in yeast Top2) from the WHD subdomain (Supplementary Fig. 5b) for stabilizing the bent DNA conformation^48^. These distinct structural features likely differentiate the DNA modulation functions of *Asfv*Top2 from other Top2s.

Binding of the two Top2-targeting drugs etoposide and *m*-AMSA to *Asfv*Top2 is described in detail in Supplementary Note 2 and Supplementary Fig. 10. Structural comparison with human Top2-DNA-drug complexes^66,68^ highlights the distinctive residues from the two species facilitating the drug binding in different modes. For instances, *Asfv*Top2 lacks the conserved segment (PLR_503_GKXL) and polar residues that are characterized in its eukaryotic counterparts for the major interactions with the etoposide and *m*-AMSA, respectively (Fig. 3g, h). Based on the structural information, general guidelines for drug design targeting *Asfv*Top2 are summarized here: (1) a polycyclic aromatic core to assist drug intercalation into the DNA cleavage site as suggested for human Top2 targeting drug development^68^; (2) attaching a polar and bulky minor groove protruding moiety to the aromatic core that enhances the drug’s binding and specificity towards the minor groove-binding pocket of *Asfv*Top2.

### Induced fit from *apo*-IIa to EDI complex

**Fig. 4:**
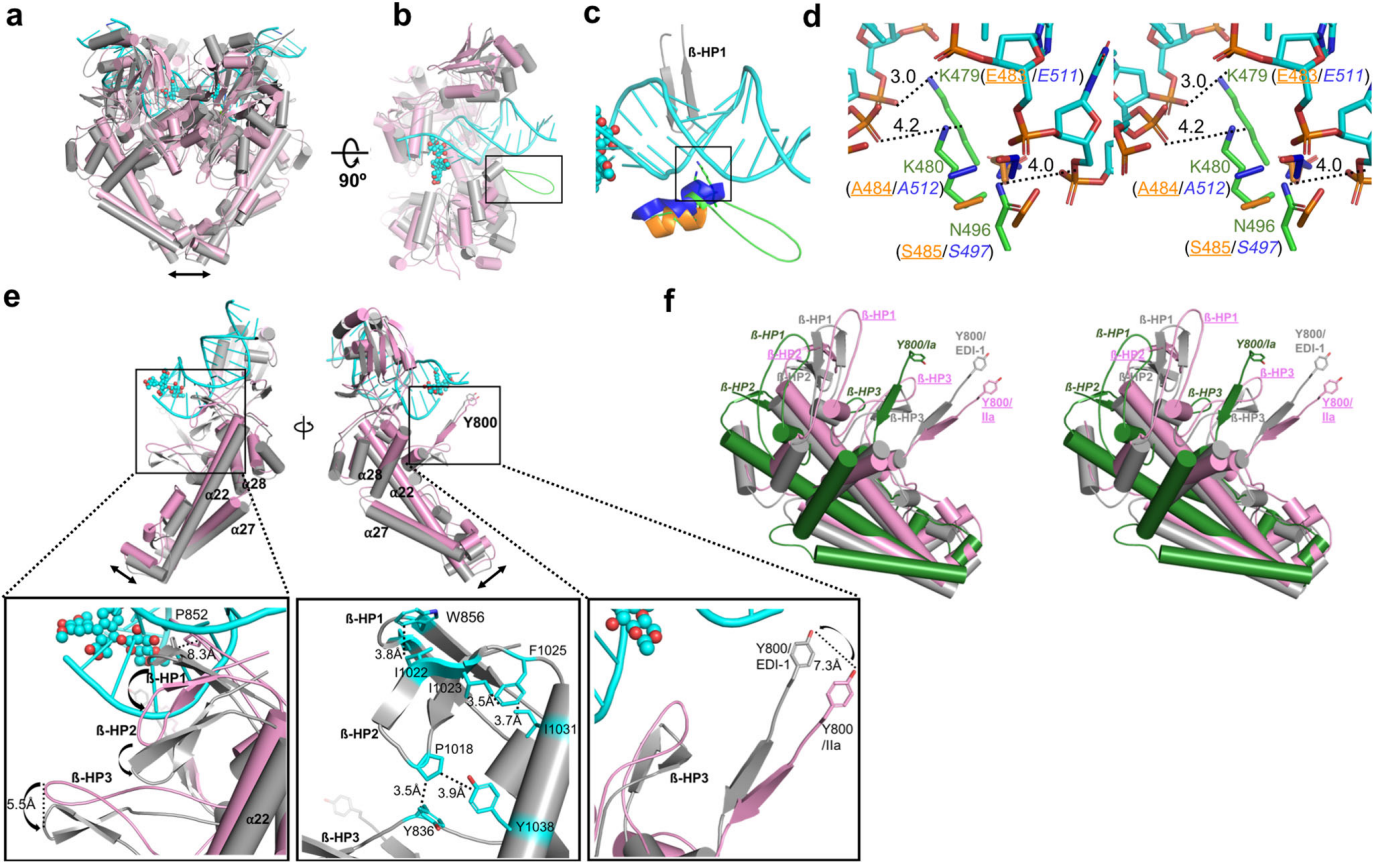
Global and local conformational changes induced by DNA binding. **a** The subdomain reorganization of *Asfv*Top2 is illustrated by aligning EDI-1 (gray) with *apo*-IIa (pink). **b** Half model of a, with a 90° rotation around the *x*-axis. The black rectangle highlights the extended loop that appears upon DNA binding. **c** The extended loop (residues 481-493, colored in green) shown inside the black square in panel b. The corresponding region in yeast/human Top2^64,66^ is colored in orange/blue, respectively. **d** The close-up stereo view of the specific interactions between *Asfv*Top2 and DNA in the square box in (**c**). The interactions are governed by the K479 and K480 of *Asfv*Top2, which are replaced with Glu and Ala (residue numbers in parentheses), respectively, in yeast/human Top2. **e** Upper panels: A global view of the local movements upon DNA binding with focus on the ß-HP1 region (left) and the catalytic Y800 (right). Lower panels: The enlarged view of the squared regions in the upper panel. Left: The three ß-HP loops from *apo*-IIa concurrently shift downward in the EDI-1 complex. Middle: The hydrophobic network that coordinates the concurrent movements of the three ß-HPs and α22 in the EDI-1 complex. Right: Shifting of the catalytic Y800 toward DNA in the complex. **f** Stereo view for the spatial locations of the three ß-HPs and Y800 in *apo*-Ia (colored in green with italic label), *apo*-IIa (colored in pink with underlined label), and the EDI-1 complex (gray).

By overlaying the *apo*-conformer IIa and the DNA/inhibitor bound *Asfv*Top2 structure, Fig. 4a shows that the protein conformations of the two structures are very similar except that binding of DNA/inhibitor causes small subdomain reorientations to constitute the protein-DNA interface and re-organize the dimer interfaces at DNA- and C-gates (Fig. 4a and Supplementary Movie 3: DNA-induced subdomain reorientation of *Asfv*Top2), which can be considered the induced fit by ligand binding to the pre-selected conformer IIa. Presumably, conformer IIb should work equally well due to its similarity to IIa.

Furthermore, comparison of the DNA-bound complex with the *apo*-*Asfv*Top2 conformers unravels a flexible extended loop (from residue 481–493) protruding from the TOPRIM subdomain that becomes visible upon DNA binding (Fig. 4b). Sequence alignment (Supplementary Fig. 5b) indicates that this long-extended loop is unique for *Asfv*Top2. As it resides at the vicinity of the DNA binding groove, and located opposite to the ß-HP1 across the DNA (Fig. 4c), it could be involved in DNA modulation, analogous to the potential role of the human Top2 CTD (missing in *Asfv*Top2) in facilitating strand passage process^69^. Figure 4d highlights the differences in the protein-DNA specific interactions in that region between the viral and eukaryotic Top2-DNA complexes. The key positively charged residues involved in the DNA binding are well conserved amongst Asfv orthologues (Supplementary Fig. 11), which likely illustrates the conserved functionality of the DNA responsive loop in *Asfv*Top2.

Another induced fit feature involves re-positioning of ß-HP1 and subsequent movements of the neighboring structural elements that likely harbor functional relevance. Aligning EDI-1 and conformer IIa (Fig. 4e left panels) indicates that ß-HP1 shifts downward in EDI-1, resulting in relocation of P852 by 8.3 Å to accommodate DNA binding. Simultaneously, several hydrophobic contacts are formed between ß-HP1 and ß-HP2, shifting the ß-HP2 downward to form hydrophobic interactions with ß-HP3 and helix α22 (Fig. 4e lower panels left and center), part of the coiled-coil C-gate region. The ß-HP3 residing beneath the ß-HP2 moves downward by 5.5 Å. Since the N-terminal end of ß-HP3 is linked to the catalytic residue Y800 through a long β-strand linker and two small α-helices (Figs. 3f and 4e lower right), the spatial location of the Y800 in EDI-1 is shifted from *apo*-IIa by a distance of 7.3 Å and moves upward toward DNA. These DNA-responsive conformational changes occurring in the DNA binding groove are relayed to the active site and potentially the C-gate region, which likely collectively coordinate the dynamic gating operation.

### Incompetence of *apo*-I for ligand binding

The results from the preceding section suggest that, even though the global conformation of *apo*-IIa is very similar to that of EDI-1, binding of DNA/inhibitor still induces small subdomain reorientations (Supplementary Movie 3: DNA-induced subdomain reorientation of *Asfv*Top2), yet coupled with substantial catalytically relevant conformational changes locally. On the other hand, as shown in Fig. 4f, the three ß-HPs and the catalytic Y800 in *apo*-Ia are farther away, relative to the corresponding moieties in *apo*-IIa, from the catalytically relevant positions in EDI-1, suggesting that it is less feasible for *apo*-I to bind the DNA substrate and undergo functionally relevant conformational changes. Taken together, our results suggest that it is advantageous for *apo*-II to coexist with *apo*-I through the catalytic selection process in order to be selected by the ligands, and to undergo further induced changes after ligand binding.

### Functional selection by redox regulation

**Fig. 5:**
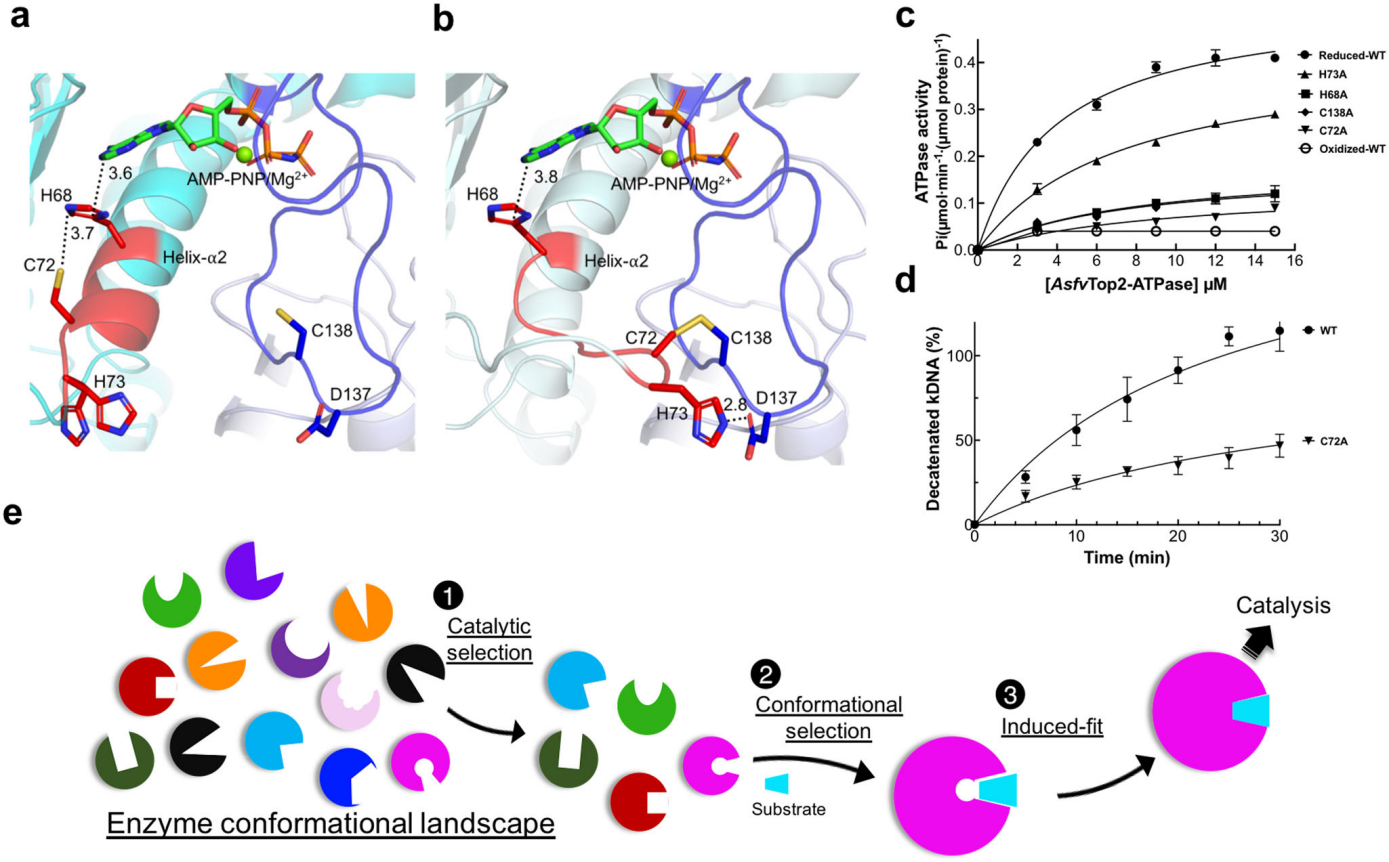
A potential redox regulatory mechanism unique to *Asfv*Top2. **a**, **b** The close-up view for the relative positions of MgAMP-PNP and the essential residues (C72, C138, H68, H73, and D137, all in stick presentation) involved in the potential regulatory mechanism. The reduced (**a**) and oxidized (**b**) forms are shown in cyan and pale cyan cartoon presentations, respectively. The disulfide bond between C72 and C138 is shown in brown stick. D137 and C138 are located at a long-extended surface loop (colored in blue) that harbors the active site residues interacting with the triphosphate group of the AMP-PNP. The segment E69-H73 that moves to form the disulfide bond is shown in red. **c** Analysis of ATP hydrolysis for the *Asfv*Top2 ATPase domain in the presence of 1 mM ATP and varying concentrations of the enzyme. The *y*-axis shows the specific activity from each measurement. Raw data are provided in Supplementary Data 5. **d** C72A mutation of the full-length *Asfv*Top2 resulted in significant reduction of DNA decatenation activity in comparison to the wild type over different time points. Detailed conditions are described in Supplementary Fig. 13c, d. Raw data are provided in Supplementary Data 6. Each data point in panels c, d includes the mean ± SE value from three independent reactions (*n* = 3) from the same batch of sample. Similar assay methods for ATPase and decatenation activities have been used previously for other Top2 proteins^35,83^. **e** A graphical summary illustrating that the enzyme-substrate recognition is mediated by the joint application of the substrate binding theories in order: (1) catalytic selection, (2) conformational selection, and (3) induced fit. The catalytic selection should be replaced by functional selection when referring to other functions instead of catalysis.

The ATPase domain regulates the Top2 catalytic cycle by binding ATP to induce closure of the N-gate, followed by hydrolyzing ATP to stimulate cleavage and passage of DNA^40,41,51,53,55,56,58,70^ (Fig. 1b), though the specific structural basis remains elusive. Here we report a different type of regulation by the ATPase domain of *Asfv*Top2. Using X-ray crystallography, we first found that the ATPase domain of *Asfv*Top2 seemed to coexist in two conformers in the MgAMP-PNP-bound crystal structure, and suspected that it could a mixture of reduced and oxidized forms. Then we resolved the crystal structure of *Asfv*Top2 ATPase domain complexed with MgAMP-PNP in the presence of the reducing agent β-mercaptoethanol (β-ME) (Fig. 5a, with density maps in Supplementary Fig. 12a). This structure showed unique features relative to the corresponding ATPase domain from *E. coli* and yeast Top2 (Supplementary Note 3 and Supplementary Fig. 13).

Next we address a new regulatory mechanism unique to *Asfv*Top2. The crystal structure of *Asfv*Top2 ATPase domain obtained from a non-reducing condition was solved at 1.14 Å resolution (Supplementary Note 3, with density maps in Supplementary Fig. 12). Structural analysis indicates that the structures from reducing and non-reducing conditions are without and with a C72-C138 disulfide bond, respectively (Fig. 5a, b). Interestingly, the disulfide bond formation involves a large movement of C72 to reach C138, along with relocation of H73 to pair with D137. Both residues and C138 are located at the solvent-exposed loops, in favor of the local motions. Additionally, this significant movement led to partial deformation of helix-α2 (Fig. 5b). The formation of disulfide bond between C72-C138 and the involvement of H73 are supported by mass spectrometry and NMR, respectively (Supplementary Fig. 14a, b).

Kinetic assays further indicated that the ATP hydrolysis activity was significantly dampened upon oxidation (Fig. 5c). Furthermore, under non-oxidizing condition, mutation of the residues involved in the disulfide bond formation, including H68A, C72A, H73A, and C138A, also led to different extent of inactivation, suggesting that these residues play additional roles in the non-oxidizing environment. H68 and C72 appeared to be crucial in holding adenosine moiety of AMP-PNP in position and thus both alanine mutations significantly attenuated the ATP hydrolysis activity. C138 resides at the long extended loop (residues F_125_-G_147_), encompassing the conserved ATP-lid that completely encloses the bound MgAMP-PNP at the active site (Fig. 5a, b, Supplementary Fig. 13b), therefore C138A mutation could affect the flexibility of the loop and subsequently the ATP hydrolysis. This effect appeared independent of the redox condition since the activity of C138A remained low in the oxidized form (Supplementary Fig. 14c). Thus, formation of the C72-C138 disulfide bond is most likely a naturally occurring way, in place of mutation, to enable the regulation of the ATPase activity and presumably the topoisomerase function. Importantly, this disulfide bond-mediated regulation of ATPase activity has not been shown in other Top2 enzymes. Furthermore, we showed that C72A significantly affected DNA decatenation activity of the full-length *Asfv*Top2 (Fig. 5d, Supplementary Fig. 14d, e), which demonstrates that the oxidation-mediated ATPase inactivation shown in Fig. 5c can regulate the primary function of the *Asfv*Top2.

These structural and functional analyses further support that the conformational states of the ATPase domain are coupled to the conformational and functional states of the cleavage core. Although the full-length structure of *Asfv*Top2 is not attainable due to conformational flexibility of the linker region that bridges two major functional domains, we have obtained a modeled structure based on the cryo-EM structure of the EDI-1 complex and the crystal structure of the ATPase domain, as described in Supplementary Note 4 and Supplementary Fig. 15.

Our result is in line with the redox-regulation of *Asfv*PolX^71,72^ and *Asfv*AP^73^, which have been shown to exist in both reduced and oxidized forms involving C81-C86 and C16-C20, respectively. The oxidized form of *Asfv*PolX has been shown to be specific to GG mismatch in addition to the four Watson-Crick base pairs^74^, whereas the reduced form has higher fidelity^75^. The structural basis of the low fidelity of the oxidized *Asfv*PolX has been elucidated by NMR^76^. *Asfv*PolX and *Asfv*AP, along with a low-fidelity Asfv DNA ligase^77^ are the key enzymes in the Asfv base excision repair system^78,79^. In line with the redox regulation of *Asfv*PolX and *Asfv*AP, formation of the disulfide bond by the cysteine pair (C72-C138) of *Asfv*Top2 can lower the activity of the topoisomerase, thus also save the ATP consumption and slow down DNA replication and cell growth, all of which may favor survival of the virus in under oxidative stress. Taken together, we propose that the redox mediated conformational transition of *Asfv*Top2 ATPase domain from reduced to oxidized state via the cysteine pair C72-C138 is a part of the functional selection, along with the redox regulations of *Asfv*PolX and *Asfv*AP, for the virus to adapt to the oxidizing environment of macrophage cytoplasm, analogous to catalytic selection in catalysis.

## Conclusion

As a conclusion, we quote a statement by D. E. Koshland from his 1994 review entitled “The Key-Lock Theory and the Induced Fit Theory”^80^ with relevant terms from recent literature inserted in parentheses: “A new theory (conformational selection^3–5^, or conformational selection followed by induced fit^4,6,7^) must explain all the existing facts that pertain to it at the time of its enunciation. Gradually the new theory becomes accepted and then acquires anomalies due to the new facts uncovered after its enunciation. That in turn generates a newer theory (catalytic selection^8,9^) which elicits new techniques (cryo-EM) to test it and its predictions. These new techniques then uncover facts which eventually require further new theories and so on. The new theories are built on components of the old principles.” In this study, the newest theory is the combination of the old components in order: (1) catalytic selection by the enzyme, (2) conformational selection by the substrates, and induced fit together, as illustrated in Fig. 5e. For signaling or other function, the first step should be replaced by “functional selection by the protein”. We propose that such a three-stage mechanism is universal in enzyme catalysis or protein-ligand recognitions, and expect that it will be demonstrated in more examples in the future.

## Methods

A brief summary is provided here. The functionally active full-length *Asfv*Top2 was over-expressed and purified from the yeast protein expression system (Supplementary Fig. 7) for cryo-EM structure determination of *apo*-*Asfv*Top2 and its DNA-inhibitor bound complexes (Supplementary Figs. 1–4). The functional studies performed included DNA decatenation, DNA relaxation, and cleavage assays (Fig. 5d, Supplementary Figs. 7, 8, 14). The active *Asfv*Top2 ATPase domain and its mutants were over-expressed and purified from *E. coli* for crystal structure determination (Supplementary Figs. 12, 13), redox studies, ATP hydrolysis activity assays (Figs. 5), 1D NMR, and mass spectrometry (Supplementary Fig. 14). The raw data for the functional and biophysical analyses are provided in Supplementary Data 1–6. A detailed explanation of all the procedures can be found in the Supplementary Methods section.

### Reporting summary

Further information on research design is available in the Nature Portfolio Reporting Summary linked to this article.

### Supplementary information

Peer Review File

Supplementary information

Description of Additional Supplementary Files

Supplementary Movie 1

Supplementary Movie 2

Supplementary Movie 3

Supplementary Data 2

Supplementary Data 5

Supplementary Data 7

Supplementary Data 8

Supplementary Data 9

Supplementary Data 10

Supplementary Data 11

Supplementary Data 12

Supplementary Data 13

Supplementary Data 14

Supplementary Data 15

Supplementary Data 16

Supplementary Data 17

Supplementary Data 18

Reporting Summary

## Data Availability

The coordinates and structure factors for *Asfv*Top2 ATPase domain in the reduced and oxidized states have been deposited in the Worldwide Protein Data Bank (wwPDB) under the accession codes: 8JA2 and 8JA1. Cryo-EM maps and the corresponding coordinate files for the *apo* full length *Asfv*Top2 have been deposited in the wwPDB under accession codes: 8J87, EMD-36062 (conformer Ia); 8J88, EMD-36063 (conformer Ib); 8J89, EMD-36064 (conformer IIa); 8J8A, EMD-36065 (conformer IIb); 8J8B, EMD-36066 (conformer IIIa); and 8J8C, EMD-36067 (conformer IIIb). Accession codes for the complexes are: 8J9V, EMD-36116 (Cut02aDNA/etoposide-bound, EDI-1); 8J9W, EMD-36117 (Cut02bDNA/etoposide-bound, EDI-2); and 8J9X, EMD-36118 (Cut02aDNA/*m*-AMSA-bound, EDI-3). The 3.68 Å cryo-EM map of full-length DNA/etoposide/AMP-PNP-bound *Asfv*Top2 can be accessed in the EMD-36473. The raw data for the functional and biophysical analyses are provided in Supplementary Data 1-6. The formal PDB validation reports of all deposited structures and maps are provided in Supplementary Data 7–18. The files of Supplementary Data 1, 3, 4, 6 have been stored in Figshare with 10.6084/m9.figshare.25123829.
