# Supplementary material for: A unified view on enzyme catalysis by cryo-EM study of a DNA topoisomerase
Source: Commun Chem. 2024 Feb 28;7:45. doi: 10.1038/s42004-024-01129-y (PMC10901890; doi:10.1038/s42004-024-01129-y)
Supplement: Supplementary file 2 — Supplementary information [file 42004_2024_1129_MOESM2_ESM.pdf]

Supplementary Information

A unified view on enzyme catalysis by cryo-EM study of a DNA topoisomerase

Chiung-Wen Mary Chang[1,2], Shun-Chang Wang[1], Chun-Hsiung Wang[1], Allan H. Pang[1,3], Cheng-Han Yang[1], Yao-Kai Chang[1], Wen-Jin Wu[1], Ming-Daw Tsai[1,4*]

[1]Institute of Biological Chemistry, Academia Sinica, Taipei 115, Taiwan.

[2]Present address: Institute of Biochemistry and Molecular Biology, China Medical University, Taichung, Taiwan.

[3]Present address: Department of Biological Sciences, National University of Singapore, Singapore.

[4]Institute of Biochemical Sciences, National Taiwan University, Taipei 106, Taiwan.

[*]Corresponding author. Email: mdtsai@gate.sinica.edu.tw

This PDF file contains supplementary information in the following order:

Supplementary Methods

Supplementary Notes 1-4

Supplementary Figures 1-15

Supplementary Tables 1-3

Supplementary References

Additional files uploaded separately:

Supplementary Movies 1-3

Supplementary Data 2, 5, 7-18. (Supplementary Data 1, 3, 4, 6 have been stored in Figshare with DOI: 10.6084/m9.figshare.25123829.)

Reporting Summary

Editorial Policy Checklist

Supplementary Methods

DNA constructs. The original pYES2-*Asfv*Top2 was kindly provided by Dr. Alexandre Leitão[1]. This construct was further modified to remove the extra 66 nucleotides from the 3'-end of *Asfv*Top2 coding gene and fully sequenced (Genomics, Taipei, Taiwan). The modified construct was used as a template for obtaining the full-length wild-type *Asfv*Top2 and C72A full-length protein mutant in *S. cerevisiae* cells (EGY48 strain). Using this construct as a template, the gene corresponding to the N-terminal ATPase domain of *Asfv*Top2 (residues 1-408) was amplified by PCR and subcloned into bacterial expression plasmid pET29c using restriction sites *Nde*I and *Xho*I. This resulted in the expression construct of *Asfv*Top2 ATPase with a N-terminal His_6-tag cleavable by TEV protease. To generate H68A, C72A, H73A, C138A mutants of the domain, the following mutagenic primers (listed below) synthesized from Genomics (Taipei, Taiwan) were used. Successful mutagenesis was confirmed by DNA sequencing (Genomics, Taipei, Taiwan). The following pairs of primers were used:

WT:	f: 5'ctagctagcatggaagcgtttgaaatcagcgattc3'	r: 5'gtcgctcgagttaatgaaatttccactgagtatttc3'
H68A:	f: 5'catcgtaaatgccacggatgctgaaagagcttgccatagc3'	r: 5'gctatggcaagctctttcagcatccgtggcatttacgatg3'
C72A:	f: 5'ggatcatgaaagagctgcccatagcaaaacaaaaaagg3'	r: 5'ccttttttgttttgctatgggcagctctttcatgatcc3'
H73A:	f: 5'ggatcatgaaagagcttgcgctagcaaaacaaaaaaggtaacc3'	r: 5'ggttaccttttttgttttgctagcgcaagctctttcatgatcc3'
C138A:	f: 5'caataaggccaaggacgctatcaagggggggaacc3'	r: 5'ggttcccccccttgatagcgtccttggccttattg3'

Expression and purification of full-length *Asfv*Top2. The protein expression plasmid pYES2-*Asfv*Top2 with C-terminal 6x His-tag was transformed into *S. cerevisiae* cells (EGY48 strain), incubated on a SD-U selection plate (yeast nitrogen base without amino acids, synthetic dropout mix without uracil, 2 % w/v agar, 2 % glucose) at 30 °C for 2-3 days. The competent cell preparation and transformation protocols were as previously described[2]. First, a pre-culture broth was prepared and used as a short-term stock. About 5 colonies (2-3 mm in diameter) on the SD-U selection plate were inoculated into 5 ml SD-U selection broth (with the same recipes of the SD-U selection plate without agarose) at 30 °C, 220 rpm for 30 h to reach a stationary phase with $O.D._{600}$ ~ 5. This pre-culture was used to prepare a starter culture for the following large-scale protein expression. A starter culture was prepared by adding 24 μl of the pre-culture broth ($O.D._{600}$ =5) into a new 5 ml SD-U selection broth and incubated at 30 °C with a shaking speed of 220 rpm for 24 h to reach the mid-log phase ($O.D._{600}$ =1.8-2). Then, a large scale culture was constructed

by adding a 5 ml starter culture into a 500 ml YPAGyL rich broth (5 g yeast extract, 10 g peptone, 50 mg adenine hemisulfate, 1.5 % w/v glycerol, 2 % w/v lactic acid, and neutralized with 3.125 g NaOH) in a 2 L baffled bottom bottle. The culture was incubated at 120 rpm shaking speed, 30 °C (Innova 44/44R incubator) for about 24 h to reach mid-log phase ($O.D._{600}$ =1.8-2), at which point protein expression was induced by adding 20 % w/v galactose to a final concentration of 2 % w/v for 6-8 h.

Cells were harvested and lysed in a buffer containing 50 mM Tris-HCl, pH 7.5, 0.5 M NaCl, 0.2% Triton X-100, 5 mM ß-mercaptoethanol (ß-ME), 20 mM imidazole and 0.1 mM phenylmethylsulfonyl fluoride (PMSF) using Avestin EmulsiFlex-C3 homogenizer. After a high-speed centrifugation, the supernatant was incubated with pre-equilibrated Ni^{2+}-charged Chelating Sepharose™ Fast Flow medium (GE Healthcare) for 40 mins at 4 °C. The beads were then sequentially washed with wash buffer 1 (20 mM Tris-HCl, pH 7.5, 0.5 M NaCl, 0.2 % Triton X-100, 5 mM ß-ME, 50 mM imidazole), wash buffer 1 with NaCl increased to 1 M, and wash buffer 2 (20 mM Tris-HCl, pH 7.5, 0.3 M NaCl, 10 % glycerol, 50 mM L-arginine, 50 mM L-glutamic acid, 5 mM ß-ME, 50 mM imidazole). The full-length *Asfv*Top2 was eluted with the wash buffer 2 but with higher imidazole concentration (200 mM). The purified protein was concentrated and injected into Superdex 200 increase 10/300 GL gel filtration column using the buffer consisting of 20 mM Tris-HCl, pH 7.5, 0.1 M NaCl, 50 mM L-arginine, 50 mM L-glutamic acid, 2 mM ß-ME, 5 mM $MgCl_2$. The protein for *apo Asfv*Top2 cryo-EM sample was purified without $MgCl_2$. The C72A mutant was produced and purified using the same procedure.

Expression and purification of ATPase domain. The pET29c-ATPase was transformed into *E. coli* BL21-CodonPlus(DE3)-RIPL and grown in Luria broth containing 50 µg/ml kanamycin and 25 µg/ml chloramphenicol at 37 °C to $O.D._{600}$ ~ 0.6. Protein production was induced by addition of isopropyl-β-D-thiogalactopyranoside (IPTG) to a final concentration of 0.1 mM at 16 °C for 16 h. 2 L cell culture were harvested by centrifugation and resuspended in the lysis buffer (20 mM Tris-HCl pH 8, 0.3 M NaCl, 20 mM ß-ME, 5 mM imidazole), followed by lysis using Avestin EF-C3 homogenizer. After a high-speed centrifugation, the clarified lysate was isolated and incubated with 3 ml cOmplete His-Tag Purification Resin (Roche) at 4 °C for 1 h gentle stirring. Resins containing the bound protein was loaded and passed through a gravity flow open column, followed by wash with 200 mL buffer containing 20 mM Tris-HCl pH 8, 0.3 M NaCl, 5 mM ß-ME, 7 mM

imidazole. A second wash was carried out by passing through the same wash buffer, but with higher NaCl concentration (0.8 M). The resins were then washed with stabilization buffer (20 mM Tris-HCl pH 8, 0.2 M NaCl, 5 mM ß-ME, 7 mM imidazole, 5 % glycerol) before the protein eluted out with buffer containing 20 mM Tris-HCl pH 8, 0.2 M NaCl, 5 mM ß-ME, 200 mM imidazole, 5 % glycerol. The N-terminal His-tag was removed by 1 h incubation with TEV protease at 22 °C, before loading into Superdex-75 (10/60) gel filtration column (GE Healthcare), with buffer consisting of 20 mM Tris pH 8, 0.15 M NaCl, 5 mM $MgCl_2$, 5 % glycerol, 2 mM ß-ME. The protocol above was optimized to obtain ATPase domain in reduced state. All mutants were purified using the same procedure. For oxidized samples, the same procedure was carried out but without the presence of ß-ME in the gel filtration buffer.

Protein crystallization and structure determination of *Asfv*Top2 ATPase. Peak fractions containing the purified *Asfv*Top2 ATPase proteins from the gel filtration chromatography were pooled together and concentrated. The purified *Asfv*Top2 ATPase with/without ß-ME were concentrated to 15 mg/ml. *Asfv*Top2 ATPase proteins in reduced state were used as is for the crystallization trials while protein samples for the oxidized state were kept at the 4 °C and gently stirred for few seconds daily for 2 weeks. Protein samples in both states were mixed with 1 mM AMP-PNP (Roche) and kept for at least 1 h before crystallization experiments. The crystallization conditions were screened using pre-formulated commercial screening kits: Wizard, Magic, PACT, JCSG(+), Ligand-Friendly and LiCl screens (Hampton Research, Molecular Dimensions). Screening was carried out using 96-well sitting-drop vapor diffusion method while optimization of crystallization hits was done by hanging drop method in 24-well trays. Crystals grew best under this condition: 0.1 M Tris-HCl pH 8.5, 20-25 % PEG 3350, 0.2-0.4 M $LiSO_4$ at 18 °C. Cryoprotection was carried out by transferring crystals to their mother liquor supplemented by 25 % glycerol before getting picked using a litholoop (Molecular Dimensions Ltd.) and quickly plunging into liquid nitrogen. The X-ray diffraction data were collected at the National Synchrotron Radiation Research Center beamline 05A (Hsinchu, Taiwan), processed and scaled using *XDS*[3] and SCALA[4], respectively. Molecular replacement was carried out using BALBES in the CCP4 online server[5] with the yeast Top2 ATPase domain (PDB: 1PVG) as the initial search model[6] (**Table 4**). Both reduced and oxidized forms of the *Asfv*Top2 ATPase domain complexed with MgAMP-PNP were resolved in the $P6_122$ space group with one molecule per asymmetric

unit at 1.73 and 1.14 Å resolution, respectively. A biologically relevant symmetry mate is present to form a superimposable fit to the homodimer of yeast Top2 ATPase protein[6], in agreement with the PISA assembly analysis result[7]. Refinement was carried out using Phenix v.1.19[8], and iterative cycles of manual building in Coot v.0.8.8[9]. The final model was validated by MolProbity[10]. The detailed statistics regarding data process, model refinement and validation were listed in **Table 4**. A summarized analysis of the structures of the *Asfv*Top2 in reduced and oxidized states is presented in **Supplementary Note 3**.

DNA relaxation assay. An individual reaction contained 2.1 pmol *Asfv*Top2 was incubated at 37 °C with 0.6 pmole of supercoiled pUC19 plasmid in a 20 µl reaction mixture. The reaction buffer contained 10 mM Tris-HCl pH 7.5, 50 mM NaCl, 6 mM $MgCl_2$, 2 mM DTT, 2 mM ATP. After 60 mins, reactions were stopped by adding 1.0 % SDS. Agarose gel electrophoresis was used to monitor the conversion of supercoiled pUC19 to the relaxed form. Samples were run on a 1.0 % agarose, 1X Tris-acetate EDTA buffer (TAE) gel, at 100 V/cm for 30 mins at room temperature. The raw images of electrophoresis results were taken by Syngene U:Genius Gel Documentation System. The intensities of DNA bands were quantified by ImageJ ver.1.53v[11]. Data for the relaxation assays are presented in **Supplementary Fig. 7b**.

DNA cleavage assay. A 398 bp fragment that amplified by PCR from position 1047 to 1444 of pUC19 was used to analyze the cleavage sites of *Asfv*Top2. The reaction contained 4-6 pmol recombinant *Asfv*Top2 protein and 0.4 pmol DNA fragments in a total of 20 µl buffer (10 mM Tris-HCl pH 7.5, 50 mM NaCl, 6 mM $CaCl_2$, 2 mM ATP). Calcium ions were used to enhance the cleavage reactions[12]. Reaction was incubated for 6 mins at 37 °C, and then stop by adding 1 µl 10 % SDS. Finally, the enzyme was removed by incubating the reaction mixture with 2 µg proteinase K at 45 °C for 30 min. A summarized result of this assay can be found in the **Supplementary Note 1**.

Mass spectrometry analysis. Both reduced and oxidized samples were buffer exchanged with 50 % methanol/0.5 % formic acid using Amicon ultra 0.5 ml centrifugal concentrators. An aliquot corresponding to one pmol of the protein sample was injected to LockSpray Exact Mass Ionization Source (Waters, Milford, MA) with a syringe pump (Harvard Apparatus, MA) and held a flow rate

of 3 µl/min throughout the analysis. The mass of the intact protein was determined using SYNAPT G2 HDMS mass spectrometer (Waters, Milford, MA). The mass spectra were acquired in the 400–2050 range of mass-to-charge (m/z); and these were deconvoluted to zero-charge spectrum using MaxEnt1 algorithm of the MassLynx 4.1 software (Waters, Milford, MA). MS data are presented in **Supplementary Fig. 14a**.

Nuclear magnetic resonance (NMR) spectroscopy. NMR data were collected at 298 K on a Bruker Avance 800 MHz NMR spectrometer equipped with a cryogenic probe. Samples for the ATPase domain in reduced, oxidized and the H73A mutant in reduced states were analyzed by one dimensional NMR. The NMR sample concentrations were 72 µM, 150 µM and 300 µM for the WT oxidized form, WT reduced form, and the reduced H73A mutant, respectively. The reduced NMR samples contained 50 mM sodium borate, 150 mM NaCl, 8.9 µM sodium trimethylsilyl propanesulfonate (DSS), 10 % D_2O and 5 mM DTT-d_{11} at pH 8.0, where the oxidized sample did not contain the reducing agent DTT. Data collection: water suppression was achieved using the W5 WATERGATE scheme[13], 13.9466 ppm (11160.714 Hz) spectral width, 16384 data points, 1.5 second interscan delay, number of scans were 4096, 1024 and 256 scans for the WT oxidized form, WT reduced form, and the reduced H73A mutant, respectively. Data processing was done under Topspin 3.6, zero filling was used to increase the digital resolution, and a line broadening factor (LB) of 3 was used in the exponential multiplication of the FID. Chemical shifts were referenced to the internal chemical shift standard of DSS (set to 0 ppm). NMR results are presented in **Supplementary Fig. 14b**.

ATPase activity assay. The *Asfv*Top2 ATPase activity was examined using the established Malachite Green assay[14] to measure the free phosphate (P_i) group released from the ATP hydrolysis. Briefly, 200 µl reaction containing 20 mM Tris-HCl pH 8, 50 mM NaCl, 1.0 % glycerol, 1 mM ATP (Sigma-Aldrich) and 5 mM $MgCl_2$ with *Asfv*Top2 ATPase (3 to 15 µM) was performed in triplicate experiments. After 15 mins incubation at 37 °C, the reactions were stopped by adding the 800 µl Malachite Green color reagent. Then the mixture was added with 100 µl 34 % citrate solution before obtaining the absorbance $O.D._{650}$ reading by DU® 800 spectrophotometer (Beckman Coulter). The amount of the released P_i was calculated from a standard curve obtained

by measuring the $O.D._{650}$ reading under a series of known K_2HPO_4 concentration. Standard curve was made for each individual assay. Data for the activity assays are presented in **Fig. 5c**.

DNA decatenation assay. Reactions were carried out under 37 °C for up to 30 mins in a total of 20 µl reaction mixture, containing 100 ng of kinetoplast DNA (*k*DNA) from *Crithidia fasciculata* (TopoGEN, Inc). The reaction buffer contained 10 mM Tris-HCl pH 7.5, 80 mM NaCl, 6 mM $MgCl_2$, 2 mM DTT, 0.1 mg/ml BSA, 2 mM ATP (Sigma-Aldrich). The final concentration range of *Asfv*Top2 was 0.5-10 nM. Reactions were stopped by adding EDTA (pH 8.0) to the final concentration, 25 mM. Then protein digestion was carried out by adding 2 µg proteinase K at 45 °C for 30 mins. The final reaction mixtures were electrophoresed in a 1.0 % (w/v) agarose gel, with 1X TAE buffer at 100 V/cm for 30 mins at room temperature. The raw images of electrophoresis results were taken by Syngene U:Genius Gel Documentation System. The intensities of DNA bands were quantified by ImageJ ver.1.53v[11]. Data for the assays are presented in **Fig. 5d** and **Supplementary Fig. 14c**, **d**.

Nucleic acid preparation for cryo-EM studies. Using the information from the DNA cleavage assay (**Supplementary Note 1**), two asymmetric synthetic oligonucleotides (17 bp and 13 bp) were prepared to mimic a doubly nicked 30 bp DNA duplex. The following ssDNA was synthesized and obtained from Genomics (Taipei, Taiwan); for the 17 bp: Cut02a: 5'-ggccgcctacatacctc-3'; Cut02b: 5'-catgctacagagttctt-3', and 13 bp: Cut02a: 5'-gaggtatgtaggc-3'; Cut02b: 5'-aagaactctgtag-3'. The 0.1 mM oligonucleotides were dissolved in the DNAse-free water. The dsDNA was annealed by mixing the asymmetry paired 17 bp and 13 bp oligonucleotides in 1:1 molar ratio, incubated at 95 °C for 5 mins, followed by cooling down to 20 °C, with a descending temperature increments of 5 °C every 3 mins.

Nucleoprotein complex assembly for cryo-EM. Freshly purified *Asfv*Top2 (0.3 mg/ml, equivalent to ~1 µM of dimer in a buffer containing 20 mM Tris pH 7.5, 0.1 M NaCl, 50 mM L-Arg/L-Glu, 5 mM $MgCl_2$ and 2 mM ß-ME) was mixed with the doubly nicked 30 bp DNA duplex at 1:2 molar ratio at room temperature (20 °C) for 30 mins. Etoposide or *m*-AMSA (Sigma-Aldrich) was added at a final concentration of 0.5 mM and incubated for 30 mins at room temperature. Subsequently, AMP-PNP (final concentration of 5 mM, Roche) was added to *Asfv*Top2

DNA/inhibitor complex, and then incubated for 30 mins at room temperature. Finally, CHAPSO (Sigma-Aldrich) was added to the mixture to reach a final concentration of 8 mM. The assembled complex was kept at room temperature until vitrification.

Cryo-EM specimen preparation and data collection. Samples were prepared for cryo-EM by applying 4 µl freshly purified *Asfv*Top2 or DNA/inhibitor *Asfv*Top2 complex (~0.3 mg/ml) to a glow-charged holey Au grid (Quantifoil R 2/2, 300 mesh, 25 mA for 40 s). The *apo* and the complex samples were respectively kept on ice and at room temperature (20 °C) for at least 30 mins before applying to the grids and vitrification. After sample application, the grids were incubated for 10 seconds and subsequently blotted with filter paper for 2-3 seconds at 10 °C in a 95 % humidity-controlled chamber, followed by plunge-freezing in liquid ethane using a FEI Vitrobot Mark IV (Thermo Fisher Scientific). Cryo-EM specimen grids were imaged on a FEI Titan Krios microscope (Thermo Fisher Scientific) operated at 300 kV. Dose-fractionated image stacks were recorded on a BioQuantum K3 Direct Electron Detector (Gatan) operating in super-resolution mode at 105,000x nominal magnification (corresponding to a pixel size of 0.415 Å/pixel. For the *apo*-*AsfvT*op2 and *Asfv*Top2:Cut02aDNA:etoposide (EDI-1) samples, 50 frames of non-gain normalized tiff stacks were recorded with a dose rate of ~14.0 $e^-/Å^2$ per second and the total exposure time was set to 3.5 s, resulting in an accumulated dose of ~ 50 $e^-/Å^2$ (~1 $e^-/Å^2$ per frame). For the *Asfv*Top2:Cut02bDNA:etoposide (EDI-2) and *Asfv*Top2:Cut02aDNA:*m*-AMSA (EDI-3) samples, 40 frames were recorded with a dose rate of ~16.8 $e^-/Å^2$ per second and the total exposure time was set to 2.5 s, resulting in an accumulated dose of ~42 $e^-/Å^2$ (~1 $e^-/Å^2$ per frame). The datasets were obtained with nominal defocus values ranging from 1.5 to 2.5 µm, and data collection parameters were controlled in an automated manner using EPU 2.10 (Thermo Fisher Scientific). No energy filter or objective aperture was implemented during data collection. The detailed statistics of individual datasets are summarized in **Tables 3** and **4**.

Cryo-EM Data processing and 3D reconstruction. All four datasets were collected in the super-resolution mode, the movies were binned twice during motion correction by MotionCor2[15], resulting in a pixel size of 0.83 Å/pixel for the final motion corrected images. The contrast transfer function (CTF) estimation was performed with cryoSPARC3.2[16]. For the apo-*Asfv*Top2, a dataset of 8,898 movies was collected. Initial particle picking was carried out using Blob picker in

cryoSPARC3.2[16] to obtain a small subset of particles for initial 2D classification. The good 2D classes were further used as a template for the entire particle picking by template picker in cryoSPARC3.2[16]. About 3.2 million particles were picked and extracted with a box size of 400 pixels for the following iterative 2D classification to eliminate bad particles. Around 2.6 million particles from good class averages were used to generate *de novo* initial 3D models (class=6, C1 symmetry) by cryoSPARC3.2[16], and followed by heterogeneous 3D refinement with C1 symmetry. Three models showing distinct features with better quality maps than the other three models were selected, and nearly 70 % of particles remained. The selected models were named as open form, closed form and the C-gate open form, which respectively possessed 9.7 %, 38.1 % and 22.2 % of the selected particles. The 251,335 particles accounting for the open form were subjected to 2D classification, homogeneous 3D refinement (C1 symmetry) and followed by 3D variability analysis. The two maps with the most obvious deviation from each other were selected and used as 3D templates for the following heterogeneous refinement (C1 symmetry) with the selected particles (n=155, 339). The resulting 3D models for the closed form are named conformer Ia and Ib with the resolution of 6.34 and 6.51 Å, respectively, which were further improved to 3.42 and 3.49 Å by non-uniform refinement with C2 symmetry. The same procedure was applied to the closed form and the C-gate open form. The final 3D models of the closed form conformers (IIa and IIb) calculated by homogeneous refinement (C2 symmetry) reach a resolution of 2.31 and 2.51 Å, respectively. For the C-gate open form, the final resulting resolution for its two conformers (IIIa and IIIb) are 2.43 and 2.69 Å, respectively (**Supplementary Fig. 1**).

For *Asfv*Top2-Cut02aDNA-etoposide, the initial particle picking followed the same procedure as described for the apo-*Asfv*Top2 dataset. There were approximately 2.8 million particles picked and extracted with a box size of 432 pixels by template picker from 11,786 micrographs. Initial iterations of 2D classification were conducted for filtering some junk particles. After multiple rounds of 2D classification, the retained particles were used to generate *de novo* initial 3D models (class=6, C1 symmetry) and followed by heterogeneous 3D refinement with C1 symmetry by cryoSPARC3.2[16]. Two classes with similar structural shapes to the known eukaryotic Top2 were selected and the particle numbers accounted for these two classes were approximately 60 % of the selected sets (n= ~1 million). The particles (n=632,027) accounted for the resulting 2 classes with improved features were selected for further non-uniform refinement with C2 symmetry. The resolution of the final resulting map (EDI-1) is 2.70 Å. Additionally, a subset of

particles (n=501,560) selected from the particles set (n=632,027) after 2D classification was subjected to generate *de novo* 3D models (class=4, C1 symmetry), and followed by heterogeneous 3D refinement with C1 symmetry. One class with defined shape of the full complex (EDI-1 full complex) was subjected to non-uniform refinement with C1 symmetry and resulted in the resolution of 3.68 Å (**Supplementary Fig. 2**).

For *Asfv*Top2-Cut02bDNA-etoposide, approximate 5.6 million particles were picked by template picking and extracted with a box size of 432 pixels from 10,919 micrographs. After iterations of 2D classification, the resulting particles (n=~0.8 millions) were subjected to generate *de novo* initial 3D models (class=6, C1 symmetry) by cryoSPARC3.2[16]. One class was expelled from the heterogeneous 3D refinement (C1 symmetry), and the remaining 88.0 % of particles were subjected to 2D classification to further eliminate junk particles. The final selected particles (n=650,266) and the best model with distinct features and higher resolution (5.74 Å) was subjected to non-uniform refinement with C2 symmetry. The resolution of the final 3D map (EDI-2) was improved to 2.74 Å (**Supplementary Fig. 3**).

For *Asfv*Top2-Cut02aDNA-*m*-AMSA, 9,846 micrographs were collected and an initial particle set (n= 5.4 millions) was obtained by the same procedure as described previously. After 2D classification, the selected particles (box size: 400 pixels, n= 1.4 millions) were subjected to generate *de novo* initial 3D models (class=6, C1 symmetry) and followed by heterogeneous 3D refinement with C1 symmetry by cryoSPARC3.2[16]. One class (n=351,979) showing distinct features with better map quality than the others was selected for non-uniform refinement in C2 symmetry. The resolution of the final 3D map (EDI-3) was improved to 3.0 Å (**Supplementary Fig. 4**).

The Fourier shell correlation (FSC) = 0.143 standard was used to estimate the overall resolution, and the local resolution was calculated by cryoSPARC3.2[16]. 3D variability analysis[17] was performed to obtain the 3D templates to further classify particles for reconstructing variable 3D volume models. UCSF Chimera v.1.15[18] was used to visualize the 3D density map.

Atomic modeling, refinement and validation. The initial templates used for modeling the *apo* structures conformers I and II were the crystal structure of the *E. coli* ParC (PDB 1ZVU[19]) and a predicted model obtained from Robetta online server[20], respectively. The conformer III was built using the conformer IIa as the initial model. For the three DNA/inhibitor bound structures (EDI-

1, -2, -3), the cleavage core domain was built based on the conformer IIa. The initial DNA template was derived from the human Top2 DNA complex[21] and then modified according to the Cut02a and Cut02b DNA sequences (**Fig. 3a**). The inhibitors were obtained from "Get monomer molecule" and manually modeled into the density in Coot v.0.8.8[9]. Densities were clearly evident with average resolutions of 2.7-3.0 Å (**Fig. 3b** and **Supplementary Fig. 1-3**) for most of the structure except for the ATPase domain and the linker regions. Additionally, we modeled the full length nucleoprotein complex using the crystal structure of the reduced *Asfv*Top2 ATPase/ MgAMP-PNP, and EDI-1 cryo-EM structure from this study. The linker region was mostly modeled as a loop conformation based on the prediction result and the available density (**Supplementary Fig. 15**). Initial rigid-body fitting for all structures was performed using "Fit in the Volume" function implemented in the UCSF Chimera v.1.15[18]. Further refinement cycles were performed using "phenix.real_space_refine" program in the Phenix ver.1.19 suite[22]. The atomic coordinates were rebuilt and fitted to the density map using Coot v.0.8.8[9]. Several iterations of real-space refinement on the entire model were completed by Phenix ver.1.19[22] until the refinement statistics converged. The final model was validated by MolProbity[10]. The detailed statistics of data processing, model refinement, and validation was listed in **Tables 2** and **3**. Structural visualization and rendering of structural representation were performed using PyMOL Molecular Graphics System, Version 2.4.1 Schrödinger, LLC and Chimera v.1.15[18].

Statistical analysis

Data analyses and presentation were performed using Microsoft Excel and Prism Version 9.5.0 (GraphPad Software, La Jolla, CA). Unpaired, two-tailed *t*-test was implemented to compare two groups. $P < 0.05$ was considered statistically significant. **Fig. 5c, d** were plotted using nonlinear regression fitting with error bar indicating 95 % confidence intervals based on technical triplicate (n=3).

Supplementary Note

Supplementary Note 1: Characterization of the preferential DNA sequences cleaved by *Asfv*Top2

The Asfv genome is known to feature terminal inverted repeats, cross links, and the hairpin loop structure at both ends[23]. The formation of the special head to head concatemeric genome intermediates during replication was reported for regeneration of the covalently closed ends[24]. These genome properties and significant *Asfv*Top2 sequence deviation from its eukaryotic and bacterial counterparts prompted us to examine the DNA cleavage specificity of this viral Top2. A graphical summary and detailed procedure for the characterization of the DNA sequences preferentially cleaved by *Asfv*Top2 are shown in **Supplementary Fig. 8**.

There were 117 clones obtained, which were classified based on the cutting positions. One unique cleavage position is defined with the same four bases at the cutting ends of both forward and reverse strands, since Top2 creates a double strand break with a four-base 5'-overhang at each cleaved strand[25,26]. Finally, 39 unique cleavage sites/sequences were characterized (**Supplementary Table 1**). Next, we calculated the frequency of the occurrence for the individual 39 unique sequences. Among them, the one (annotated as the Cut02, **Supplementary Table 1**), appeared most frequently and accounted for approximately 25 % of the sequenced clones, contrary to the rest of the cleavage sites with relatively less occurrence. Based on the frequency of the base occurrence at the individual position (**Supplementary Table 1**), we propose a consensus DNA sequence that is preferentially cleaved by *Asfv*Top2. As shown in **Supplementary Table 2**, the derived consensus sequence is a palindromic symmetry sequence flanking the two cleavage points on the double strand. The enzyme showed base preferences from the positions -1 to -5 and +5 to +9, but the bases were randomly distributed between positions +1 and +4. At position -1, the guanine is the most preferred base, and conversely adenine was absent at this particular position among the aligned sequences. This sequence preference of *Asfv*Top2 is somewhat different from that of Top2 from other species. As shown in **Supplementary Table 3**, the pyrimidines are the common bases in most eukaryotes at the position -1. The adenine is the preferred base at the positions -2 and -5 for *Asfv*Top2, whereas this tendency was unseen for the others. Likewise, *Asfv*Top2 preferred purines and pyrimidines at the positions -4 and +8, which is analogues to *Drosophila* and chicken, but not human proteins (**Supplementary Table 3**). Thus, *Asfv*Top2 harbors sequence preference that is somewhat different from other species. Nevertheless, we divided the most preferred DNA sequence (Cut02) into two doubly nicked 30 bp palindromic DNA segments (annotated as Cut02a and Cut02b, **Fig. 3a**) that respectively possesses the cleavage site sequence of the forward and reverse strands of the Cut02 (**Supplementary Table 1**).

Supplementary Note 2: Drug binding with *Asfv*Top2 and DNA

As shown in **Supplementary Fig. 10a**, two etoposide molecules are well resided at the two DNA nicks occurring in between the base pairs (-1/+5 and +1/+4) and forms stacking interactions with the +5 guanine and the -1 cytosine base, respectively. To accommodate the bulky ring structure of the inhibitor, the base pairing interaction of +1/+4 bases is disturbed, resulting in local deformation of the DNA (**Supplementary Fig. 10a)**. Both the glycosidic moiety and the pendant E-ring of the etoposide are the contact points with *Asfv*Top2. Particularly, the E-ring interacts with the adjacent D440 residue, and shows close contact (< 4 Å) with K417, E438, G439, G471, and G472 from the TOPRIM subdomain (**Supplementary Fig. 10b**), whereas the glycosidic moiety facing the major groove binding pocket merely interacts with T760 and shows close contact with M756.

Residing at the roughly similar position, *m*-AMSA has its acridine triple-ring core structure inserted in the space between the +1/+4 and -1/+5 base pairs and forms partial stacking interactions with the guanine base at the +1 and +5 positions, respectively (**Supplementary Fig. 10c**). Additional contacts between the inhibitor and DNA are formed in-between the anilino group of *m*-AMSA and the cytosine/guanine bases at +4/+5 positions. The methanesulfon moiety of the anilino group is in close distance (< 3.5 Å) pointing towards the sugar-phosphate backbone of the +5 guanine base (**Supplementary Fig. 10c**). The G471 forms a H-bond interaction with the N_{14} atom of *m*-AMSA. Residues, including L470, G472, V473, K503, and V504, are in the close contact (< 4 Å) with the inhibitor. The bulky methanesulfon-*m*-anisidide group of *m*-AMSA protrudes upwards, opposite side of the glycosidic moiety of etoposide is positioned **(Supplementary Fig. 10d)**. Therefore, the contact interface of *m*-AMSA is extended towards the minor groove-binding pocket with a few more residues from the TOPRIM subdomain, in comparison to the etoposide bound interface (**Supplementary Figs. 10b, d**).

*Asfv*Top2 possesses different drug-binding pockets from human Top2. Next, we compared the etoposide- and *m*-AMSA bound *Asfv*Top2 structures with the same drug-bound human Top2 structures[27,28]. In the etoposide-bound human Top2 structure, a conserved segment (PLR_{503}GKXL) in the TOPRIM subdomain was suggested to be essential in coordinating the etoposide binding. However, structure-based alignment shows the corresponding segment in *Asfv*Top2 is replaced by the segment: SLG_{471}GVXM, not conserved with the eukaryotic Top2 proteins (**Supplementary Fig. 5b**). Particularly, the human residue (R503) sidechain, forming key interactions with the

polycyclic ring system of the etoposide, is replaced by the G471 in *Asfv*Top2 (**Fig. 3g**). Even more variations are observed from the structural comparison of the *m*-AMSA binding pocket between the virus and human Top2 proteins (**Fig. 3h**). The human Top2 residues, interacting with the *m*-AMSA, are mostly non-conserved in *Asfv*Top2. Structural superposition of the *m*-AMSA bound viral and human Top2 structures highlights the distinctive residues from two species facilitating the drug binding **(Fig. 3h)**. For instances, the major interactions led by the sidechains of K503 and E522 in human Top2 are unseen in the viral protein, as these two polar residues are replaced by the non-polar G471 and V504. The other human Top2 residues, I454, P455, and A521, are replaced by D416, K417, and K503 in *Asfv*Top2, but merely K503 is involved in the drug binding.

Supplementary Note 3: Crystal structures of the *Asfv*Top2 ATPase domain in reduced and oxidized states

The ATP binding subdomain consists of the core elements of the Bergerat ATP-binding fold (**Supplementary Fig. 13a**), a unifying feature of the GHKL ATPase/kinase superfamily[29]. It is primarily constituted of an eight-stranded β-sheet and four α-helices (**Supplementary Fig. 6a**) that form the structural framework for ATP binding. These structural elements are connected via several surface loops that harbor the conserved residues coordinating the ATP binding. Of which, the loop (residues F_{125}- G_{147}) encompasses the conserved ATP-lid that completely encloses the bound MgAMP-PNP (**Supplementary Fig. 13b**). The residing residues, T130, N131, and $T_{143}NGVGLK_{149}$ show close contacts and interactions with the ribose and triphosphate moiety of AMP-PNP. Additionally, Q366 and K368 from an adjacent loop in the transducer subdomain form H-bonds with the *r*-phosphate. N64 and N95 interact with the α-phosphate and the N_6 atom of the adenine ring, respectively (**Supplementary Fig. 13b**). Unlike other homologs, the dimer interface of *Asfv*Top2 ATPase is predominantly constituted of the interactions between the N-terminal region, spanning approximately 30 residues, and the ATP-lid containing loop (**Supplementary Fig. 13c**). The main interactions are governed by the N-terminal loop. Sandwiched between the ATP-lid containing loop and the helix α4 from the other subunit, the N-terminal loop stabilizes the dimer assembly (**Supplementary Fig. 13b**). This structural feature is distinct from its eukaryotic and bacterial counterparts[6,30] that exhibit N-terminal α-helical turns prior to the equivalent loop region, leading to additional contacts in the dimer interface (**Supplementary Fig. 13c**). Overlap between the *Asfv*Top2 ATPase domain structure and its bacterial counterpart, ParE, from the *S.*

pneumoniae Top IV complexed with a 14-mer T-segment duplex DNA (PDB: 5J5Q)[30] shows the potential T-DNA segment binding interface and clashes between the DNA and a ß-hairpin loop (residue ranging V_{26}-D_{49}) from the viral protein (**Supplementary Fig. 13d**). Note that this long insertion hairpin loop is present in both viral and eukaryotic Top2 proteins, but not bacterial ones[6,30] (**Supplementary Fig. 13c**, **Supplementary Fig. 5a**). Immediately following the N-terminal loop, the ß-hairpin loop resides adjacent to the central cavity for trapping and passage of the T-DNA segment (**Supplementary Fig. 13c**). This structural arrangement likely couples the ATPase activity with the mechanistic manipulation of the T-DNA segment.

The crystal structure of the oxidized form was resolved at the same space group with a very high resolution (1.14 Å) from one single crystal. Superimposition of the reduced and oxidized forms showed marginal differences with an *r.m.s.d.* of 0.118 (332 Cα atoms/structure). The major difference is the formation of the disulfide bond between C72 and C138, which led to disruption of the interaction between C72 and H68, and partial deformation of helix α2 (**Fig. 5b**). Both C72 and H73 make significant relocation to interact with C138 and D137, respectively (**Fig. 5b**), which result in the covalent and hydrogen binding interactions with the long ATP-lid containing loop, that is positioned in between the helix α4 and α5 (**Supplementary Fig. 13b**, **Supplementary Fig. 5a**). The substantial local conformational change likely affects the hydrolytic activity of the enzyme via the ATP lid. Therefore, H68, C72, H73 and C138 were further subjected to mutational and functional analyses (**Supplementary Fig. 14**).

Supplementary Note 4: Modeling of the full-length *Asfv*Top2 DNA/inhibitor complex

We obtained the full electron density map (resolution 3.68 Å) of EDI-1 complex to include the ATPase domain and the linker region (**Supplementary Fig. 2h-m**, **Supplementary Fig. 15a**). The structures of the *Asfv*Top2 ATPase domain and the EDI-1 cleavage core domain were individually fitted into the density map. Whereas, the majority of *Asfv*Top2 linker region was predicted to form loop conformation[20], and unlike the helical linker resolved in the human Top2 cryo-EM structure[21]. The highly conserved motifs ($W_{414}XXFK_{418}$ and KKC_{427}) characterized in the eukaryotic Top2 linker[21], involved in coupling ATPase activity and DNA modulation are unseen in the *Asfv*Top2 (**Supplementary Fig. 15b**). The loop conformation imposes greater regional flexibility to some extent in comparison to the helical conformation, therefore the density was relatively poor to model the entire linker loop region (**Supplementary Fig. 15c**). Nevertheless, this structural feature

differentiates *Asfv*Top2 from the eukaryotic Top2, and implies that *Asfv*Top2 likely adapts a distinct approach to coordinate the essential functionalities occurring at the two distant catalytic sites.

Supplementary Figures

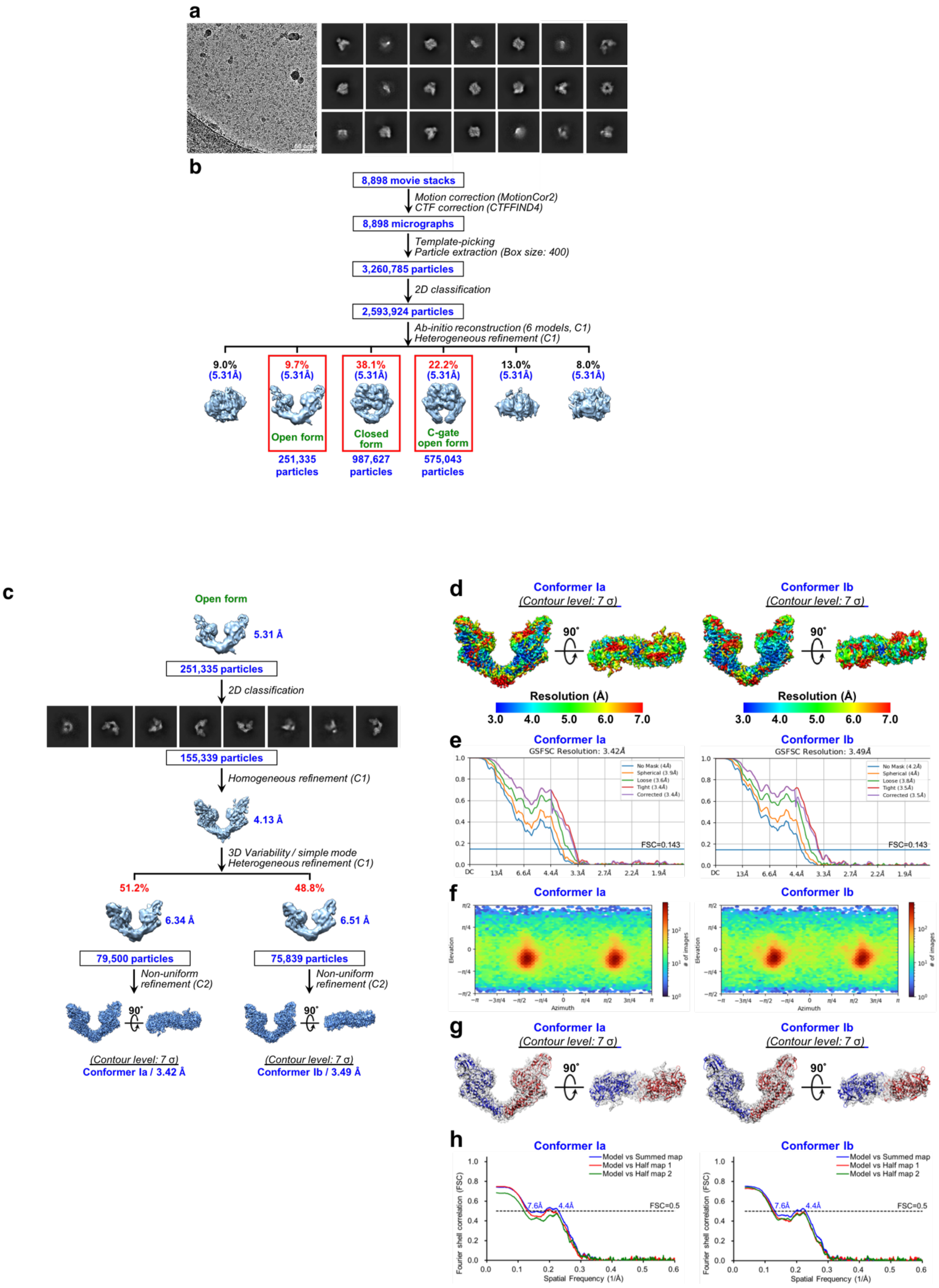

a
b
8,898 movie stacks
Motion correction (MotionCor2)
CTF correction (CTFFIND4)
8,898 micrographs
Template-picking
Particle extraction (Box size: 400)
3,260,785 particles
2D classification
2,593,924 particles
Ab-initio reconstruction (6 models, C1)
Heterogeneous refinement (C1)
9.0% (5.31Å)
9.7% (5.31Å)
38.1% (5.31Å)
22.2% (5.31Å)
13.0% (5.31Å)
8.0% (5.31Å)
Open form
Closed form
C-gate open form
251,335 particles
987,627 particles
575,043 particles
c
Open form
5.31 Å
251,335 particles
2D classification
155,339 particles
Homogeneous refinement (C1)
4.13 Å
3D Variability / simple mode
Heterogeneous refinement (C1)
51.2%
48.8%
6.34 Å
6.51 Å
79,500 particles
75,839 particles
Non-uniform refinement (C2)
Non-uniform refinement (C2)
90˚
90˚
(Contour level: 7 σ)
Conformer Ia / 3.42 Å
(Contour level: 7 σ)
Conformer Ib / 3.49 Å
d
Conformer Ia
(Contour level: 7 σ)
Conformer Ib
(Contour level: 7 σ)
90˚
90˚
Resolution (Å)
3.0 4.0 5.0 6.0 7.0
Resolution (Å)
3.0 4.0 5.0 6.0 7.0
e
Conformer Ia
GSFSC Resolution: 3.42Å
Conformer Ib
GSFSC Resolution: 3.49Å
FSC=0.143
FSC=0.143
f
Conformer Ia
Conformer Ib
Elevation
Azimuth
g
Conformer Ia
(Contour level: 7 σ)
Conformer Ib
(Contour level: 7 σ)
90˚
90˚
h
Conformer Ia
Conformer Ib
Model vs Summed map
Model vs Half map 1
Model vs Half map 2
7.6Å
4.4Å
FSC=0.5
Fourier shell correlation (FSC)
Spatial Frequency (1/Å)

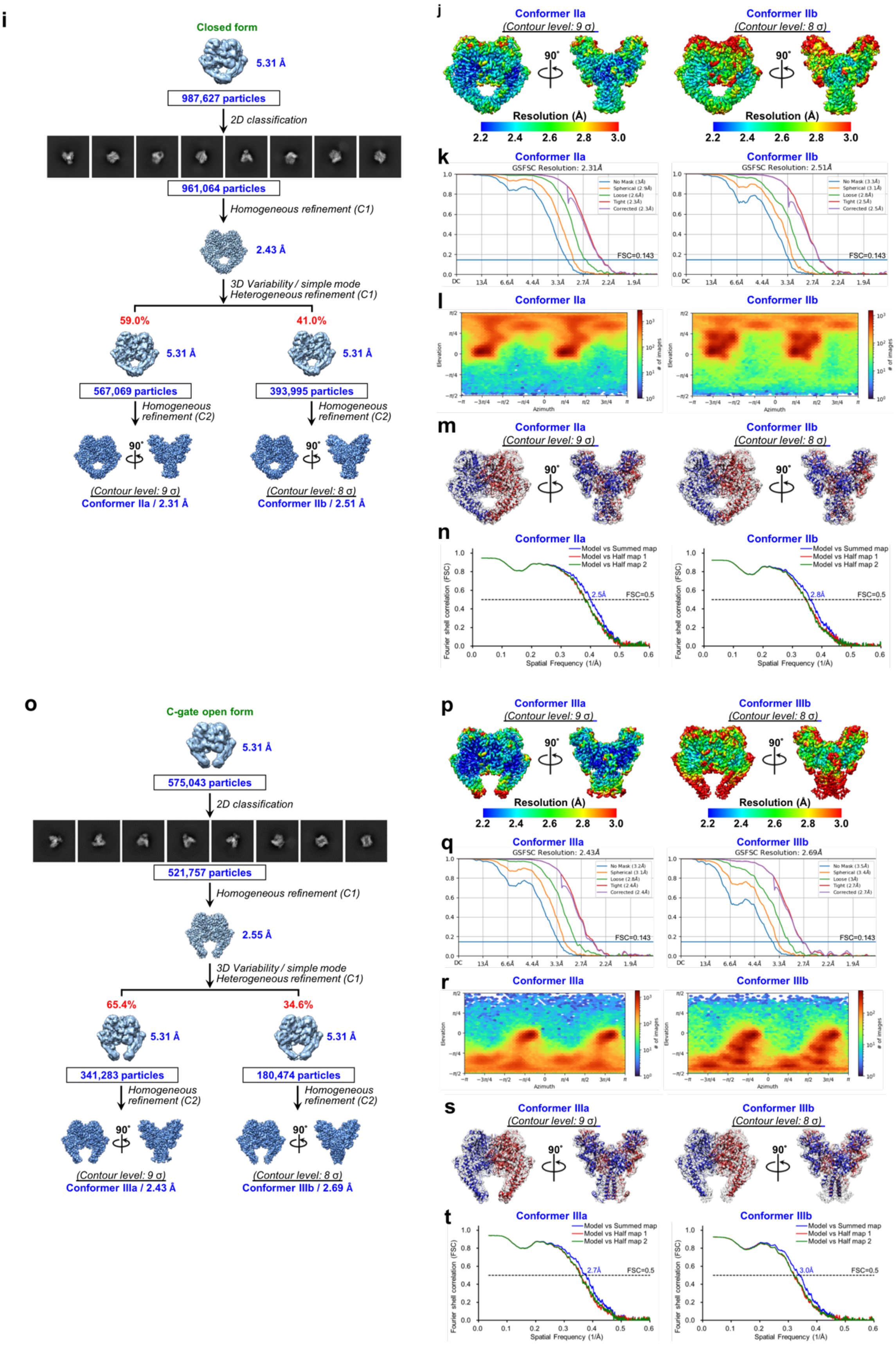
i
Closed form
5.31 Å
987,627 particles
2D classification
961,064 particles
Homogeneous refinement (C1)
2.43 Å
3D Variability / simple mode
Heterogeneous refinement (C1)
59.0%
41.0%
5.31 Å
5.31 Å
567,069 particles
393,995 particles
Homogeneous refinement (C2)
Homogeneous refinement (C2)
90°
90°
(Contour level: 9 σ)
Conformer IIa / 2.31 Å
(Contour level: 8 σ)
Conformer IIb / 2.51 Å
j
Conformer IIa
(Contour level: 9 σ)
Conformer IIb
(Contour level: 8 σ)
90°
Resolution (Å)
2.2 2.4 2.6 2.8 3.0
k
Conformer IIa
GSFSC Resolution: 2.31Å
Conformer IIb
GSFSC Resolution: 2.51Å
FSC=0.143
l
Conformer IIa
Conformer IIb
Elevation
Azimuth
m
Conformer IIa
(Contour level: 9 σ)
Conformer IIb
(Contour level: 8 σ)
n
Conformer IIa
Conformer IIb
Model vs Summed map
Model vs Half map 1
Model vs Half map 2
2.5Å
2.8Å
FSC=0.5
Fourier shell correlation (FSC)
Spatial Frequency (1/Å)
o
C-gate open form
5.31 Å
575,043 particles
2D classification
521,757 particles
Homogeneous refinement (C1)
2.55 Å
3D Variability / simple mode
Heterogeneous refinement (C1)
65.4%
34.6%
5.31 Å
5.31 Å
341,283 particles
180,474 particles
Homogeneous refinement (C2)
Homogeneous refinement (C2)
90°
90°
(Contour level: 9 σ)
Conformer IIIa / 2.43 Å
(Contour level: 8 σ)
Conformer IIIb / 2.69 Å
p
Conformer IIIa
(Contour level: 9 σ)
Conformer IIIb
(Contour level: 8 σ)
90°
Resolution (Å)
2.2 2.4 2.6 2.8 3.0
q
Conformer IIIa
GSFSC Resolution: 2.43Å
Conformer IIIb
GSFSC Resolution: 2.69Å
FSC=0.143
r
Conformer IIIa
Conformer IIIb
Elevation
Azimuth
s
Conformer IIIa
(Contour level: 9 σ)
Conformer IIIb
(Contour level: 8 σ)
t
Conformer IIIa
Conformer IIIb
Model vs Summed map
Model vs Half map 1
Model vs Half map 2
2.7Å
3.0Å
FSC=0.5
Fourier shell correlation (FSC)
Spatial Frequency (1/Å)

Supplementary Fig. 1. Cryo-EM structure determination of the *apo-Asfv*Top2. a. Representative motion-corrected cryo-electron micrograph (left) and the representative 2D class averages (right) indicate the quality and orientations of particles. **b.** The flow chart for the cryo-EM data processing. **c.** The flow chart for the cryo-EM data processing of the conformer I (open). **d.** Local resolution analyses for the 3D maps of the conformer Ia and Ib. The maps are colored based on the local resolution. **e.** Gold-standard FSC curves (FSC= 0.143) of conformer Ia and Ib. The unusual dip of the curves is likely due to the reduced number of views and intrinsic dynamic nature in comparison to the other conformer populations. We are confident in their reliability since the tight and corrected FSC curves (red and purple, respectively) agree at higher resolutions in both cases, suggesting that the masking did not cause any resolution artefacts. In support, the cross-validation FSC curves of the model vs. map were closely superposed (see panel h below), assuring no significant overfitting. **f.** The angular distributions were calculated for all particle projections in the final 3D reconstructions of conformer Ia and Ib. The heat maps show the number of particles for each viewing angle. The coloring scheme represents the population of particle distribution. **g.** The cryo-EM density maps (in transparent gray) of the conformer Ia and Ib for building 3D models. The dimer subunits are colored in red and blue, respectively. **h.** Map versus model FSC curves were calculated between molecular models and the corresponding full summed map (blue), half map 1 (red, used for refinement) and the half map 2. **i.** The flow chart for the cryo-EM data processing of the closed form (conformer II). **j.** Local resolution analysis for the 3D maps. The maps are colored according to the local resolution. **k.** Gold-standard FSC curves (FSC= 0.143). **l.** The angular distributions calculated in cryoSPARC3.2[16] for all particle projections in the final 3D reconstructions. The heat maps show the number of particles for each viewing angle. The coloring scheme represents the population of particle distribution. **m.** The final cryo-EM maps (in transparent gray) of conformer IIa and IIb, built with individual molecular model. The dimer subunits are presented in red and blue. **n.** Map versus model FSC curves were calculated between molecular models and the corresponding full summed map (blue), half map 1 (red, used for refinement) and the half map 2. **o.** The flow chart for the cryo-EM data processing of the C-gate open form (conformer III). **p.** Local resolution analyses of the 3D maps. The cryo-EM maps are coloured according to the local resolution. **q.** Gold-standard FSC curves (FSC= 0.143). **r.** The angular distributions calculated in cryoSPARC3.2[16] for all particle projections in the final 3D reconstructions. The heat maps show the number of particles for each viewing angle. **s.** The cryo-EM density maps (in transparent gray) of and their corresponding molecular models. The dimer subunits are presented in red and blue. **t.** Map versus model FSC curves were calculated between molecular models and the corresponding full summed map (blue), half map 1 (red, used for refinement) and the half map 2.

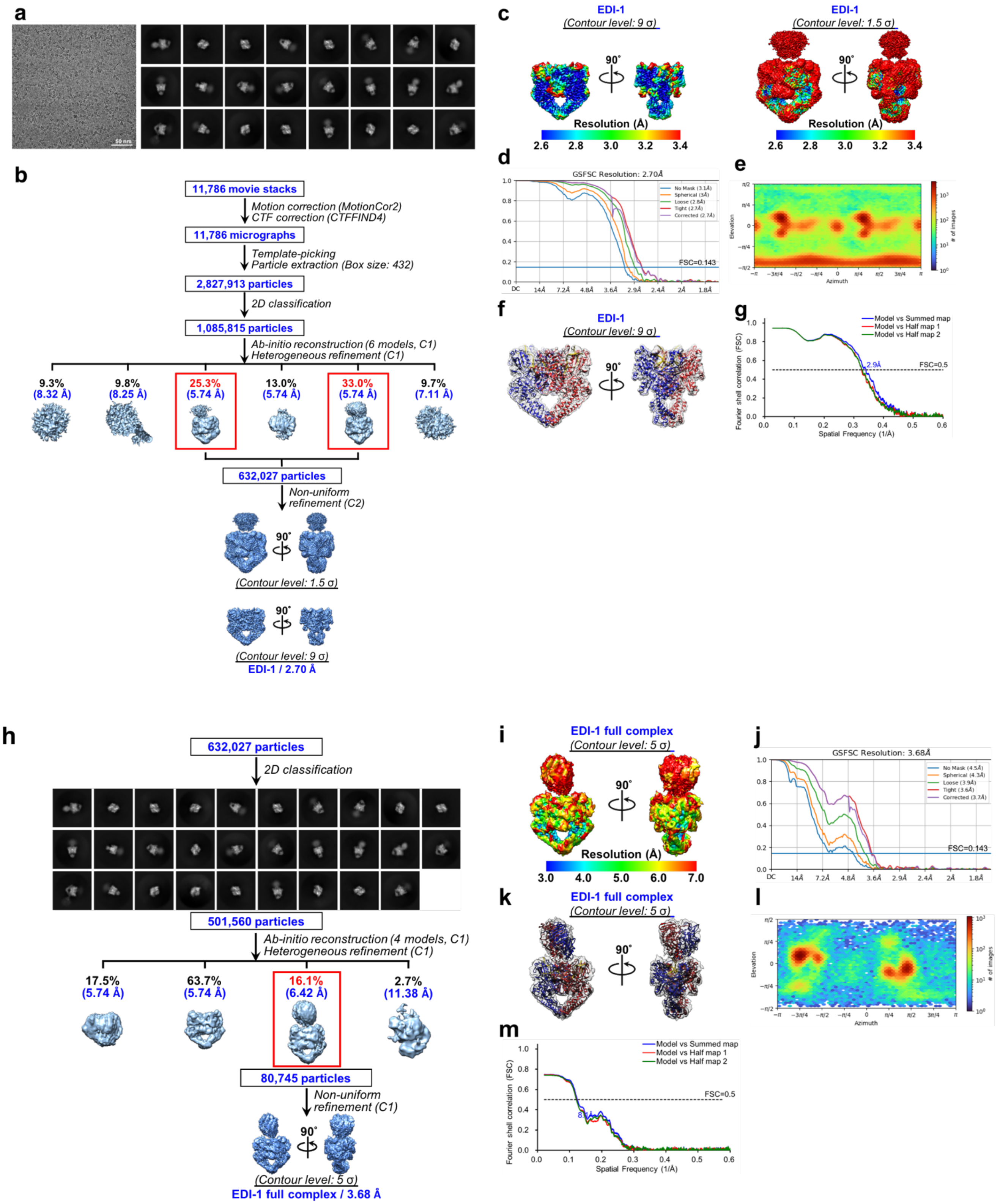
a
50 nm
b
11,786 movie stacks
Motion correction (MotionCor2)
CTF correction (CTFFIND4)
11,786 micrographs
Template-picking
Particle extraction (Box size: 432)
2,827,913 particles
2D classification
1,085,815 particles
Ab-initio reconstruction (6 models, C1)
Heterogeneous refinement (C1)
9.3% (8.32 Å)
9.8% (8.25 Å)
25.3% (5.74 Å)
13.0% (5.74 Å)
33.0% (5.74 Å)
9.7% (7.11 Å)
632,027 particles
Non-uniform refinement (C2)
90°
(Contour level: 1.5 σ)
(Contour level: 9 σ)
EDI-1 / 2.70 Å
c
EDI-1
(Contour level: 9 σ)
EDI-1
(Contour level: 1.5 σ)
Resolution (Å)
2.6 2.8 3.0 3.2 3.4
d
GSFSC Resolution: 2.70Å
No Mask (3.1Å)
Spherical (3Å)
Loose (2.8Å)
Tight (2.7Å)
Corrected (2.7Å)
FSC=0.143
e
Elevation
Azimuth
# of images
f
EDI-1
(Contour level: 9 σ)
g
Model vs Summed map
Model vs Half map 1
Model vs Half map 2
2.9Å
FSC=0.5
Fourier shell correlation (FSC)
Spatial Frequency (1/Å)
h
632,027 particles
2D classification
501,560 particles
Ab-initio reconstruction (4 models, C1)
Heterogeneous refinement (C1)
17.5% (5.74 Å)
63.7% (5.74 Å)
16.1% (6.42 Å)
2.7% (11.38 Å)
80,745 particles
Non-uniform refinement (C1)
(Contour level: 5 σ)
EDI-1 full complex / 3.68 Å
i
EDI-1 full complex
(Contour level: 5 σ)
Resolution (Å)
3.0 4.0 5.0 6.0 7.0
j
GSFSC Resolution: 3.68Å
No Mask (4.5Å)
Spherical (4.3Å)
Loose (3.9Å)
Tight (3.6Å)
Corrected (3.7Å)
FSC=0.143
k
EDI-1 full complex
(Contour level: 5 σ)
l
Elevation
Azimuth
# of images
m
Model vs Summed map
Model vs Half map 1
Model vs Half map 2
FSC=0.5
Fourier shell correlation (FSC)
Spatial Frequency (1/Å)

Supplementary Fig. 2. Cryo-EM structure determination of the *Asfv*Top2-Cut02aDNA-etoposide (EDI-1). a. Representative motion-corrected cryo-electron micrograph (left) and the representative 2D class averages (right) indicate the quality and orientations of particles. **b.** The flow chart for the cryo-EM data processing of EDI-1 complex. **c.** Local resolution analysis of EDI-1 3D map, shown at higher (left) and lower (right) contour levels. The cryo-EM map is colored according to the local resolution. **d.** Gold-standard FSC curve (FSC= 0.143). **e.** The angular distribution is calculated in cryoSPARC3.2[16] for all particle projections for the final 3D reconstruction of EDI-1. The heat map shows the number of particles for each viewing angle. The coloring scheme represents the population of particle distribution. **f.** The cryo-EM density map (in transparent gray) of EDI-1 and its 3D model. The dimer subunits are colored in red and blue. The DNA is colored in yellow. **g.** Map versus model FSC curves were calculated between the molecular model and the corresponding full summed map (blue), half map 1 (red, used for refinement) and half map 2. **h.** The data processing flow chart of the EDI-1 full complex, including the ATPase and linker regions. To obtain the full reconstruction of the EDI-1 complex including the flexible linker region and ATPase domain, we further conducted 2D classification of the particle population (n=632,027 from panel b) and selected classes with the views of the full EDI-1 complex. The resulting particles (n=501,560) were further subjected to *ab-initio* reconstruction into 4 models. Of which, the 3D volume model with the full view including the ATPase domain was made by merely 16.1% of the total particle population, which was then refined to 3.68 Å resolution with C1 symmetry. **i.** Local resolution analysis for the 3D map. The cryo-EM map is coloured according to the local resolution. **j.** Gold-standard FSC curve (FSC= 0.143). The unusual dip of the curves is likely due to the small number of the particle views mentioned above, and the intrinsic dynamic nature of the ATPase domain and linker region. As also explained in **Supplementary Fig. 1e**, we are confident in their reliability since the tight and corrected FSC curves (red and purple, respectively) agree at higher resolutions in both cases, suggesting that the masking did not cause any resolution artefacts. In support, the cross-validation FSC curves of the model vs. map were closely superposed (see panel m below), assuring no significant overfitting. **k.** The cryo-EM density map (in transparent gray) of the full complex for building its molecular model. The subunits are present in red and blue, with DNA colored in yellow. **l.** The angular distribution is calculated by cryoSPARC3.2[16] for all particle projections in the final 3D reconstruction. The heat map shows the number of particles for each viewing angle. The coloring scheme represents the population of particle distribution. **m.** Map versus model FSC curves were calculated between the molecular model and the corresponding full summed map (blue), half map 1 (red, used for refinement) and the half map 2.

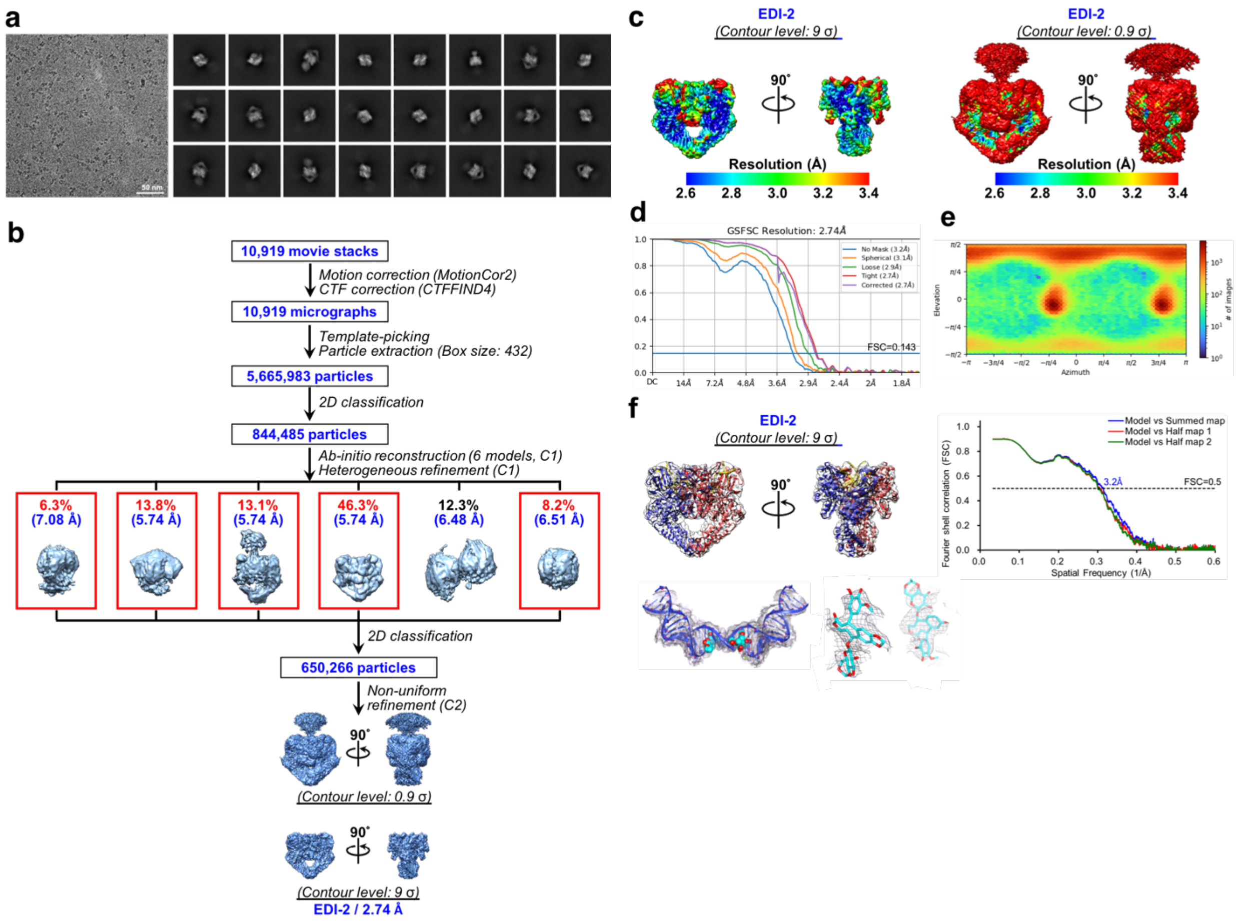

Supplementary Fig. 3. Cryo-EM structure determination of the *Asfv*Top2-Cut02bDNA-etoposide (EDI-2). a. Representative motion-corrected cryo-electron micrograph (left) and the representative 2D class averages (right) of *Asfv*Top2-Cut02bDNA-etoposide indicate the quality and orientations of particles. **b.** The flow chart for the data processing of the EDI-2. **c.** Local resolution analysis of the 3D map shown at higher (left) and lower (right) contour level. The cryo-EM map is colored according to the local resolution. **d.** Gold-standard FSC curve (FSC= 0.143) **e.** The angular distribution calculated in cryoSPARC3.2[16] for all particle projections in the final 3D reconstruction. The heat map shows the number of particles for each viewing angle. The coloring scheme represents the population of particle distribution. **f.** The cryo-EM density map (in transparent gray) is built with the final refined model. The dimer subunits are present in red and blue. The density (contoured at 0.151, UCSF Chimera v.1.15[18]) for modeling Cut02bDNA, and etoposide. **g.** Map versus model FSC curves were calculated between molecular model and the corresponding full summed map (blue), half map 1 (red, used for refinement) and half map 2.

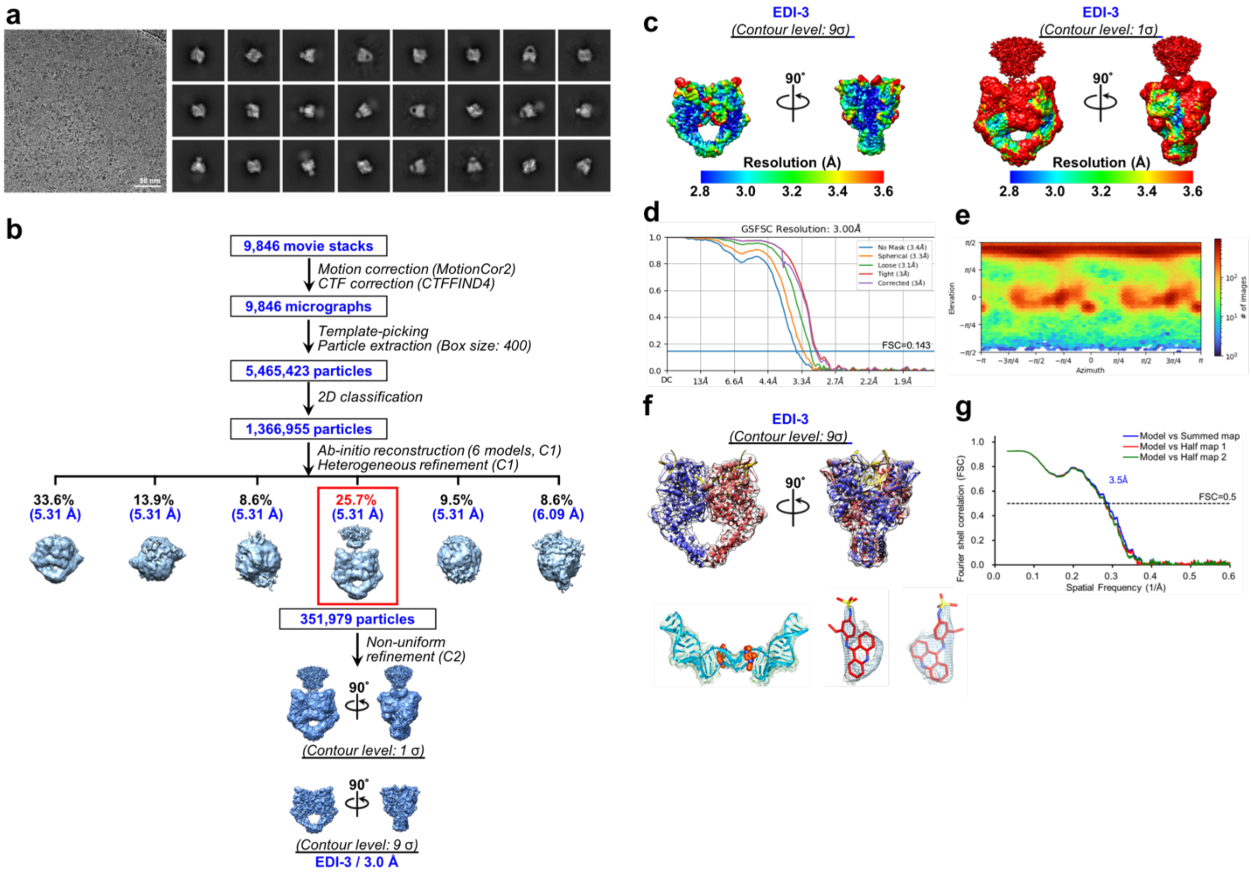

Supplementary Fig. 4. Cryo-EM structure determination of the *Asfv*Top2-Cut02aDNA-*m*-AMSA(EDI-3). a. Representative motion-corrected cryo-electron micrograph (left) and the representative 2D class averages (right) show the quality and orientations of particles. **b.** The flow chart for the cryo-EM data processing. **c.** Local resolution analysis of the cryo-EM map, shown at higher (left) and lower (right) contours and colored according to the local resolution. **d.** Gold-standard FSC curve (FSC= 0.143). **e.** The angular distribution for all particle projections in the final 3D reconstruction. The heat map shows the number of particles for each viewing angle. The coloring scheme represents the population of particle distribution. **f.** The cryo-EM density map (in transparent gray) is built with the final refined model. The dimer subunits are colored in red and blue. The density (contoured at 0.151, UCSF Chimera v.1.15[18]) for modeling Cut2aDNA, and *m*-AMSA. **g.** Map versus model FSC curves were calculated between molecular model and the corresponding full summed map (blue), half map 1 (red, used for refinement) and the half map 2.

a

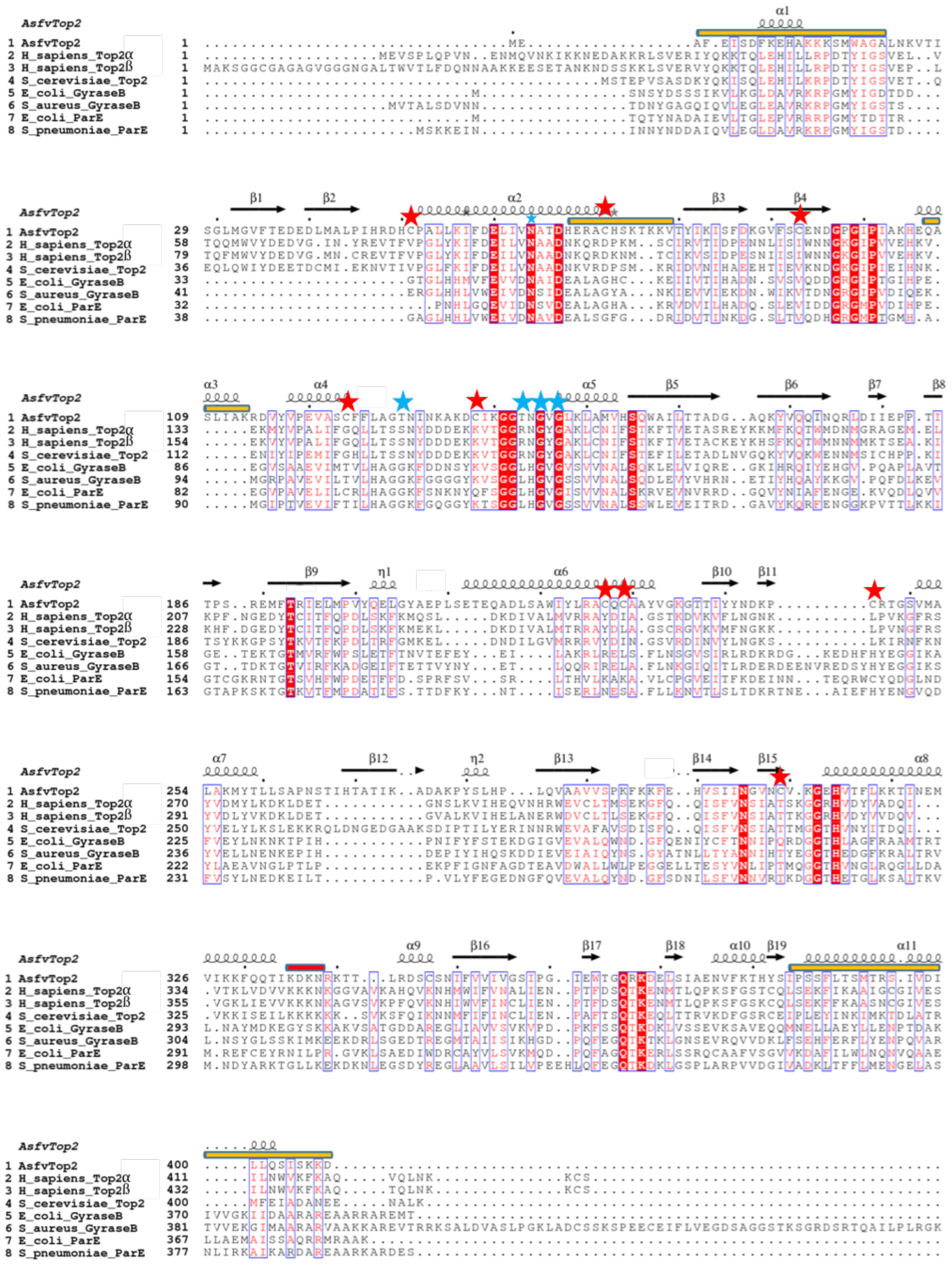
AsfvTop2
1 AsfvTop2
2 H_sapiens_Top2α
3 H_sapiens_Top2β
4 S_cerevisiae_Top2
5 E_coli_GyraseB
6 S_aureus_GyraseB
7 E_coli_ParE
8 S_pneumoniae_ParE

```
   AsfvTop2

1 AsfvTop2                    ................................................................................
2 H_sapiens_Top2α             ................................................................................
3 H_sapiens_Top2ß             ................................................................................
4 S_cerevisiae_Top2           ................................................................................
5 E_coli_GyraseB              ................................................................................
6 S_aureus_GyraseB   461      ILNVEKARLDRILNNNEIRQMITAFGTGIGGDFDLAKARYHKIVIMTDADVDGAHIRTLLLTFFYRFMRPLIEAGYVYIA
7 E_coli_ParE                 ................................................................................
8 S_pneumoniae_ParE           ................................................................................
```

```
   AsfvTop2

1 AsfvTop2                    ................................................................................
2 H_sapiens_Top2α             ................................................................................
3 H_sapiens_Top2ß             ................................................................................
4 S_cerevisiae_Top2           ................................................................................
5 E_coli_GyraseB              ................................................................................
6 S_aureus_GyraseB   541      QPPLYKLTQGKQKYYVYNDRELDKLKSELNPTPKWSIARYKGLGEMNADQLWETTMNPEHRALLQVKLEDAIEADQTFEM
7 E_coli_ParE                 ................................................................................
8 S_pneumoniae_ParE           ................................................................................
```

```
   AsfvTop2

1 AsfvTop2                    ........................
2 H_sapiens_Top2α             ........................
3 H_sapiens_Top2ß             ........................
4 S_cerevisiae_Top2           ........................
5 E_coli_GyraseB              ........................
6 S_aureus_GyraseB   621      LMGDVVENRRQFIEDNAVYANLDF
7 E_coli_ParE                 ........................
8 S_pneumoniae_ParE           ........................
```

b

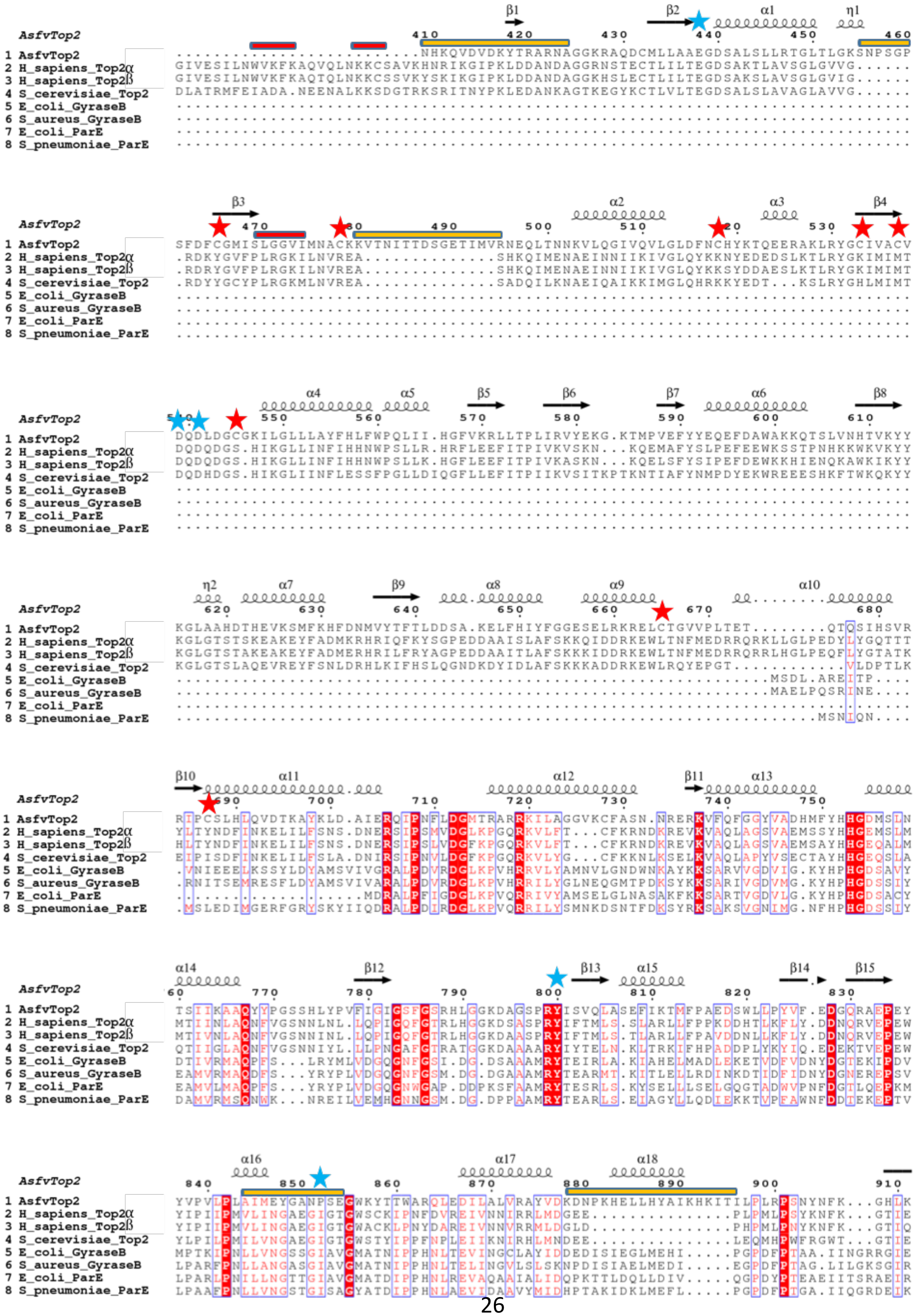
AsfvTop2
1 AsfvTop2
2 H_sapiens_Top2α
3 H_sapiens_Top2β
4 S_cerevisiae_Top2
5 E_coli_GyraseB
6 S_aureus_GyraseB
7 E_coli_ParE
8 S_pneumoniae_ParE

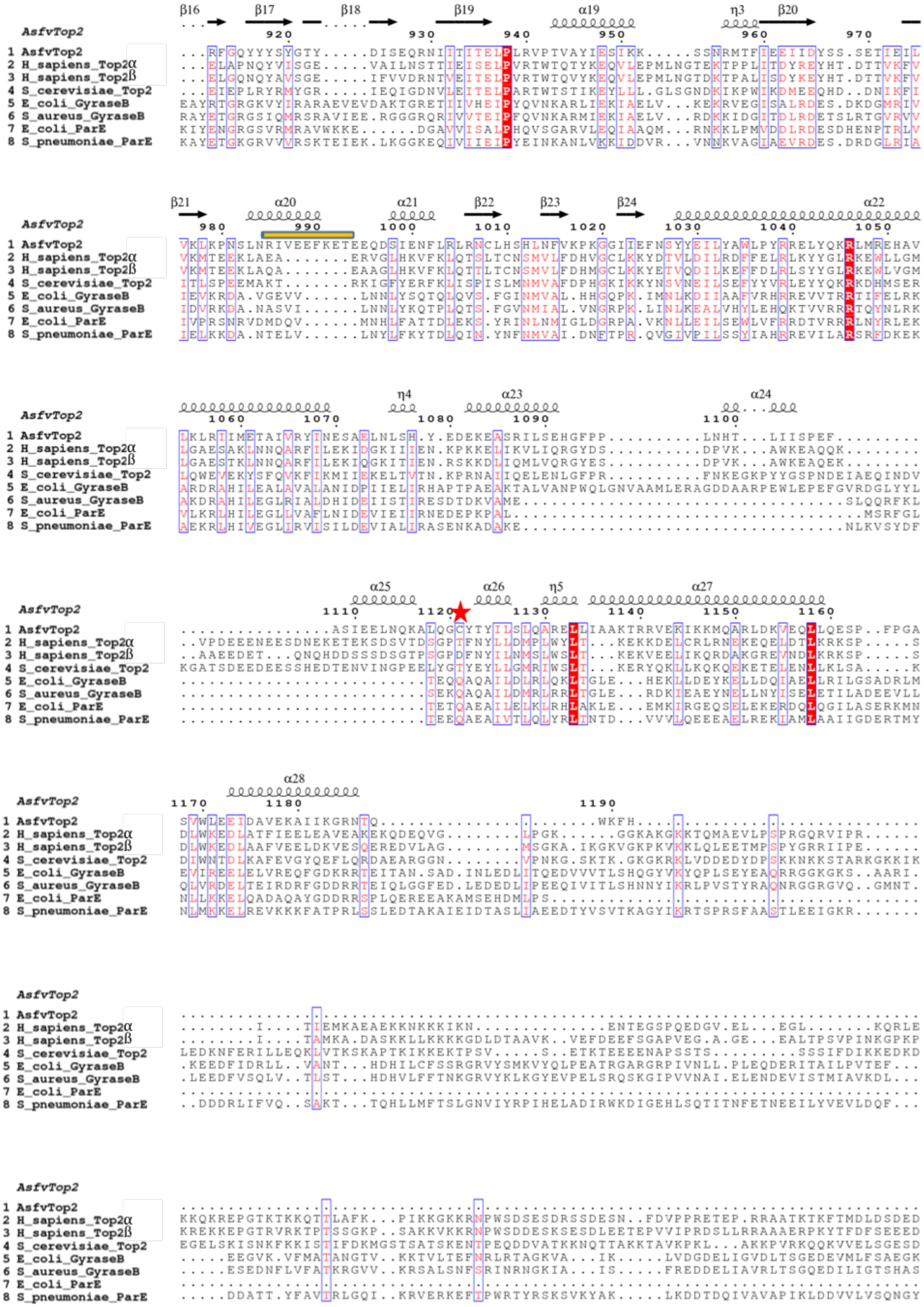
AsfvTop2
1 AsfvTop2
2 H_sapiens_Top2α
3 H_sapiens_Top2β
4 S_cerevisiae_Top2
5 E_coli_GyraseB
6 S_aureus_GyraseB
7 E_coli_ParE
8 S_pneumoniae_ParE

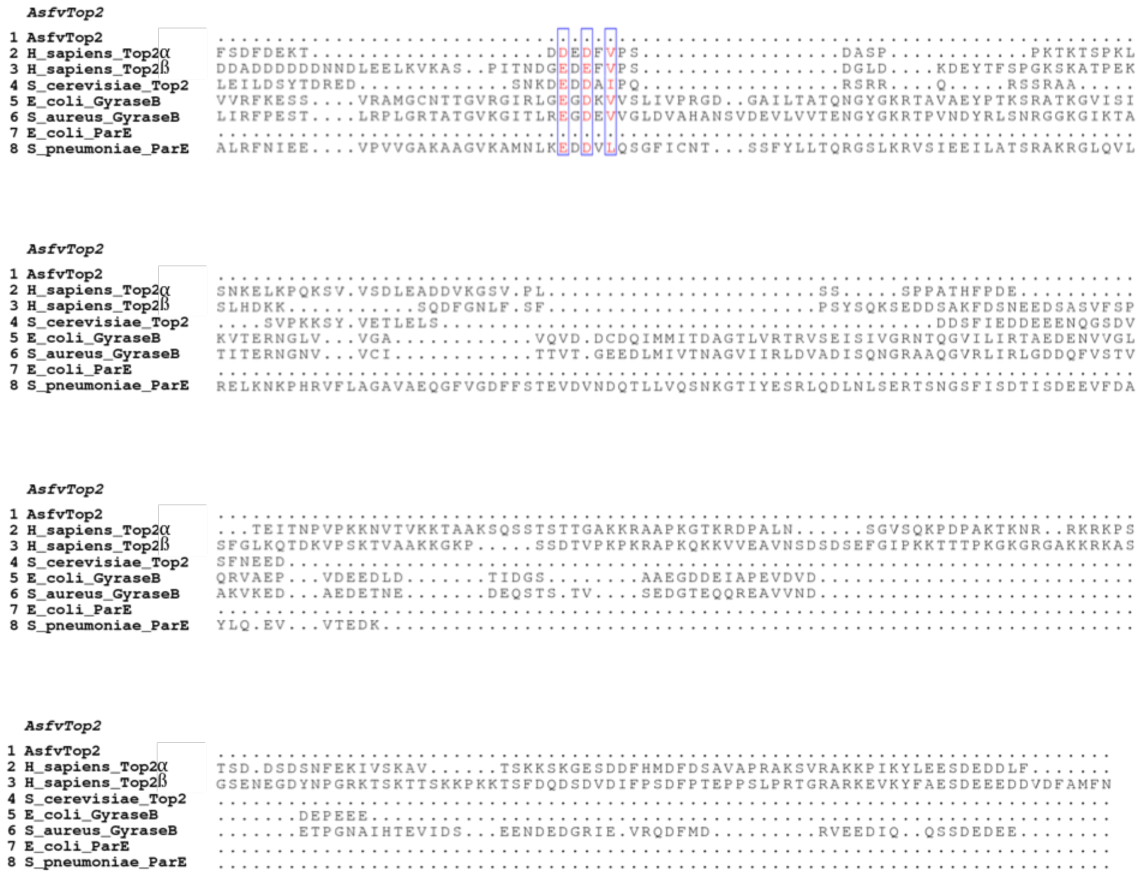

Supplementary Fig. 5. Sequence alignment of *Asfv*Top2 with eukaryotic and bacterial homologs. a. ATPase domain. **b**. DNA binding/cleavage domain. The aligned Top2 protein sequences are: 1. *Asfv*Top2 (UniProt: Q00942), 2. *H. sapiens* Top2α (UniProt: P11388), 3. *H. sapiens* Top2ß (UniProt: Q02880), 4. *S. cerevisiae* Top2 (UniProt: P06786), 5. *E. coli* Gyrase B (Uniprot: P0AES6)/Gyrase A (UniProt: P0AES4), 6. *E. coli* ParE (UniProt: P20083)/ParC (UniProt: P0AFI2), 7. *S. pneumoniae* ParE (UniProt: Q59961)/ParC (UniProt: P72525), 8. *S. aureus* Gyrase B (UniProt: P66937)/Gyrase A (UniProt: Q99XG5). Regions highlighted with red bars are the conserved and in relevant to functions, including the k-loop[31], linker motifs[21] and etoposide binging motif [27]. The conserved catalytic residues are marked with blue stars. The highly non-conserved or insertion regions of *Asfv*Top2 are marked with orange bars. The non-conserved cysteine and the DNA intercalating residues are marked with red stars. The input alignment file was prepared using Clustal Omega[32]. The figure was the output from ESPript3[33] with default setting.

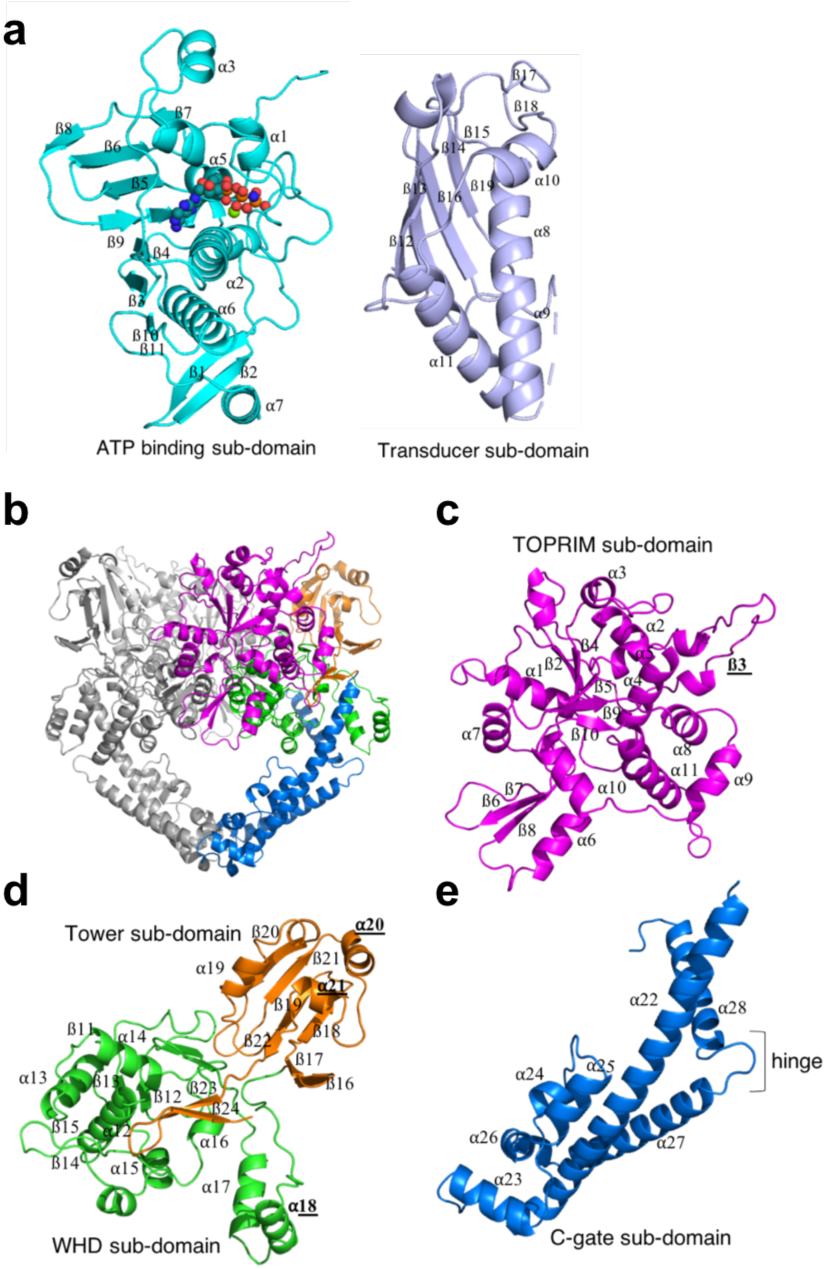

Supplementary Fig. 6. Secondary structure annotation of the *Asfv*Top2. a. ATPase domain. The input structure files were the crystal structure of *Asfv*Top2 ATPase domain in the reduced state (resolution 1.73 Å), and **b**. the cryo-EM structure of *apo*-*Asfv*Top2 conformer IIa (resolution 2.31 Å) determined in this study. The individual subdomain is colored based on the scheme in **Fig. 1a**. The DNA and etoposide are omitted for clarity. **c.** The TOPRIM subdomain, **d.** Tower and WHD, and **e.** C-gate subdomains. The annotation are based on the ESPript3[33] output.

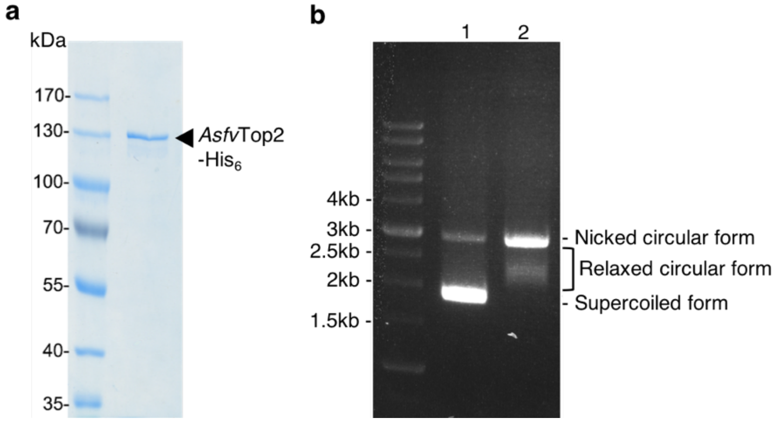

Supplementary Fig. 7. Protein purification of full length AsfvTop2 and DNA relaxation assays. a. Representative SDS-PAGE (7.5 %) indicating the molecular size and the purity of the full-length *Asfv*Top2. **b.** The SYBR^{TM}-DNA stained agarose gel (1.0 %) showing DNA relaxation activity of the enzyme. Lane1: pUC19 in supercoiled form. Lane2: pUC19 was converted to the relaxed and nicked circular forms upon addition of *Asfv*Top2 (**Supplementary Data 1**).

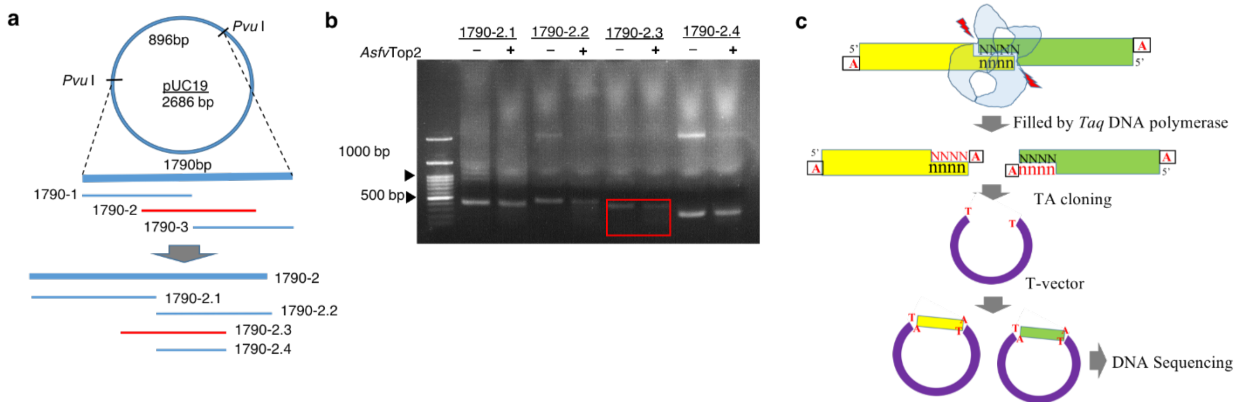

Supplementary Fig. 8. Characterization of the preferential DNA sequences cleaved by *Asfv*Top2. a. Schematic representation of hierarchical fragmentation of the pUC19 plasmid used in *Asfv*Top2 cleavage assay. pUC19 was cut with PvuI into two large DNA fragments, 896 bp and 1790 bp. Other fragments, 1790-n and 1790-n.n, were obtained from PCR with related primers. The red color fragment represents the chosen one in each hierarchical step. **b.** The SYBR^{TM}-DNA stained agarose gel (1.0 %) showing further cleavage of the sub-DNA fragments by *Asfv*Top2 in 6 min. We found that all of them could be cleaved with similar efficiency (compare – and + lanes for 1790-2.1, 2.2, 2.3, and 2.4), suggesting that the specificity of *Asfv*Top2 is not very high. We decided that any one of these fragments can be used for further systematic analysis in (c), which will go through randomization processes to provide fine-tuned specificity. The fragment 1790-2.3 (red square) was chosen since its DNA appeared more pure (less contamination of larger fragments), and it is indeed cleaved by *Asfv*Top2. **c.** The *Asfv*Top2 cleaved, smearing DNA fragments in the region below the 398 bp 1790-2.3 fragment were cut and eluted from agarose gel. The eluate was treated with *Taq* polymerase (New England Biolabs) to fill up the 5’ overhang end with 5 bases. The reactions resulted in the 3’-end of both forward and revere strands with an adenine for cloning to a T-overhang vector. The resulted clones were subjected to DNA sequencing to obtain the cutting sequences recognized by *Asfv*Top2.

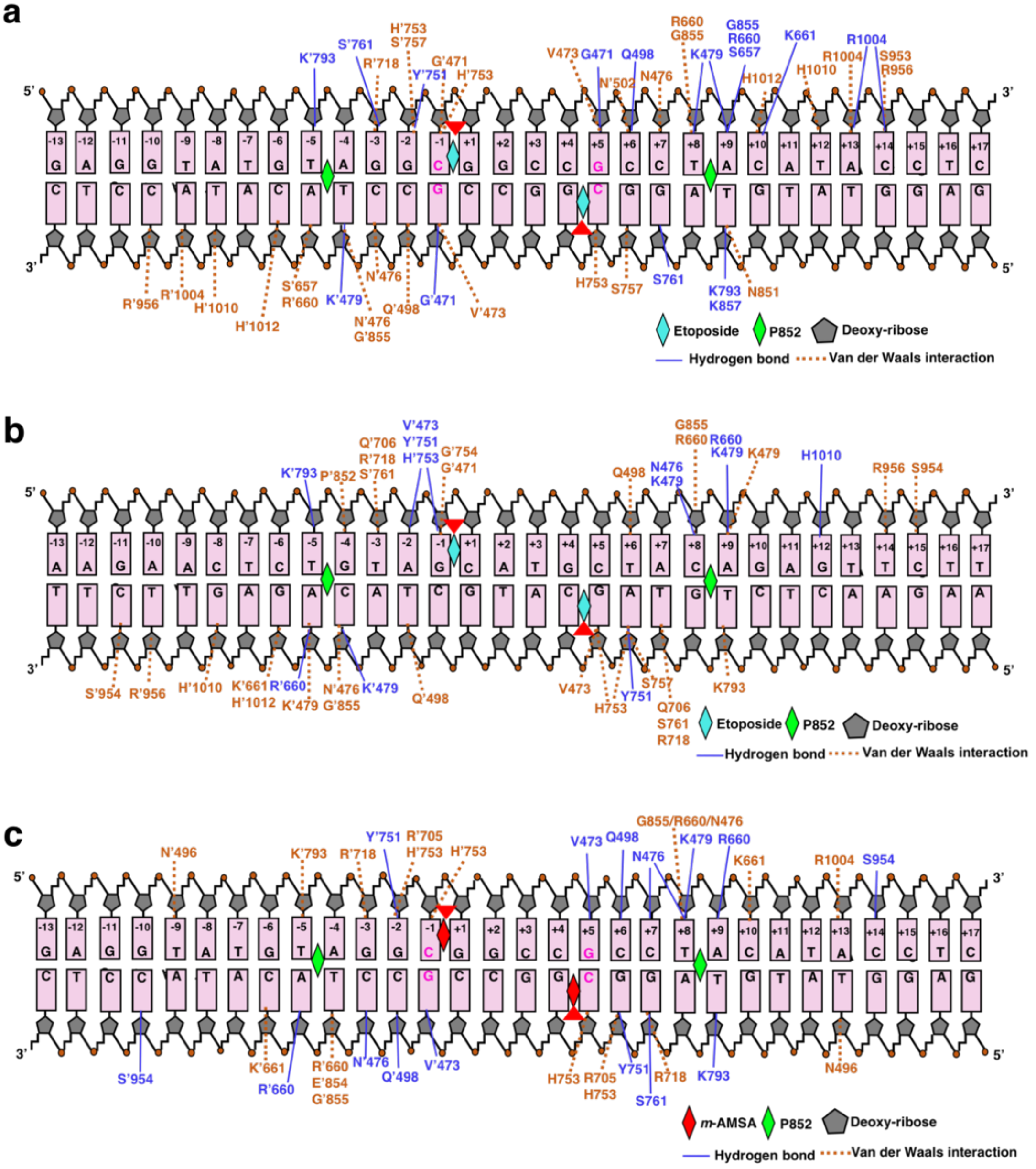

Supplementary Fig. 9. Cryo-EM structure of the full-length *Asfv*Top2 complexed with Cut02b DNA/etoposide or Cut02a DNA/ *m*-AMSA. a. b. c. Schematic diagrams of the protein-Cut02a/02b DNA interactions with etoposide/ *m*-AMSA bound. The labelled residues from the other monomer is flagged by a prime. Blue and orange colored residues form H-bonds and Van der Waals interactions with DNA, respectively. The *m*-AMSA and etoposide are highlighted in red and cyan diamond shape. The position of P852 and DNA nick are indicated by green diamond shapes and red triangles, respectively.

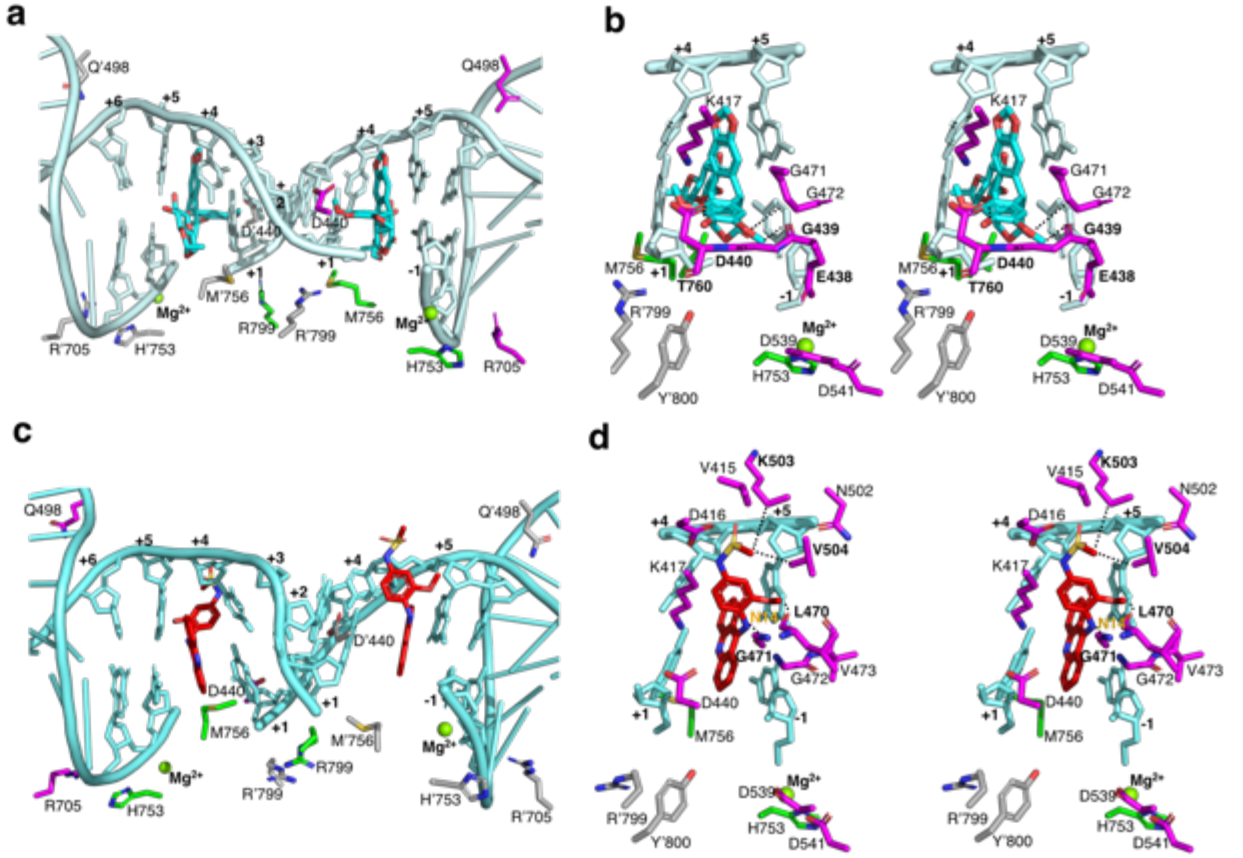

Supplementary Fig. 10. Drug binding pockets in *Asfv*Top2 and key residues involved in drug/DNA interactions. a. c. The two drug molecules (etoposide in cyan stick presentation; *m*-AMSA in red) reside at the close proximity to the DNA nicks and the catalytic sites of *Asfv*Top2 cleavage core domain. The residues shown in stick are in close contact (< 5 Å) with DNA. Colored scheme is based on **Fig. 1a**. **b, d.** Stereo views of the etoposide/*m*-AMSA binding pockets. The labelled residues surrounding the drug are the contacting interface residues. Residues labelled in bold form close contacts with the drug. Contact distances < 4 Å are labeled with dashed lines. Residues from the other monomer are colored in grey and flagged by a prime.

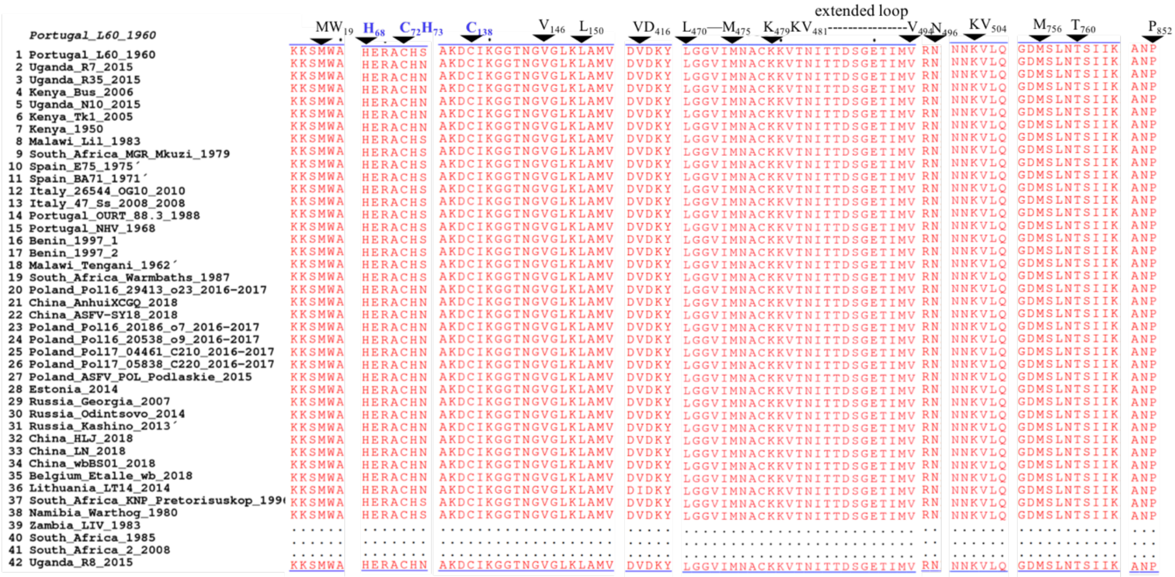

Supplementary Fig. 11. The conservation of the essential residues involved in regulation and drug binding of *Asfv*Top2 in Asfv orthologues. The 42 *Asfv*Top2 protein sequences obtained from the ASFVdb[34] were aligned using Clustal Omega[32]. The figure was the output from ESPript3[33] with default setting. The *Asfv*Top2 sequence in this study is originated from the Portugal_L60_1960 Asfv variant. The labelled residues are involved in the regulation (colored in blue) and drug binding.

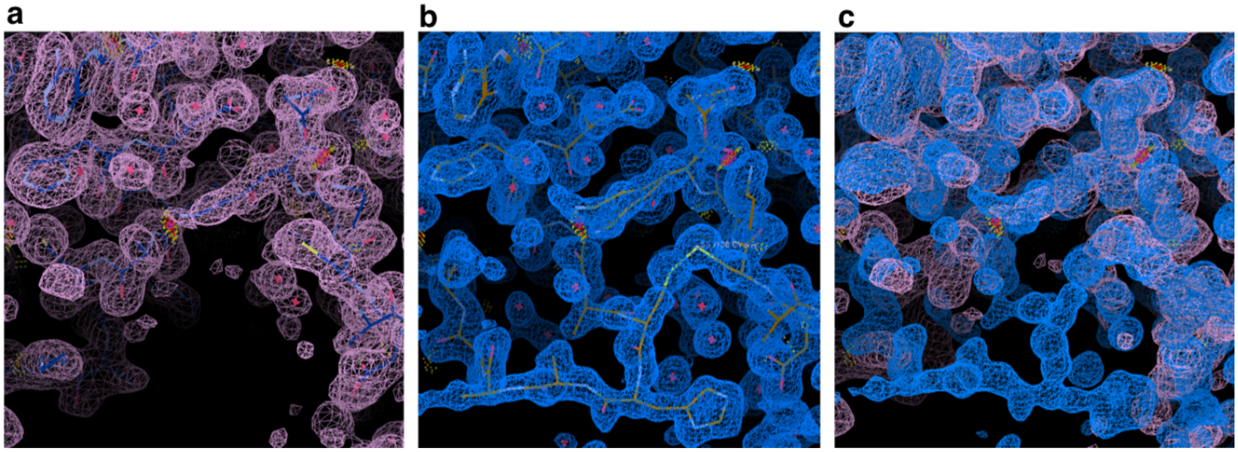

Supplementary Fig. 12. Electron density maps of the ATPase domain complexed with MgAMP-PNP. a. The reduced form (pink, contoured at 0.35σ, *Coot* v.0.8.8) with the modeled structure in blue sticks. **b.** The oxidized form (blue, contoured at 0.35σ, *Coot* v.0.8.8) with the modeled structure in orange sticks. **c.** Superposition of the maps of both reduced and oxidized forms.

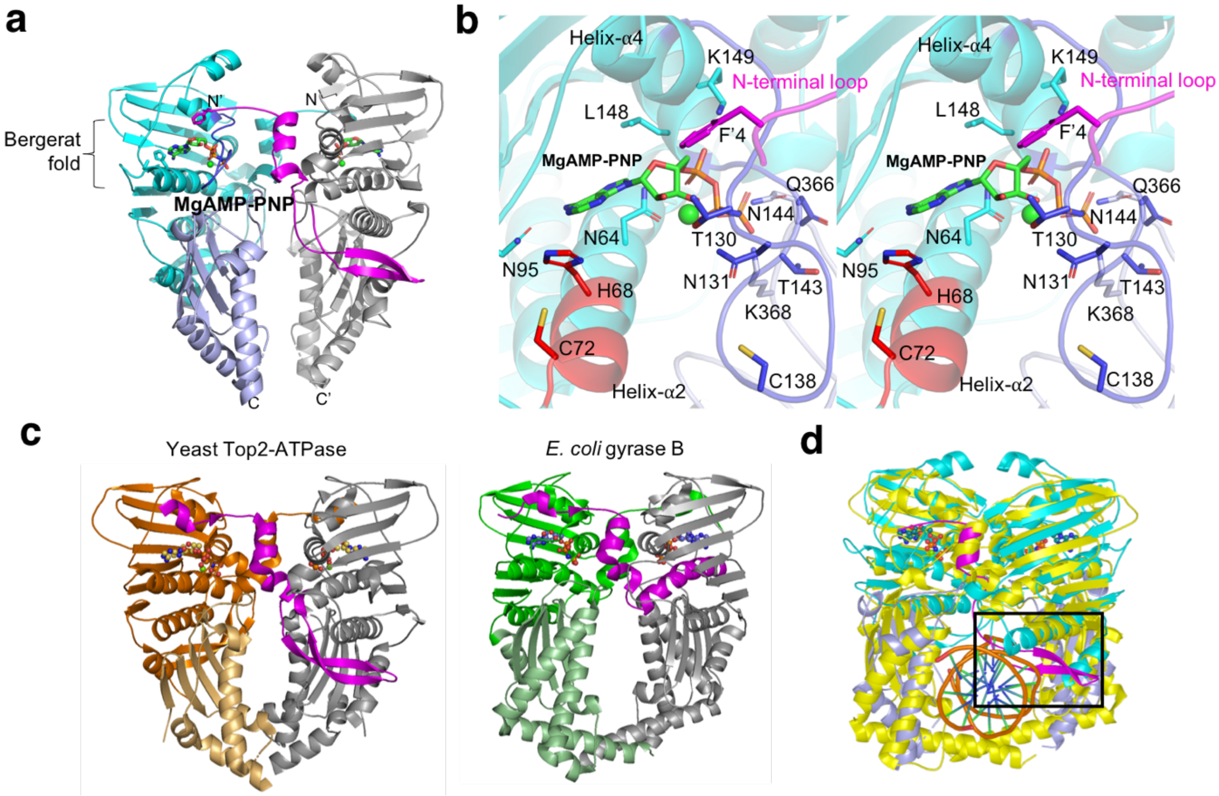

Supplementary Fig. 13. X-ray structure of *Asfv*Top2 ATPase domain complexed with Mg AMP-PNP. a. A dimeric assembly of the domain is presented in cartoon illustration. The ATP-binding and transducer subdomains of one subunit are colored in cyan and light blue, respectively. The former involves the conserved Bergerat fold for constituting the ATP binding site[29]. The bound AMP-PNP is shown as sticks while the Mg^{2+} ion is shown as green sphere. The N-terminal loop (residues A_3-F_9) and the long extended loop (residues F_{125}-G_{147}) adjacent to the active site are respectively colored in magenta and blue. **b.** The stereo-view of the ATP binding site shows the labelled residues contacting with MgAMP-PNP. **c.** The ATPase domain structure of yeast[6] (PDB: 1PVG) comprises the N-terminal loop and ß-hairpin loop insertion (residues L_{31}-I_{46}) (both colored in magenta), whereas the latter is absent in *E. coli*[35] protein (PDB: 1EI1). **d.** Superimposition between the structures of the *Asfv*Top2 ATPase domain and the ParE domain (colored in yellow) of *S. pneumoniae* Top IV complexed with a T-DNA segment (PDB: 5J5Q)[30] indicates the potential DNA contact surface in *Asfv*Top2, and the clash (highlighted in black rectangular) between DNA and the ß-hairpin loop (colored in magenta).

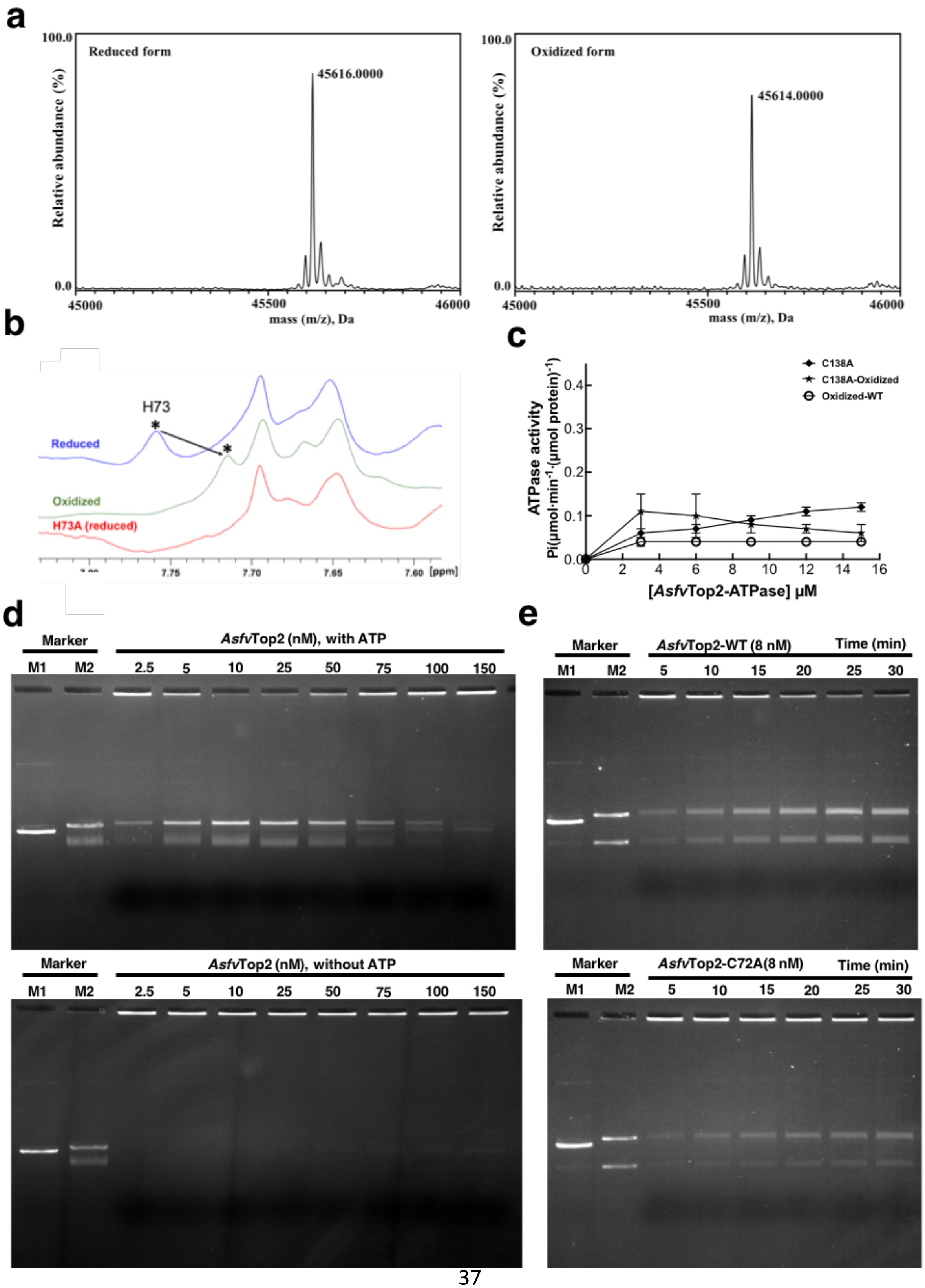
a
Reduced form
45616.0000
Relative abundance (%)
mass (m/z), Da
Oxidized form
45614.0000
b
H73
Reduced
Oxidized
H73A (reduced)
[ppm]
c
C138A
C138A-Oxidized
Oxidized-WT
ATPase activity
Pi(μmol·min⁻¹·(μmol protein)⁻¹)
[AsfvTop2-ATPase] μM
d
Marker
AsfvTop2 (nM), with ATP
M1
M2
2.5
5
10
25
50
75
100
150
AsfvTop2 (nM), without ATP
e
AsfvTop2-WT (8 nM)
Time (min)
AsfvTop2-C72A(8 nM)

Supplementary Fig. 14. Biophysical and biochemical analyses of the *Asfv*Top2 ATPase domain in reduced/oxidized forms and DNA decatenation assays of intact *Asfv*Top2. a. Intact protein mass spectrometry (MS) showing a 2-Da molecular weight difference between the reduced and oxidized forms, supporting the disulfide bond formation in the oxidized form. **b.** 1D proton NMR analyses showing perturbation of the proton signal of H73 upon oxidation. The identity of the H73 signal at 7.758 ppm in the reduced form was confirmed by the absence of this signal in the H73A mutant (spectrum in red). This proton signal was assigned by the characteristic chemical shift-of the imidazole side chain $C^{\varepsilon 1}$-H[36]. **c.** The ATPase activity of oxidized C138A is comparable to that of the reduced C138A. Each data point in 379 panel c includes the mean ± SE value from three independent reactions (n=3) using the same batch of sample. **d.** DNA decatenation by *Asfv*Top2 at serial concentrations (2.5 nM - 150 nM) with presence and absence of ATP shown in upper and lower panels, respectively. Note that DNA catenation occurred at higher concentrations (≥ 50 nM) of *Asfv*Top2 in the presence of ATP at the upper panel. An analogous phenomenon was reported previously[37]. **e.** Time-dependent DNA decatenation by *Asfv*Top2 and C72A mutant are shown in the upper and lower panels, respectively. M1 and M2 are the linear *k*DNA and decatenated *k*DNA markers (TopoGEN, Inc). The raw data of the MS, NMR, and DNA decatenation analyses are provided in the **Supplementary Data 3**, **4**, and **6**, respectively.

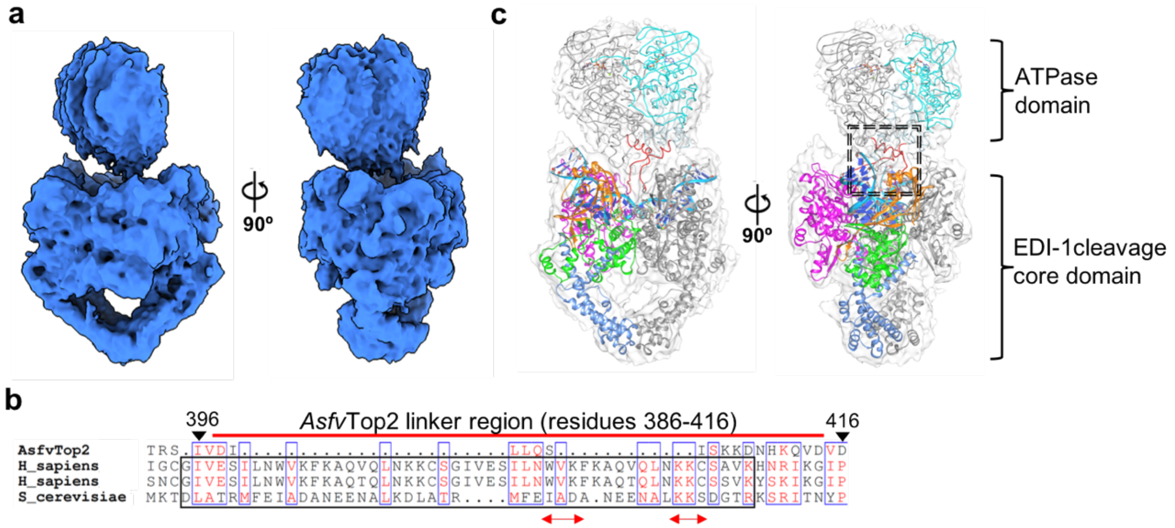

Supplementary Fig. 15. Cryo-EM map of a full-length *Asfv*Top2 DNA/inhibitor complex, and the non-conserved linker region. a. The cryo-EM map (3.68 Å, shown in blue surface) for the full complex of EDI-1, including the ATPase domain and the linker region, as described in **Supplementary Note 4** and **Supplementary Fig. 2m-h**. **b.** Sequence alignment of the linker region in human, yeast and Asfv Top2 proteins. The red arrows highlight the location of the two conserved motifs characterized in eukaryotic Top2 linker[21]. **c.** The atomic model of the full-length *Asfv*Top2. The crystal structure of the *Asfv*Top2 ATPase domain/ MgAMP-PNP bound and cryo-EM structure of the EDI-1 (cleavage core/DNA binding domain) complex were fitted to the cryo-EM densities. The linker region (colored in red) was mostly modeled as a loop conformation.

Supplementary Tables

Supplementary Table 1. The 39 double stranded DNA sequences that contain *Asfv*Top2 cleavage sites. All sequences are listed starting from 5’ end. The Cut02 highlighted in red had the largest number of clones (shown in parenthesis) compared with the others.

	-19	-18	-17	-16	-15	-14	-13	-12	-11	-10	-9	-8	-7	-6	-5	-4	-3	-2	-1	+1	+2	+3	+4	+5	+6	+7	+8	+9	+10	+11	+12	+13	+14	+15	+16	+17	+18	+19	+20	+21
Cut01 (4)	g	a	c	t	t	a	t	c	g	c	c	a	c	t	g	g	c	a	g	c	a	g	c	c	a	c	t	g	g	t	a	a	c	a	g	g	a	t	t	a
Cut01R	g	c	t	a	a	t	c	c	t	g	t	t	a	c	c	a	g	t	g	g	c	t	g	c	t	g	c	c	a	g	t	g	g	c	g	a	t	a	a	g
Cut02 (29)	c	a	g	a	g	c	g	a	g	g	t	a	t	g	t	a	g	g	c	g	g	t	g	c	t	a	c	a	g	a	g	t	t	c	t	t	g	a	a	g
Cut02R	c	a	c	t	t	c	a	a	g	a	a	c	t	c	t	g	t	a	g	c	a	c	c	g	c	c	t	a	c	a	t	a	c	c	t	c	g	c	t	c
Cut03 (2)	t	g	c	g	c	c	t	t	a	t	c	c	g	g	t	a	a	c	t	a	t	c	g	t	c	t	t	g	a	g	t	c	c	a	a	c	c	c	g	g
Cut03R	t	a	c	c	g	g	g	t	t	g	g	a	c	t	c	a	a	g	a	c	g	a	t	a	g	t	t	a	c	c	g	g	a	t	a	a	g	g	c	g
Cut04 (5)	t	g	c	t	a	c	a	g	a	g	t	t	c	t	t	g	a	a	g	t	g	g	t	g	g	c	c	t	a	a	c	t	a	c	g	g	c	t	a	c
Cut04R	g	t	g	t	a	g	c	c	g	t	a	g	t	t	a	g	g	c	c	a	c	c	a	c	t	t	c	a	a	g	a	a	c	t	c	t	g	t	a	g
Cut05 (1)	a	c	a	g	g	a	t	t	a	g	c	a	g	a	g	c	g	a	g	g	t	a	t	g	t	a	g	g	c	g	g	t	g	c	t	a	c	a	g	a
Cut05R	a	c	t	c	t	g	t	a	g	c	a	c	c	g	c	c	t	a	c	a	t	a	c	c	t	c	g	c	t	c	t	g	c	t	a	a	t	c	c	t
Cut06 (1)	a	g	c	a	g	a	g	c	g	a	g	g	t	a	t	g	t	a	g	g	c	g	g	t	g	c	t	a	c	a	g	a	g	t	t	c	t	t	g	a
Cut06R	c	t	t	c	a	a	g	a	a	c	t	c	t	g	t	a	g	c	a	c	c	g	c	c	t	a	c	a	t	a	c	c	t	c	g	c	t	c	t	g
Cut07 (2)	g	t	a	t	g	t	a	g	g	c	g	g	t	g	c	t	a	c	a	g	a	g	t	t	c	t	t	g	a	a	g	t	g	g	t	g	g	c	c	t
Cut07R	t	t	a	g	g	c	c	a	c	c	a	c	t	t	c	a	a	g	a	a	c	t	c	t	g	t	a	g	c	a	c	c	g	c	c	t	a	c	a	t
Cut08 (1)	a	g	g	c	g	g	t	g	c	t	a	c	a	g	a	g	t	t	c	t	t	g	a	a	g	t	g	g	t	g	g	c	c	t	a	a	c	t	a	c
Cut08R	c	c	g	t	a	g	t	t	a	g	g	c	c	a	c	c	a	c	t	t	c	a	a	g	a	a	c	t	c	t	g	t	a	g	c	a	c	c	g	c
Cut09 (7)	t	t	c	t	c	a	t	a	g	c	t	c	a	c	g	c	t	g	t	a	g	g	t	a	t	c	t	c	a	g	t	t	c	g	g	t	g	t	a	g
Cut09R	a	c	c	t	a	c	a	c	c	g	a	a	c	t	g	a	g	a	t	a	c	c	t	a	c	a	g	c	g	t	g	a	g	c	t	a	t	g	a	g
Cut10 (1)	t	t	t	c	t	c	a	t	a	g	c	t	c	a	c	g	c	t	g	t	a	g	g	t	a	t	c	t	c	a	g	t	t	c	g	g	t	g	t	a
Cut10R	c	c	t	a	c	a	c	c	g	a	a	c	t	g	a	g	a	t	a	c	c	t	a	c	a	g	c	g	t	g	a	g	c	t	a	t	g	a	g	a
Cut11 (3)	t	g	g	g	c	t	g	t	g	t	g	c	a	c	g	a	a	c	c	c	c	c	c	g	t	t	c	a	g	c	c	c	g	a	c	c	g	c	t	g
Cut11R	c	g	c	a	g	c	g	g	t	c	g	g	g	c	t	g	a	a	c	g	g	g	g	g	g	t	t	c	g	t	g	c	a	c	a	c	a	g	c	c
Cut12 (3)	t	g	g	t	a	t	c	t	g	c	g	c	t	c	t	g	c	t	g	a	a	g	c	c	a	g	t	t	a	c	c	t	t	c	g	g	a	a	a	a
Cut12R	c	t	t	t	t	t	c	c	g	a	a	g	g	t	a	a	c	t	g	g	c	t	t	c	a	g	c	a	g	a	g	c	g	c	a	g	a	t	a	c
Cut13 (2)	t	a	t	c	t	g	c	g	c	t	c	t	g	c	t	g	a	a	g	c	c	a	g	t	t	a	c	c	t	t	c	g	g	a	a	a	a	a	g	a
Cut13R	a	c	t	c	t	t	t	t	t	c	c	g	a	a	g	g	t	a	a	c	t	g	g	c	t	t	c	a	g	c	a	g	a	g	c	g	c	a	g	a
Cut14 (4)	t	c	t	g	c	g	c	t	c	t	g	c	t	g	a	a	g	c	c	a	g	t	t	a	c	c	t	t	c	g	g	a	a	a	a	a	g	a	g	t
Cut14R	c	a	a	c	t	c	t	t	t	t	t	c	c	g	a	a	g	g	t	a	a	c	t	g	g	c	t	t	c	a	g	c	a	g	a	g	c	g	c	a
Cut15 (6)	a	a	c	t	a	c	g	g	c	t	a	c	a	c	t	a	g	a	a	g	a	a	c	a	g	t	a	t	t	t	g	g	t	a	t	c	t	g	c	g
Cut15R	a	g	c	g	c	a	g	a	t	a	c	c	a	a	a	t	a	c	t	g	t	t	c	t	t	c	t	a	g	t	g	t	a	g	c	c	g	t	a	g
Cut16 (8)	a	c	t	a	c	g	g	c	t	a	c	a	c	t	a	g	a	a	g	a	a	c	a	g	t	a	t	t	t	g	g	t	a	t	c	t	g	c	g	c
Cut16R	g	a	g	c	g	c	a	g	a	t	a	c	c	a	a	a	t	a	c	t	g	t	t	c	t	t	c	t	a	g	t	g	t	a	g	c	c	g	t	a
Cut17 (5)	a	c	c	t	t	c	g	g	a	a	a	a	a	g	a	g	t	t	g	g	t	a	g	c	t	c	t	t	g	a	t	c	c	g	g	c	a	a	a	c
Cut17R	t	t	g	t	t	t	g	c	c	g	g	a	t	c	a	a	g	a	g	c	t	a	c	c	a	a	c	t	c	t	t	t	t	t	c	c	g	a	a	g
Cut18 (2)	t	t	a	t	c	g	c	c	a	c	t	g	g	c	a	g	c	a	g	c	c	a	c	t	g	g	t	a	a	c	a	g	g	a	t	t	a	g	c	a
Cut18R	t	c	t	g	c	t	a	a	t	c	c	t	g	t	t	a	c	c	a	g	t	g	g	c	t	g	c	t	g	c	c	a	g	t	g	g	c	g	a	t
Cut19 (1)	c	c	c	c	c	g	t	t	c	a	g	c	c	c	g	a	c	c	g	c	t	g	c	g	c	c	t	t	a	t	c	c	g	g	t	a	a	c	t	a
Cut19R	g	a	t	a	g	t	t	a	c	c	g	g	a	t	a	a	g	g	c	g	c	a	g	c	g	g	t	c	g	g	g	c	t	g	a	a	c	g	g	g
Cut20 (6)	g	c	t	g	c	g	c	c	t	t	a	t	c	c	g	g	t	a	a	c	t	a	t	c	g	t	c	t	t	g	a	g	t	c	c	a	a	c	c	c
Cut20R	c	c	g	g	g	t	t	g	g	a	c	t	c	a	a	g	a	c	g	a	t	a	g	t	t	a	c	c	g	g	a	t	a	a	g	g	c	g	c	a
Cut21 (1)	c	c	g	a	c	c	g	c	t	g	c	g	c	c	t	t	a	t	c	c	g	g	t	a	a	c	t	a	t	c	g	t	c	t	t	g	a	g	t	c
Cut21R	t	g	g	a	c	t	c	a	a	g	a	c	g	a	t	a	g	t	t	a	c	c	g	g	a	t	a	a	g	g	c	g	c	a	g	c	g	g	t	c
Cut22 (1)	c	g	c	c	t	g	c	g	c	c	t	t	a	t	c	c	g	g	t	a	a	c	t	a	t	c	g	t	c	t	t	g	a	g	t	c	c	a	a	c
Cut22R	g	g	g	t	t	g	g	a	c	t	c	a	a	g	a	c	g	a	t	a	g	t	t	a	c	c	g	g	a	t	a	a	g	g	c	g	c	a	g	c
Cut23 (2)	g	c	t	g	g	g	c	t	g	t	g	t	g	c	a	c	g	a	a	c	c	c	c	c	c	g	t	t	c	a	g	c	c	c	g	a	c	c	g	c
Cut23R	c	a	g	c	g	g	t	c	g	g	g	c	t	g	a	a	c	g	g	g	g	g	g	t	t	c	g	t	g	c	a	c	a	c	a	g	c	c	c	a
Cut24 (1)	c	c	c	c	c	c	g	t	t	c	a	g	c	c	c	g	a	c	c	g	c	t	g	c	g	c	c	t	t	a	t	c	c	g	g	t	a	a	c	t
Cut24R	a	t	a	g	t	t	a	c	c	g	g	a	t	a	a	g	g	c	g	c	a	g	c	g	g	t	c	g	g	g	c	t	g	a	a	c	g	g	g	g
Cut25 (2)	g	c	g	t	g	g	c	g	c	t	t	t	c	t	c	a	t	a	g	c	t	c	a	c	g	c	t	g	t	a	g	g	t	a	t	c	t	c	a	g
Cut25R	a	a	c	t	g	a	g	a	t	a	c	c	t	a	c	a	g	c	g	t	g	a	g	c	t	a	t	g	a	g	a	a	a	g	c	g	c	c	a	c
Cut26 (1)	c	g	t	c	t	t	g	a	g	t	c	c	a	a	c	c	c	g	g	t	a	a	g	a	c	a	c	g	a	c	t	t	a	t	c	g	c	c	a	c
Cut26R	c	a	g	t	g	g	c	g	a	t	a	a	g	t	c	g	t	g	t	c	t	t	a	c	c	g	g	g	t	t	g	g	a	c	t	c	a	a	g	a
Cut27 (2)	t	a	t	c	g	t	c	t	t	g	a	g	t	c	c	a	a	c	c	c	g	g	t	a	a	g	a	c	a	c	g	a	c	t	t	a	t	c	g	c
Cut27R	t	g	g	c	g	a	t	a	a	g	t	c	g	t	g	t	c	t	t	a	c	c	g	g	g	t	t	g	g	a	c	t	c	a	a	g	a	c	g	a
Cut28 (3)	t	a	t	g	t	a	g	g	c	g	g	t	g	c	t	a	c	a	g	a	g	t	t	c	t	t	g	a	a	g	t	g	g	t	g	g	c	c	t	a
Cut28R	g	t	t	a	g	g	c	c	a	c	c	a	c	t	t	c	a	a	g	a	a	c	t	c	t	g	t	a	g	c	a	c	c	g	c	c	t	a	c	a
Cut29 (1)	g	g	t	a	g	c	t	c	t	t	g	a	t	c	c	g	g	c	a	a	a	c	a	a	a	c	c	a	c	c	g	c	t	g	g	t	a	g	c	g
Cut29R	a	c	c	g	c	t	a	c	c	a	g	c	g	g	t	g	g	t	t	t	g	t	t	t	g	c	c	g	g	a	t	c	a	a	g	a	g	c	t	a
Cut30 (1)	c	g	g	c	a	a	a	c	a	a	a	c	c	a	c	c	g	c	t	g	g	t	a	g	c	g	g	t	g	g	t	t	t	t	t	t	t	g	t	t
Cut30R	c	a	a	a	c	a	a	a	a	a	a	a	c	c	a	c	c	g	c	t	a	c	c	a	g	c	g	g	t	g	g	t	t	t	g	t	t	t	g	c
Cut31 (1)	c	a	t	a	g	c	t	c	a	c	g	c	t	g	t	a	g	g	t	a	t	c	t	c	a	g	t	t	c	g	g	t	g	t	a	g	g	t	c	g
Cut31R	a	a	c	g	a	c	c	t	a	c	a	c	c	g	a	a	c	t	g	a	g	a	t	a	c	c	t	a	c	a	g	c	g	t	g	a	g	c	t	a
Cut32 (1)	g	g	t	c	g	t	t	c	g	c	t	c	c	a	a	g	c	t	g	g	g	c	t	g	t	g	t	g	c	a	c	g	a	a	c	c	c	c	c	c
Cut32R	a	c	g	g	g	g	g	g	t	t	c	g	t	g	c	a	c	a	c	a	g	c	c	c	a	g	c	t	t	g	g	a	g	c	g	a	a	c	g	a
Cut33 (1)	g	a	a	c	a	g	t	a	t	t	t	g	g	t	a	t	c	t	g	c	g	c	t	c	t	g	c	t	g	a	a	g	c	c	a	g	t	t	a	c
Cut33R	a	g	g	t	a	a	c	t	g	g	c	t	t	c	a	g	c	a	g	a	g	c	g	c	a	g	a	t	a	c	c	a	a	a	t	a	c	t	g	t
Cut34 (1)	c	a	a	g	c	t	g	g	g	c	t	g	t	g	t	g	c	a	c	g	a	a	c	c	c	c	c	c	g	t	t	c	a	g	c	c	c	g	a	c
Cut34R	c	g	g	t	c	g	g	g	c	t	g	a	a	c	g	g	g	g	g	g	t	t	c	g	t	g	c	a	c	a	c	a	g	c	c	c	a	g	c	t
Cut35 (1)	a	c	a	c	g	a	c	t	t	a	t	c	g	c	c	a	c	t	g	g	c	a	g	c	a	g	c	c	a	c	t	g	g	t	a	a	c	a	g	g
Cut35R	a	t	c	c	t	g	t	t	a	c	c	a	g	t	g	g	c	t	g	c	t	g	c	c	a	g	t	g	g	c	g	a	t	a	a	g	t	c	g	t
Cut36 (1)	t	a	g	c	a	g	a	g	c	g	a	g	g	t	a	t	g	t	a	g	g	c	g	g	t	g	c	t	a	c	a	g	a	g	t	t	c	t	t	g
Cut36R	t	t	c	a	a	g	a	a	c	t	c	t	g	t	a	g	c	a	c	c	g	c	c	t	a	c	a	t	a	c	c	t	c	g	c	t	c	t	g	c
Cut37 (1)	c	t	c	t	t	g	a	t	c	c	g	g	c	a	a	a	c	a	a	a	c	c	a	c	c	g	c	t	g	g	t	a	g	c	g	g	t	g	g	t
Cut37R	a	a	a	c	c	a	c	c	g	c	t	a	c	c	a	g	c	g	g	t	g	g	t	t	t	g	t	t	t	g	c	c	g	g	a	t	c	a	a	g
Cut38 (1)	g	c	c	t	a	a	c	t	a	c	g	g	c	t	a	c	a	c	t	a	g	a	a	g	a	a	c	a	g	t	a	t	t	t	g	g	t	a	t	c
Cut38R	c	a	g	a	t	a	c	c	a	a	a	t	a	c	t	g	t	t	c	t	t	c	t	a	g	t	g	t	a	g	c	c	g	t	a	g	t	t	a	g
Cut39 (1)	g	c	a	g	c	a	g	c	c	a	c	t	g	g	t	a	a	c	a	g	g	a	t	t	a	g	c	a	g	a	g	c	g	a	g	g	t	a	t	g
Cut39R	t	a	c	a	t	a	c	c	t	c	g	c	t	c	t	g	c	t	a	a	t	c	c	t	g	t	t	a	c	c	a	g	t	g	g	c	t	g	c	t

Supplementary Table 2. A consensus DNA sequence that is preferentially cleaved by *Asfv*Top2.

	-10	-9	-8	-7	-6	-5	-4	-3	-2	-1	+1	+2	+3	+4	+5	+6	+7	+8	+9	+10	+11	+12	+13
G	6	6	3	3	4	5	10	8	3	12	7	7	5	5	6	5	4	2	1	10	9	9	5
G%	25%	25%	13%	13%	17%	21%	42%	33%	13%	50%	29%	29%	21%	21%	25%	21%	17%	8%	4%	42%	38%	38%	21%
A	6	8	7	6	3	10	11	7	12	2	10	7	5	2	4	4	5	1	8	7	6	5	5
A%	25%	33%	29%	25%	13%	42%	46%	29%	50%	8%	42%	29%	21%	8%	17%	17%	21%	4%	33%	29%	25%	21%	21%
T	7	5	5	6	7	8	1	5	4	4	2	5	7	10	2	12	7	11	10	3	6	7	8
T%	29%	21%	21%	25%	29%	33%	4%	21%	17%	17%	8%	21%	29%	42%	8%	50%	29%	46%	42%	13%	25%	29%	33%
C	5	5	9	9	10	1	2	4	5	6	5	5	7	7	12	3	8	10	5	4	3	3	6
C%	21%	21%	38%	38%	42%	4%	8%	17%	21%	25%	21%	21%	29%	29%	50%	13%	33%	42%	21%	17%	13%	13%	25%
χ^2 test (p)	0.898	0.73	0.352	0.405	0.205	0.031	0.003	0.641	0.024	0.044	0.112	0.866	0.866	0.112	0.044	0.024	0.641	0.003	0.031	0.205	0.405	0.352	0.73
consensus	-	-	-	-	-	A noC	G A	-	A	G noA	-	-	-	-	noT C	T	-	T C	noG T	-	-	-	-

The frequency of the base occurrence is shown in percentages (**Supplementary Data 2**). The derived consensus sequence is based upon when the frequency of base occurrence is more than 40 %) or less than 10 % (marked in yellow and blue respectively. The numbering in the top blue row indicates the individual nucleotide position. The Top2 cleavage sites (between -1/+1) of the forward and reverse strands are both indicated by the red triangles. The *p*-value (< 0.05) of the individual base position is highlighted in orange.

Supplementary Table 3. Comparison of the consensus sequences recognized by Top2 from different species without inhibitors[25,38-43].

Base position	-10	-9	-8	-7	-6	-5	-4	-3	-2	-1	+1	+2	+3	+4	+5	+6	+7	+8	+9	+10	+11	+12	+13
**Sources**																							
**ASFV**	-	-	-	-	-	A noC	G A	-	A	G noA	-	-	-	-	noT C	T	-	T C	noG T	-	-	-	-
human IIa						(noA)	(noT)	(A) (noC)	-	(noA) C	-	-	-	-	(noT)	-	noG T	noA C	-				
human IIb						-	-	noC	(G)	-	(T)	-	-	-	(noT)	-	noG T	(C)	-				
mouse							(G)	noG (noC)	noT (noC)	noA (noG)	A	-	-	(T)	noT	(noG) (noA)	(A) (T)	(C)					
mouse	-	-	(T)	-	(A)	-	-	(A)	G noT	noA (T) C	A	-	-	(T)	noT	(noG) (T)	(noG) (A)	-	-	(T)	-	(A)	
chicken					T	C	G A	T	G	T	A	G	C	T	A	C	A	T C	G	A			
Drosophila					G	T	-	A T	A	T C	A	T	T	-	A	T	-	-	G	-	-	-	
Drosophila					A	(T) C	G A	T	A	T C	A	T	A	T	G A	T	A	T C	G (A)	T			
Drosophila					T C	A C	C	-	T	A	C	G C	T C	C	C	G T	T C	T C	T	-	-	C	

Supplementary References

1. Coelho, J., Martins, C., Ferreira, F. & Leitao, A. African swine fever virus ORF P1192R codes for a functional type II DNA topoisomerase. *Virology* **474**, 82-93 (2015).
2. Gietz, R.D. Yeast transformation by the LiAc/SS carrier DNA/PEG method. *Methods Mol Biol* **1205**, 1-12 (2014).
3. Kabsch, W. Xds. *Acta Crystallogr D Biol Crystallogr* **66**, 125-132 (2010).
4. Winn, M.D., *et al.* Overview of the CCP4 suite and current developments. *Acta Crystallogr D Biol Crystallogr* **67**, 235-242 (2011).
5. Long, F., Vagin, A.A., Young, P. & Murshudov, G.N. BALBES: a molecular-replacement pipeline. *Acta Crystallogr D Biol Crystallogr* **64**, 125-132 (2008).
6. Classen, S., Olland, S. & Berger, J.M. Structure of the topoisomerase II ATPase region and its mechanism of inhibition by the chemotherapeutic agent ICRF-187. *Proc Natl Acad Sci U S A* **100**, 10629-10634 (2003).
7. Krissinel, E. & Henrick, K. Inference of macromolecular assemblies from crystalline state. *J Mol Biol* **372**, 774-797 (2007).
8. Afonine, P.V., *et al.* Towards automated crystallographic structure refinement with phenix.refine. *Acta Crystallogr D Biol Crystallogr* **68**, 352-367 (2012).
9. Emsley, P. & Cowtan, K. Coot: model-building tools for molecular graphics. *Acta Crystallogr D Biol Crystallogr* **60**, 2126-2132 (2004).
10. Williams, C.J., *et al.* MolProbity: More and better reference data for improved all-atom structure validation. *Protein Sci* **27**, 293-315 (2018).
11. Schneider, C.A., Rasband, W.S. & Eliceiri, K.W. NIH Image to ImageJ: 25 years of image analysis. *Nat Methods* **9**, 671-675 (2012).
12. Osheroff, N. & Zechiedrich, E.L. Calcium-promoted DNA cleavage by eukaryotic topoisomerase II: trapping the covalent enzyme-DNA complex in an active form. *Biochemistry* **26**, 4303-4309 (1987).
13. Liu, M., *et al.* Improved WATERGATE Pulse Sequences for Solvent Suppression in NMR Spectroscopy. *J. Magn. Reson.* **132**, 125-129 (1998).
14. Carter, S.G. & Karl, D.W. Inorganic phosphate assay with malachite green: an improvement and evaluation. *J Biochem Biophys Methods* **7**, 7-13 (1982).
15. Zheng, S.Q., *et al.* MotionCor2: anisotropic correction of beam-induced motion for improved cryo-electron microscopy. *Nat Methods* **14**, 331-332 (2017).
16. Punjani, A., Rubinstein, J.L., Fleet, D.J. & Brubaker, M.A. cryoSPARC: algorithms for rapid unsupervised cryo-EM structure determination. *Nat Methods* **14**, 290-296 (2017).
17. Punjani, A. & Fleet, D.J. 3D variability analysis: Resolving continuous flexibility and discrete heterogeneity from single particle cryo-EM. *J Struct Biol* **213**, 107702 (2021).
18. Pettersen, E.F., *et al.* UCSF Chimera--a visualization system for exploratory research and analysis. *J Comput Chem* **25**, 1605-1612 (2004).
19. Corbett, K.D., Schoeffler, A.J., Thomsen, N.D. & Berger, J.M. The structural basis for substrate specificity in DNA topoisomerase IV. *J Mol Biol* **351**, 545-561 (2005).
20. Baek, M., *et al.* Accurate prediction of protein structures and interactions using a three-track neural network. *Science* **373**, 871-876 (2021).
21. Vanden Broeck, A., *et al.* Structural basis for allosteric regulation of Human

Topoisomerase IIalpha. *Nat Commun* **12**, 2962 (2021).
22. Afonine, P.V.*, et al.* Real-space refinement in PHENIX for cryo-EM and crystallography. *Acta Crystallogr D Struct Biol* **74**, 531-544 (2018).
23. Gonzalez, A., Talavera, A., Almendral, J.M. & Vinuela, E. Hairpin loop structure of African swine fever virus DNA. *Nucleic Acids Res* **14**, 6835-6844 (1986).
24. Dixon, L.K., Chapman, D.A., Netherton, C.L. & Upton, C. African swine fever virus replication and genomics. *Virus Res* **173**, 3-14 (2013).
25. Liu, L.F., Rowe, T.C., Yang, L., Tewey, K.M. & Chen, G.L. Cleavage of DNA by mammalian DNA topoisomerase II. *J Biol Chem* **258**, 15365-15370 (1983).
26. Morrison, A. & Cozzarelli, N.R. Site-specific cleavage of DNA by E. coli DNA gyrase. *Cell* **17**, 175-184 (1979).
27. Wu, C.C.*, et al.* Structural basis of type II topoisomerase inhibition by the anticancer drug etoposide. *Science* **333**, 459-462 (2011).
28. Wu, C.C., Li, Y.C., Wang, Y.R., Li, T.K. & Chan, N.L. On the structural basis and design guidelines for type II topoisomerase-targeting anticancer drugs. *Nucleic Acids Res* **41**, 10630-10640 (2013).
29. Dutta, R. & Inouye, M. GHKL, an emergent ATPase/kinase superfamily. *Trends Biochem Sci* **25**, 24-28 (2000).
30. Laponogov, I.*, et al.* Trapping of the transport-segment DNA by the ATPase domains of a type II topoisomerase. *Nat Commun* **9**, 2579 (2018).
31. Schmidt, B.H., Osheroff, N. & Berger, J.M. Structure of a topoisomerase II-DNA-nucleotide complex reveals a new control mechanism for ATPase activity. *Nat Struct Mol Biol* **19**, 1147-1154 (2012).
32. McWilliam, H.*, et al.* Analysis Tool Web Services from the EMBL-EBI. *Nucleic Acids Res* **41**, W597-600 (2013).
33. Robert, X. & Gouet, P. Deciphering key features in protein structures with the new ENDscript server. *Nucleic Acids Res* **42**, W320-324 (2014).
34. Zhu, Z. & Meng, G. ASFVdb: an integrative resource for genomic and proteomic analyses of African swine fever virus. *Database (Oxford)* **2020**(2020).
35. Brino, L.*, et al.* Dimerization of Escherichia coli DNA-gyrase B provides a structural mechanism for activating the ATPase catalytic center. *J Biol Chem* **275**, 9468-9475 (2000).
36. Horikawa, Y., Naruse, S., Tanaka, C., Hirakawa, K. & Nishikawa, H. Proton NMR relaxation times in ischemic brain edema. *Stroke* **17**, 1149-1152 (1986).
37. Coelho, J., Ferreira, F., Martins, C. & Leitao, A. Functional characterization and inhibition of the type II DNA topoisomerase coded by African swine fever virus. *Virology* **493**, 209-216 (2016).
38. Lockshon, D. & Morris, D.R. Sites of reaction of Escherichia coli DNA gyrase on pBR322 in vivo as revealed by oxolinic acid-induced plasmid linearization. *J Mol Biol* **181**, 63-74 (1985).
39. Sander, M. & Hsieh, T.S. Drosophila topoisomerase II double-strand DNA cleavage: analysis of DNA sequence homology at the cleavage site. *Nucleic Acids Res* **13**, 1057-1072 (1985).
40. Spitzner, J.R., Chung, I.K., Gootz, T.D., McGuirk, P.R. & Muller, M.T. Analysis of

eukaryotic topoisomerase II cleavage sites in the presence of the quinolone CP-115,953 reveals drug-dependent and -independent recognition elements. *Mol Pharmacol* **48**, 238-249 (1995).

41. Cornarotti, M., *et al.* Drug sensitivity and sequence specificity of human recombinant DNA topoisomerases IIalpha (p170) and IIbeta (p180). *Mol Pharmacol* **50**, 1463-1471 (1996).
42. Capranico, G., Kohn, K.W. & Pommier, Y. Local sequence requirements for DNA cleavage by mammalian topoisomerase II in the presence of doxorubicin. *Nucleic Acids Res* **18**, 6611-6619 (1990).
43. Pommier, Y., Capranico, G., Orr, A. & Kohn, K.W. Local base sequence preferences for DNA cleavage by mammalian topoisomerase II in the presence of amsacrine or teniposide. *Nucleic Acids Res* **19**, 5973-5980 (1991).
