# Supplementary material for: A unified view on enzyme catalysis by cryo-EM study of a DNA topoisomerase
Source: Commun Chem. 2024 Feb 28;7:45. doi: 10.1038/s42004-024-01129-y (PMC10901890; doi:10.1038/s42004-024-01129-y)
Supplement: Supplementary file 3 — Description of Additional Supplementary Files [file 42004_2024_1129_MOESM3_ESM.pdf]

Description of Additional Supplementary Files

File name: Supplementary Data 1

Description: Raw agarose gel images for DNA relaxation assays

File name: Supplementary Data 2

Description: Raw data for DNA cleavage site analyses

File name: Supplementary Data 3

Description: Raw data for mass spectrometry analyses of reduced/oxidized AsfvTop2 ATPase domain.

File name: Supplementary Data 4

Description: Raw data for NMR analyses of reduced/oxidized AsfvTop2 ATPase domain.

File name: Supplementary Data 5

Description: Raw data for ATPase activity assays.

File name: Supplementary Data 6

Description: Raw agarose gel images for DNA decatenation assays.

File name: Supplementary Data 7

Description: PDB validation report of apo-AsfvTop2-Ia conformer

File name: Supplementary Data 8

Description: PDB validation report of apo-AsfvTop2-Ib conformer

File name: Supplementary Data 9

Description: PDB validation report of apo-AsfvTop2-IIa conformer

File name: Supplementary Data 10

Description: PDB validation report of apo-AsfvTop2-IIb conformer

File name: Supplementary Data 11

Description: PDB validation report of apo-AsfvTop2-IIIa conformer

File name: Supplementary Data 12

Description: PDB validation report of apo-AsfvTop2-IIIb conformer.

File name: Supplementary Data 13

Description: PDB validation report of AsfvTop2-EDI-1.

File name: Supplementary Data 14

Description: PDB validation report of AsfvTop2-EDI-2.

File name: Supplementary Data 15

Description: PDB validation report of AsfvTop2-EDI-3.

File name: Supplementary Data 16

Description: PDB validation report of AsfvTop2-EDI-1 full-length reconstruction.

File name: Supplementary Data 17

Description: PDB validation report of AsfvTop2-ATPase domain in reduced state.

File name: Supplementary Data 18

Description: PDB validation report of AsfvTop2-ATPase domain in oxidized state.

File name: Supplementary Movie 1

Description: A movie showing the motion of individual apoAsfvTop2 conformational state. The motion was derived from a serial of components obtained from the 3D variability analyses by CryoSPARC3.2.

File name: Supplementary Movie 2

Description: A movie showing the conformation conversion of six apo-AsfvTop2 conformers (from Ia to IIIb).

File name: Supplementary Movie 3

Description: A movie showing the motion between apoAsfvTop2 conformer IIa and EDI-1 complex, with DNA/etoposide and TOPRIM subdomain omitted for clarity. All movies were made using PyMOL Molecular Graphic System (Version 2.2.2).
