# Supplementary figures and images for: A unified view on enzyme catalysis by cryo-EM study of a DNA topoisomerase
Source: Commun Chem. 2024 Feb 28;7:45. doi: 10.1038/s42004-024-01129-y (PMC10901890; doi:10.1038/s42004-024-01129-y)

## Slide 1
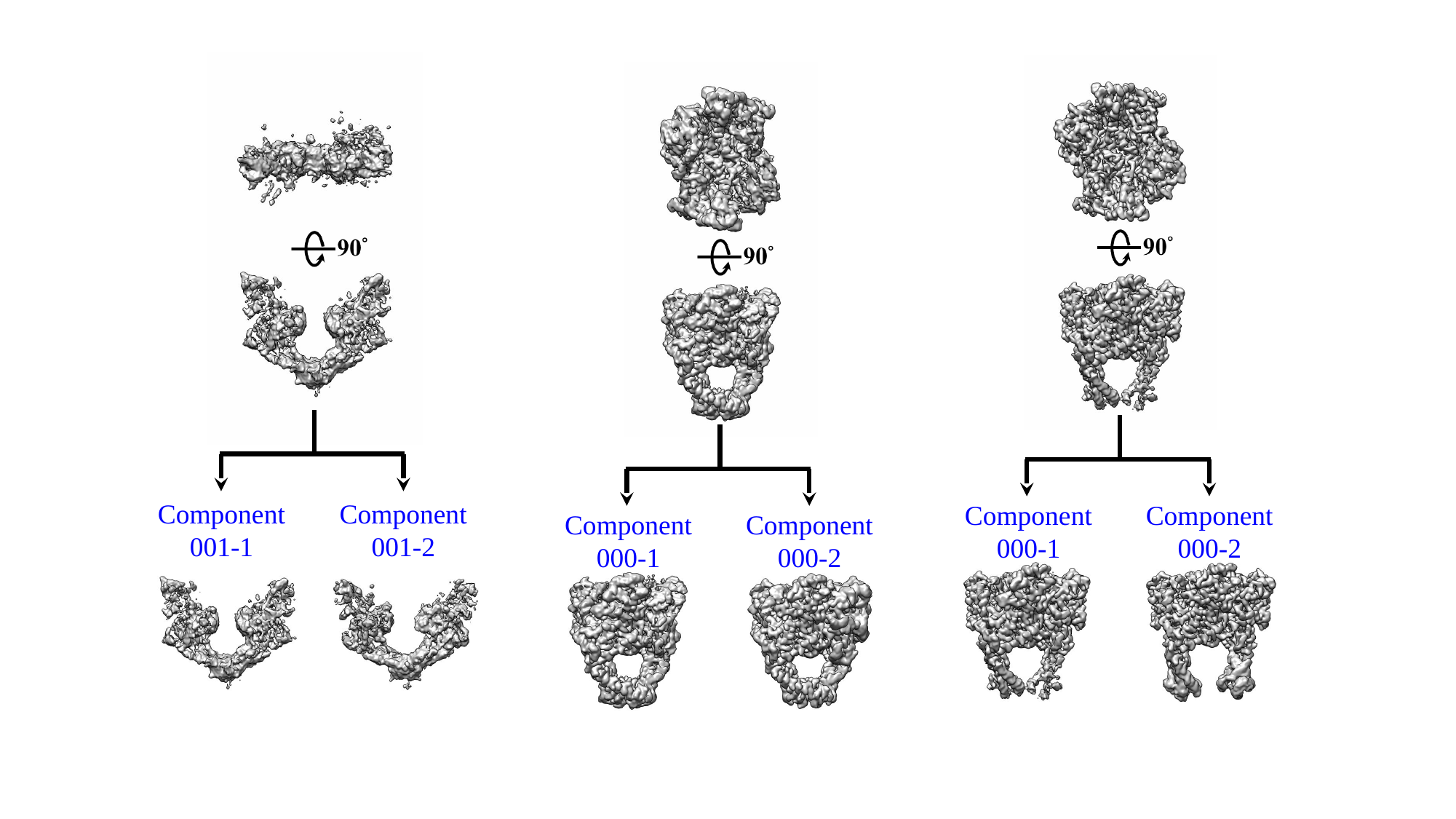

Component
001-1
Component
001-2
Component
000-1
Component
000-2
Component
000-1
Component
000-2

Supplement: Supplementary file 4 — Supplementary Movie 1 [file 42004_2024_1129_MOESM4_ESM.pptx]
