# Supplementary material for: A unified view on enzyme catalysis by cryo-EM study of a DNA topoisomerase
Source: Commun Chem. 2024 Feb 28;7:45. doi: 10.1038/s42004-024-01129-y (PMC10901890; doi:10.1038/s42004-024-01129-y)
Supplement: Supplementary file 16 — Supplementary Data 14 [file 42004_2024_1129_MOESM16_ESM.pdf]

Full wwPDB EM Validation Report (i)

May 18, 2023 – 05:07 PM JST

PDB ID	:	8J9W
EMDB ID	:	EMD-36117
Title	:	Cryo-EM structure of the African swine fever virus topoisomerase 2 complexed with Cut02bDNA and etoposide (EDI-2)
Deposited on	:	2023-05-05
Resolution	:	2.76 Å(reported)
Based on initial model	:	.

This wwPDB validation report is for manuscript review

For Manuscript Review

This is a Full wwPDB EM Validation Report.

This report is produced by the wwPDB biocuration pipeline after annotation of the structure.

We welcome your comments at *validation@mail.wwpdb.org*
A user guide is available at
https://www.wwpdb.org/validation/2017/EMValidationReportHelp
with specific help available everywhere you see the (i) symbol.

The types of validation reports are described at
http://www.wwpdb.org/validation/2017/FAQs#types.

The following versions of software and data (see references (i)) were used in the production of this report:

EMDB validation analysis	:	0.0.1.dev50
Mogul	:	1.8.5 (274361), CSD as541be (2020)
MolProbity	:	4.02b-467
buster-report	:	1.1.7 (2018)
Percentile statistics	:	20191225.v01 (using entries in the PDB archive December 25th 2019)
MapQ	:	1.9.9
Ideal geometry (proteins)	:	Engh & Huber (2001)

1 Overall quality at a glance (i)

The following experimental techniques were used to determine the structure:
ELECTRON MICROSCOPY

The reported resolution of this entry is 2.76 Å.

Percentile scores (ranging between 0-100) for global validation metrics of the entry are shown in the following graphic. The table shows the number of entries on which the scores are based.

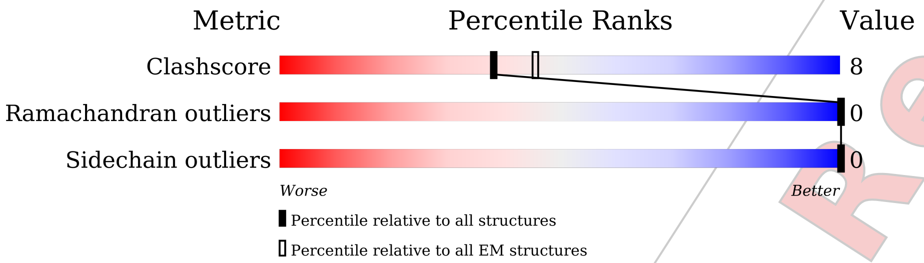

Metric	Whole archive (#Entries)	EM structures (#Entries)
Clashscore	158937	4297
Ramachandran outliers	154571	4023
Sidechain outliers	154315	3826

The table below summarises the geometric issues observed across the polymeric chains and their fit to the map. The red, orange, yellow and green segments of the bar indicate the fraction of residues that contain outliers for >=3, 2, 1 and 0 types of geometric quality criteria respectively. A grey segment represents the fraction of residues that are not modelled. The numeric value for each fraction is indicated below the corresponding segment, with a dot representing fractions <=5% The upper red bar (where present) indicates the fraction of residues that have poor fit to the EM map (all-atom inclusion < 40%). The numeric value is given above the bar.

Mol	Chain	Length	Quality of chain
1	A	1197	• 52% 13% 35%
1	B	1197	• 52% 13% 35%
2	C	13	77% 23%
2	E	13	69% 31%
3	D	17	76% 24%
3	F	17	76% 24%

Ideal geometry (DNA, RNA) : Parkinson et al. (1996)
Validation Pipeline (wwPDB-VP) : 2.32.2

For Manuscript Review

2 Entry composition (i)

There are 5 unique types of molecules in this entry. The entry contains 13841 atoms, of which 0 are hydrogens and 0 are deuteriums.

In the tables below, the AltConf column contains the number of residues with at least one atom in alternate conformation and the Trace column contains the number of residues modelled with at most 2 atoms.

- Molecule 1 is a protein called DNA topoisomerase 2.

Mol	Chain	Residues	Atoms					AltConf	Trace
1	A	775	Total 6283	C 4031	N 1080	O 1144	S 28	0	0
1	B	775	Total 6283	C 4031	N 1080	O 1144	S 28	0	0

There are 10 discrepancies between the modelled and reference sequences:

Chain	Residue	Modelled	Actual	Comment	Reference
A	1193	HIS	-	expression tag	UNP A0A0A1E3Q0
A	1194	HIS	-	expression tag	UNP A0A0A1E3Q0
A	1195	HIS	-	expression tag	UNP A0A0A1E3Q0
A	1196	HIS	-	expression tag	UNP A0A0A1E3Q0
A	1197	HIS	-	expression tag	UNP A0A0A1E3Q0
B	1193	HIS	-	expression tag	UNP A0A0A1E3Q0
B	1194	HIS	-	expression tag	UNP A0A0A1E3Q0
B	1195	HIS	-	expression tag	UNP A0A0A1E3Q0
B	1196	HIS	-	expression tag	UNP A0A0A1E3Q0
B	1197	HIS	-	expression tag	UNP A0A0A1E3Q0

- Molecule 2 is a DNA chain called DNA (5'-D(*AP*AP*GP*AP*AP*CP*TP*CP*TP*GP*TP*AP*G)-3').

Mol	Chain	Residues	Atoms					AltConf	Trace
2	E	13	Total 255	C 123	N 47	O 73	P 12	0	0
2	C	13	Total 266	C 128	N 52	O 74	P 12	0	0

- Molecule 3 is a DNA chain called DNA (5'-D(*CP*AP*TP*GP*CP*TP*AP*CP*AP*GP*AP*GP*TP*TP*CP*TP*T)-3').

For Manuscript Review

Mol	Chain	Residues	Atoms					AltConf	Trace
3	D	17	Total 334	C 161	N 57	O 100	P 16	0	0
3	F	17	Total 334	C 161	N 57	O 100	P 16	0	0

- Molecule 4 is MAGNESIUM ION (three-letter code: MG) (formula: Mg) (labeled as "Ligand of Interest" by depositor).

Mol	Chain	Residues	Atoms		AltConf
4	A	1	Total 1	Mg 1	0
4	B	1	Total 1	Mg 1	0

- Molecule 5 is (5S,5aR,8aR,9R)-9-(4-hydroxy-3,5-dimethoxyphenyl)-8-oxo-5,5a,6,8,8a,9-hexa hydrofuro[3',4':6,7]naphtho[2,3-d][1,3]dioxol -5-yl 4,6-O-[(1R)-ethylidene]-beta-D-glucopyr anoside (three-letter code: EVP) (formula: $C_{29}H_{32}O_{13}$) (labeled as "Ligand of Interest" by depositor).

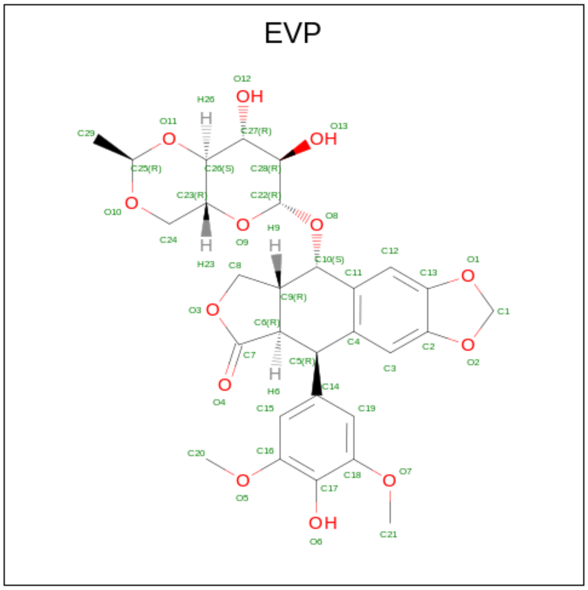

Mol	Chain	Residues	Atoms			AltConf
5	D	1	Total 42	C 29	O 13	0
5	D	1	Total 42	C 29	O 13	0

For Manuscript Review

3 Residue-property plots (i)

These plots are drawn for all protein, RNA, DNA and oligosaccharide chains in the entry. The first graphic for a chain summarises the proportions of the various outlier classes displayed in the second graphic. The second graphic shows the sequence view annotated by issues in geometry and atom inclusion in map density. Residues are color-coded according to the number of geometric quality criteria for which they contain at least one outlier: green = 0, yellow = 1, orange = 2 and red = 3 or more. A red diamond above a residue indicates a poor fit to the EM map for this residue (all-atom inclusion < 40%). Stretches of 2 or more consecutive residues without any outlier are shown as a green connector. Residues present in the sample, but not in the model, are shown in grey.

- Molecule 1: DNA topoisomerase 2

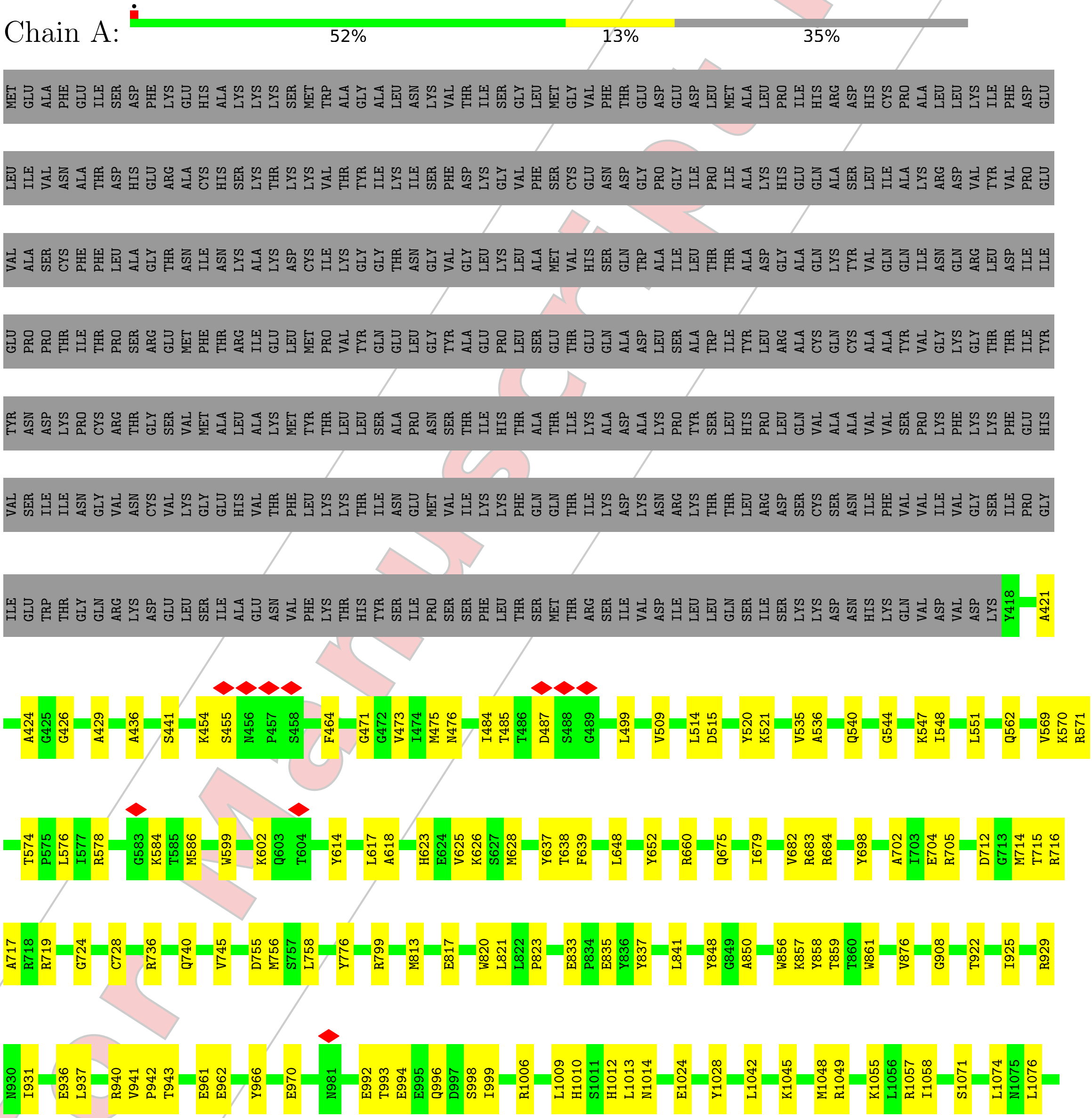

For Manuscript Review

Y1079 G1093 L1097 N1098 I1102 I1103 S1104 P1105 E1106 I1110 L1118 Q1119 G1120 R1131 E1132 I1135 R1141 I1145 M1148 S1162 P1163 F1164 W1170 I1182 K1183 G1184 R1185 Q1188 F1191 H1192 HIS HIS HIS HIS HIS

- Molecule 1: DNA topoisomerase 2

Chain B: 52% 13% 35%

MET GLU ALA PHE GLU ILE SER ASP PHE LYS GLU HIS ALA LYS LYS LYS SER MET TRP ALA GLY ALA LEU ASN LYS VAL THR ILE SER GLY LEU MET GLY VAL PHE THR GLU ASP GLU ASP LEU MET ALA LEU PRO ILE HIS ARG ASP HIS CYS PRO ALA LEU LEU LYS ILE PHE ASP GLU

LEU ILE VAL ASN ALA ALA THR ASP HIS GLU ARG ALA CYS HIS SER LYS THR LYS LYS VAL THR TYR ILE LYS ILE SER PHE ASP LYS GLY VAL PHE SER CYS GLU ASN ASP GLY PRO GLY ILE PRO ILE ALA LYS HIS GLU GLN ALA SER LEU ILE ALA LYS ARG ASP VAL TYR VAL PRO GLU

VAL ALA SER CYS PHE PHE LEU ALA GLY THR ASN ILE ASN LYS ALA LYS ASP CYS ILE LYS GLY GLY THR ASN GLY VAL GLY LEU LYS LEU ALA MET VAL HIS SER GLN TRP ALA ILE LEU THR THR ALA ASP GLY ALA GLN LYS TYR VAL GLN GLN ILE ASN GLN ARG LEU ASP ILE ILE

GLU PRO PRO THR ILE THR PRO SER ARG GLU MET PHE THR ARG ILE GLU LEU MET PRO VAL TYR GLN GLU LEU GLY TYR ALA GLU PRO LEU SER GLU THR GLU GLN ALA ASP LEU SER ALA TRP ILE TYR LEU ARG ALA CYS GLN CYS ALA ALA TYR VAL GLY LYS GLY THR THR ILE TYR

TYR ASN ASP LYS PRO CYS ARG THR GLY SER VAL MET ALA LEU ALA LYS MET TYR THR LEU LEU SER ALA PRO ASN SER THR ILE HIS THR ALA THR ILE LYS ALA ASP ALA LYS PRO TYR SER LEU HIS PRO LEU GLN VAL ALA ALA VAL VAL SER PRO LYS PHE LYS LYS PHE GLU HIS

VAL SER ILE ILE ASN GLY VAL ASN CYS VAL LYS GLY GLU HIS VAL THR PHE LEU LYS LYS THR ILE ASN GLU MET VAL ILE LYS LYS PHE GLN GLN THR ILE LYS ASP LYS ASN ARG LYS THR THR LEU ARG ASP SER CYS SER ASN ILE PHE VAL VAL ILE VAL GLY SER ILE PRO GLY

ILE GLU TRP THR GLY GLN ARG LYS ASP GLU LEU SER ILE ALA GLU ASN VAL PHE LYS THR HIS TYR SER ILE PRO SER SER PHE LEU THR SER MET THR ARG SER ILE VAL ASP ILE LEU LEU GLN SER ILE SER LYS LYS ASP ASN HIS LYS GLN VAL ASP VAL ASP LYS Y418 R422

N423 A436 A437 E438 G439 D440 L443 R447 K454 S455 N456 P457 S458 D463 F464 V473 K480 I484 T485 T486 D487 S488 G489 E490 V509 L514 D515 Y520 Q523 R526 L529 V535 A536 C537 V538 D539 L542 D543 G544 C545 I548 L549

K570 R578 V579 Y580 G583 K584 T585 M586 P587 V588 W599 K602 Q603 T604 V611 G616 L617 E624 F632 Y637 L648 Y652 L664 Q675 I679 R683 Y698 K699 L700 D701 E704 D712 G713 M714 T715 R716 R719 G724 K727

C728 E735 R736 V745 A746 D747 Y751 H752 H753 L758 G771 Y776 I780 G781 I782 R799 S802 V803 Q804 M813 E817 W820 L821 L822 P823 E833 P834 E835 L841 Y848 G849 A850 W856 K857 Y858 T859 T860 W861 L865 I868 V876

T922 I925 R929 N930 I931 I932 T933 E936 L937 P938 L939 R940 V941 P942 T943 V944 A945 Y946 I947 I950 E962 S967 E973 I974 L975 V976 K977 N981 E992 T993 E994 E995 Q996 D997 S998 I999 L1003 H1010 S1011 H1012 L1013 N1014 I1022 L1047 M1048 R1049

K1055 L1056 R1057 I1058 N1069 L1076 Y1079 G1093 N1098 I1102 I1103 S1104 P1105 E1106 I1110 Q1115 L1118 C1121 Y1122 T1123 L1126 I1135 K1138 I1145 M1148 W1170 R1185 K1190 F1191 H1192 HIS HIS HIS HIS HIS

- Molecule 2: DNA (5’-D(*AP*AP*GP*AP*AP*CP*TP*CP*TP*GP*TP*AP*G)-3’)

Chain E: 69% 31%

A1 A2 G3 A4 T7 C8 G13

For Manuscript Review

• Molecule 2: DNA (5'-D(*AP*AP*GP*AP*AP*CP*TP*CP*TP*GP*TP*AP*G)-3')

Chain C:

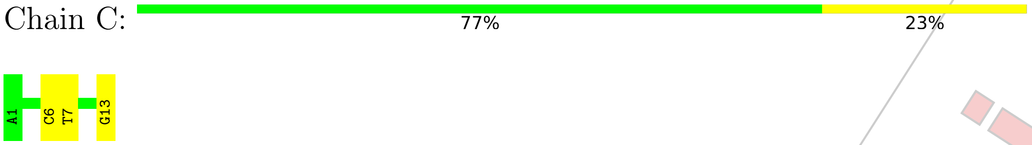

• Molecule 3: DNA (5'-D(*CP*AP*TP*GP*CP*TP*AP*CP*AP*GP*AP*GP*TP*TP*CP*TP *T)-3')

Chain D:

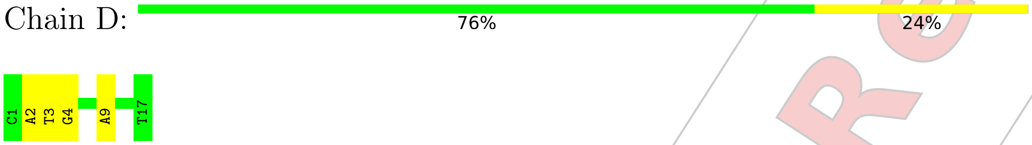

• Molecule 3: DNA (5'-D(*CP*AP*TP*GP*CP*TP*AP*CP*AP*GP*AP*GP*TP*TP*CP*TP *T)-3')

Chain F:

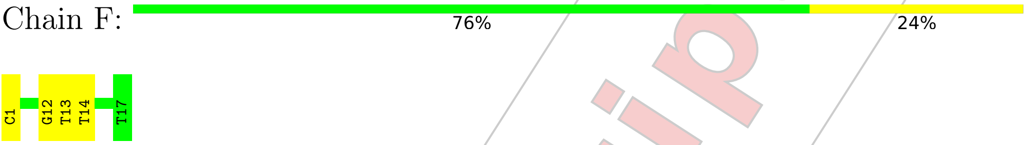

For Manuscript Review

4 Experimental information (i)

Property	Value	Source
EM reconstruction method	SINGLE PARTICLE	Depositor
Imposed symmetry	POINT, Not provided	
Number of particles used	650266	Depositor
Resolution determination method	FSC 0.143 CUT-OFF	Depositor
CTF correction method	PHASE FLIPPING AND AMPLITUDE CORRECTION	Depositor
Microscope	FEI TITAN KRIOS	Depositor
Voltage (kV)	300	Depositor
Electron dose $(e^-/\text{Å}^2)$	42	Depositor
Minimum defocus (nm)	1500	Depositor
Maximum defocus (nm)	2500	Depositor
Magnification	Not provided	
Image detector	GATAN K3 BIOQUANTUM (6k x 4k)	Depositor
Maximum map value	1.468	Depositor
Minimum map value	-0.624	Depositor
Average map value	-0.000	Depositor
Map value standard deviation	0.027	Depositor
Recommended contour level	0.195	Depositor
Map size (Å)	358.56, 358.56, 358.56	wwPDB
Map dimensions	432, 432, 432	wwPDB
Map angles (°)	90.0, 90.0, 90.0	wwPDB
Pixel spacing (Å)	0.83, 0.83, 0.83	Depositor

For Manuscript Review

5 Model quality (i)

5.1 Standard geometry (i)

Bond lengths and bond angles in the following residue types are not validated in this section: MG, EVP

The Z score for a bond length (or angle) is the number of standard deviations the observed value is removed from the expected value. A bond length (or angle) with $|Z| > 5$ is considered an outlier worth inspection. RMSZ is the root-mean-square of all Z scores of the bond lengths (or angles).

Mol	Chain	Bond lengths		Bond angles	
		RMSZ	#$\|Z\|$ >5	RMSZ	#$\|Z\|$ >5
1	A	0.30	0/6430	0.57	0/8688
1	B	0.30	0/6430	0.57	0/8688
2	C	0.57	0/299	0.86	0/460
2	E	0.56	0/286	0.87	0/439
3	D	0.54	0/373	0.96	0/573
3	F	0.59	0/373	0.96	0/573
All	All	0.33	0/14191	0.61	0/19421

There are no bond length outliers.

There are no bond angle outliers.

There are no chirality outliers.

There are no planarity outliers.

5.2 Too-close contacts (i)

In the following table, the Non-H and H(model) columns list the number of non-hydrogen atoms and hydrogen atoms in the chain respectively. The H(added) column lists the number of hydrogen atoms added and optimized by MolProbity. The Clashes column lists the number of clashes within the asymmetric unit, whereas Symm-Clashes lists symmetry-related clashes.

Mol	Chain	Non-H	H(model)	H(added)	Clashes	Symm-Clashes
1	A	6283	0	6290	101	0
1	B	6283	0	6290	107	0
2	C	266	0	148	2	0
2	E	255	0	143	2	0
3	D	334	0	189	4	0
3	F	334	0	189	4	0
4	A	1	0	0	0	0

Continued on next page...

For Manuscript Review

Continued from previous page...

Mol	Chain	Non-H	H(model)	H(added)	Clashes	Symm-Clashes
4	B	1	0	0	0	0
5	D	84	0	64	7	0
All	All	13841	0	13313	215	0

The all-atom clashscore is defined as the number of clashes found per 1000 atoms (including hydrogen atoms). The all-atom clashscore for this structure is 8.

All (215) close contacts within the same asymmetric unit are listed below, sorted by their clash magnitude.

Atom-1	Atom-2	Interatomic distance (Å)	Clash overlap (Å)
1:B:848:TYR:HE2	1:B:940:ARG:CD	1.54	1.19
1:B:848:TYR:CE2	1:B:940:ARG:HD3	1.83	1.14
1:B:542:LEU:HD21	1:B:698:TYR:HA	1.23	1.11
1:B:848:TYR:HE2	1:B:940:ARG:HD3	1.09	1.11
1:B:848:TYR:CE2	1:B:940:ARG:CD	2.46	0.93
1:B:944:VAL:O	1:B:947:ILE:HG13	1.69	0.93
1:B:771:GLY:O	1:B:940:ARG:HD2	1.77	0.85
1:A:1191:PHE:O	1:A:1192:HIS:CD2	2.32	0.82
1:B:542:LEU:CD2	1:B:698:TYR:HA	2.10	0.81
5:D:101:EVP:H15	5:D:101:EVP:H3	1.65	0.78
5:D:101:EVP:C7	5:D:101:EVP:H19	2.16	0.75
1:A:756:MET:SD	3:F:1:DC:N4	2.60	0.75
5:D:101:EVP:H15	5:D:101:EVP:C3	2.19	0.71
1:B:848:TYR:HE2	1:B:940:ARG:NE	1.89	0.70
1:B:865:LEU:HD12	1:B:868:ILE:HD11	1.74	0.68
1:B:771:GLY:O	1:B:940:ARG:CD	2.43	0.66
1:A:1058:ILE:HD11	1:A:1145:ILE:HG23	1.77	0.66
1:B:938:PRO:HG2	1:B:941:VAL:HG21	1.78	0.66
1:B:1058:ILE:HD11	1:B:1145:ILE:HG23	1.78	0.65
1:A:755:ASP:OD2	1:B:799:ARG:NH1	2.31	0.64
1:B:545:CYS:HA	1:B:549:LEU:HD12	1.81	0.63
1:B:776:TYR:OH	1:B:1185:ARG:NH2	2.32	0.63
1:A:535:VAL:HG22	1:A:570:LYS:HB2	1.81	0.62
1:A:1049:ARG:HA	1:A:1110:ILE:HD11	1.80	0.62
1:B:942:PRO:HG2	1:B:945:ALA:HB3	1.81	0.62
1:A:637:TYR:HA	1:A:683:ARG:HB3	1.82	0.62
1:B:992:GLU:HG3	1:B:997:ASP:HA	1.81	0.62
1:B:539:ASP:OD1	1:B:617:LEU:HG	2.00	0.62
1:A:993:THR:HG22	1:A:994:GLU:N	2.15	0.62
1:B:848:TYR:CE2	1:B:940:ARG:NE	2.65	0.62
1:A:799:ARG:NH2	3:D:2:DA:OP2	2.32	0.61

Continued on next page...

For Manuscript Review

Continued from previous page...

Atom-1	Atom-2	Interatomic distance (Å)	Clash overlap (Å)
1:A:712:ASP:HB3	1:A:714:MET:HG2	1.82	0.61
1:A:540:GLN:HE22	1:A:571:ARG:HH22	1.48	0.61
1:B:578:ARG:NH1	1:B:624:GLU:OE1	2.33	0.61
1:A:570:LYS:HG2	1:A:638:THR:HA	1.85	0.59
1:B:637:TYR:HA	1:B:683:ARG:HB3	1.84	0.59
1:A:704:GLU:OE1	1:A:716:ARG:NH1	2.36	0.59
1:A:702:ALA:HA	1:A:705:ARG:HG2	1.85	0.58
1:B:535:VAL:HG22	1:B:570:LYS:HB2	1.86	0.57
1:A:993:THR:HG22	1:A:994:GLU:H	1.70	0.57
1:B:712:ASP:HB3	1:B:714:MET:HG2	1.85	0.57
1:B:929:ARG:HG3	1:B:931:ILE:HG12	1.87	0.57
1:A:929:ARG:HG3	1:A:931:ILE:HG12	1.87	0.56
1:A:970:GLU:OE1	1:A:1188:GLN:NE2	2.38	0.56
1:A:574:THR:HG21	1:A:617:LEU:HD21	1.87	0.56
1:A:1191:PHE:O	1:A:1192:HIS:CG	2.58	0.56
1:B:544:GLY:HA2	1:B:548:ILE:HG12	1.87	0.56
1:A:876:VAL:HA	1:A:1164:PHE:HZ	1.70	0.56
1:A:426:GLY:H	1:A:429:ALA:HB2	1.71	0.56
1:B:438:GLU:HB3	1:B:538:VAL:HG12	1.86	0.56
1:A:937:LEU:HD13	1:A:943:THR:HG22	1.87	0.56
1:A:728:CYS:SG	1:A:736:ARG:NH1	2.79	0.55
1:A:441:SER:HB2	1:A:618:ALA:HB2	1.87	0.55
1:B:599:TRP:HA	1:B:602:LYS:HG2	1.87	0.55
1:B:728:CYS:SG	1:B:736:ARG:NH1	2.80	0.55
1:B:947:ILE:HA	1:B:950:ILE:HG22	1.87	0.54
1:A:1045:LYS:HA	1:A:1048:MET:SD	2.48	0.54
1:A:961:GLU:O	1:A:962:GLU:HG3	2.06	0.54
1:B:704:GLU:OE1	1:B:716:ARG:NH1	2.41	0.53
1:B:509:VAL:HG22	1:B:514:LEU:HD11	1.90	0.53
1:A:509:VAL:HG22	1:A:514:LEU:HD11	1.91	0.53
1:A:578:ARG:HB2	1:A:614:TYR:HE2	1.74	0.53
1:A:544:GLY:O	1:A:548:ILE:N	2.41	0.52
1:B:579:VAL:HG22	1:B:611:VAL:HG22	1.92	0.52
1:B:1058:ILE:HD13	1:B:1148:MET:HB2	1.92	0.51
1:B:848:TYR:HE2	1:B:940:ARG:CG	2.21	0.51
1:B:922:THR:HG22	1:B:936:GLU:HB3	1.92	0.51
1:B:925:ILE:HD11	1:B:999:ILE:HG13	1.93	0.51
1:B:719:ARG:HG3	1:B:833:GLU:HG3	1.93	0.51
1:A:599:TRP:HA	1:A:602:LYS:HG2	1.92	0.51
1:A:712:ASP:OD1	1:A:837:TYR:N	2.44	0.51
1:A:776:TYR:OH	1:A:1185:ARG:NH2	2.43	0.51

Continued on next page...

For Manuscript Review

Continued from previous page...

Atom-1	Atom-2	Interatomic distance (Å)	Clash overlap (Å)
1:B:1010:HIS:NE2	3:F:12:DG:OP1	2.43	0.50
1:A:925:ILE:HD11	1:A:999:ILE:HG13	1.93	0.50
1:A:1184:GLY:HA3	1:A:1191:PHE:CZ	2.46	0.50
1:B:857:LYS:O	1:B:1014:ASN:ND2	2.44	0.50
1:B:967:SER:HB3	1:B:973:GLU:H	1.76	0.50
1:B:539:ASP:CG	1:B:616:GLY:HA2	2.31	0.50
1:A:857:LYS:O	1:A:1014:ASN:ND2	2.44	0.50
1:B:724:GLY:HA3	1:B:745:VAL:HG12	1.92	0.50
1:A:1058:ILE:HD13	1:A:1148:MET:HB2	1.93	0.49
1:B:458:SER:HB2	1:B:632:PHE:HB3	1.93	0.49
1:A:473:VAL:HG23	2:C:13:DG:H1’	1.94	0.49
1:A:740:GLN:NE2	1:B:747:ASP:OD2	2.44	0.49
1:A:996:GLN:HB3	1:A:1006:ARG:HD3	1.94	0.49
1:B:848:TYR:CZ	1:B:940:ARG:HD3	2.42	0.49
1:B:542:LEU:HD13	1:B:701:ASP:OD2	2.11	0.49
1:B:580:TYR:OH	1:B:624:GLU:OE1	2.28	0.49
1:A:859:THR:OG1	1:A:1012:HIS:N	2.42	0.49
3:F:13:DT:H2”	3:F:14:DT:H5”	1.95	0.49
1:A:1042:LEU:HA	1:A:1045:LYS:HG2	1.95	0.49
1:B:745:VAL:HG23	1:B:758:LEU:HD21	1.94	0.49
1:B:821:LEU:HD11	1:B:1049:ARG:HD3	1.95	0.49
1:A:922:THR:HG22	1:A:936:GLU:HB3	1.93	0.49
1:B:1104:SER:OG	1:B:1106:GLU:OE2	2.31	0.49
1:A:966:TYR:O	1:B:454:LYS:NZ	2.42	0.49
1:B:484:ILE:HG13	1:B:485:THR:HG22	1.95	0.49
1:A:547:LYS:HD2	1:A:698:TYR:CZ	2.48	0.48
1:A:719:ARG:HG3	1:A:833:GLU:HG3	1.94	0.48
1:A:623:HIS:HA	1:A:626:LYS:HG2	1.94	0.48
1:A:436:ALA:HB3	1:A:536:ALA:HA	1.93	0.48
1:A:848:TYR:HE2	1:A:940:ARG:HD3	1.78	0.48
1:B:664:LEU:HD11	1:B:699:LYS:HB3	1.95	0.48
1:A:584:LYS:HD3	1:A:586:MET:HB2	1.94	0.48
1:A:937:LEU:HD21	1:A:941:VAL:HG13	1.95	0.48
1:A:993:THR:CG2	1:A:994:GLU:H	2.26	0.48
1:A:861:TRP:NE1	1:A:1010:HIS:O	2.42	0.48
1:A:515:ASP:H	1:A:520:TYR:HH	1.59	0.48
1:A:993:THR:CG2	1:A:994:GLU:N	2.77	0.48
1:B:423:ASN:HB3	1:B:464:PHE:HE1	1.79	0.47
1:B:735:GLU:HB2	1:B:804:GLN:HE21	1.78	0.47
5:D:102:EVP:C12	5:D:102:EVP:H22	2.44	0.47
1:A:660:ARG:NH1	3:D:9:DA:OP1	2.47	0.47

Continued on next page...

For Manuscript Review

Continued from previous page...

Atom-1	Atom-2	Interatomic distance (Å)	Clash overlap (Å)
1:A:1104:SER:OG	1:A:1106:GLU:OE2	2.32	0.47
1:B:422:ARG:NH1	1:B:463:ASP:OD1	2.40	0.47
1:B:440:ASP:HA	1:B:443:LEU:HB2	1.97	0.47
1:A:823:PRO:HG2	1:A:835:GLU:HB3	1.97	0.47
1:B:542:LEU:C	1:B:542:LEU:HD23	2.34	0.47
1:B:859:THR:OG1	1:B:1012:HIS:N	2.47	0.47
1:A:724:GLY:HA3	1:A:745:VAL:HG12	1.95	0.47
1:B:962:GLU:HG3	1:B:977:LYS:HB2	1.96	0.47
1:A:856:TRP:NE1	1:A:1024:GLU:OE2	2.48	0.47
1:A:544:GLY:HA2	1:A:548:ILE:HG12	1.96	0.46
1:B:1076:LEU:HG	1:B:1079:TYR:HD2	1.80	0.46
2:C:6:DC:H2'	2:C:7:DT:H71	1.96	0.46
1:B:1049:ARG:HA	1:B:1110:ILE:HD11	1.97	0.46
1:B:823:PRO:HG2	1:B:835:GLU:HB3	1.97	0.46
1:A:1057:ARG:HG3	1:A:1102:ILE:HG22	1.98	0.46
1:A:1076:LEU:HG	1:A:1079:TYR:HD2	1.80	0.46
1:B:1057:ARG:HG3	1:B:1102:ILE:HG22	1.98	0.46
1:B:1121:CYS:HB2	1:B:1123:THR:HG23	1.97	0.46
1:B:861:TRP:NE1	1:B:1010:HIS:O	2.41	0.46
1:A:1013:LEU:HD21	1:A:1028:TYR:HE1	1.81	0.46
1:A:484:ILE:HG13	1:A:485:THR:HG22	1.97	0.46
1:A:745:VAL:HG23	1:A:758:LEU:HD21	1.97	0.46
1:B:946:TYR:OH	1:B:1003:LEU:HB3	2.16	0.46
1:B:993:THR:OG1	1:B:995:GLU:HG3	2.15	0.46
1:A:475:MET:HE3	1:A:499:LEU:HA	1.97	0.45
1:A:813:MET:HE3	1:A:841:LEU:H	1.81	0.45
1:A:540:GLN:HG2	1:A:576:LEU:HB2	1.98	0.45
1:B:933:THR:HG22	1:B:975:LEU:HD22	1.98	0.45
1:B:813:MET:HE3	1:B:841:LEU:H	1.82	0.45
1:A:471:GLY:HA2	5:D:101:EVP:O5	2.17	0.45
1:A:515:ASP:OD1	1:A:515:ASP:N	2.48	0.45
1:A:941:VAL:HA	1:A:942:PRO:HD3	1.79	0.45
5:D:102:EVP:H6	5:D:102:EVP:H28	1.98	0.45
1:A:712:ASP:OD2	1:A:719:ARG:NE	2.48	0.44
1:A:625:VAL:HA	1:A:628:MET:HG2	2.00	0.44
1:A:821:LEU:HD11	1:A:1049:ARG:HD3	2.00	0.44
1:B:584:LYS:HD3	1:B:586:MET:HB2	1.99	0.44
1:A:421:ALA:HB3	1:A:424:ALA:HB2	1.99	0.44
1:B:515:ASP:OD1	1:B:515:ASP:N	2.50	0.44
1:A:1098:ASN:O	1:A:1102:ILE:HG12	2.18	0.43
1:B:850:ALA:HA	1:B:858:TYR:O	2.18	0.43

Continued on next page...

For Manuscript Review

Continued from previous page...

Atom-1	Atom-2	Interatomic distance (Å)	Clash overlap (Å)
1:A:455:SER:O	1:A:455:SER:OG	2.32	0.43
1:A:648:LEU:HD12	1:A:652:TYR:HE2	1.84	0.43
1:B:515:ASP:H	1:B:520:TYR:HH	1.65	0.43
2:E:7:DT:H2'	2:E:8:DC:C6	2.53	0.43
1:B:648:LEU:HD12	1:B:652:TYR:HE1	1.83	0.43
1:A:1055:LYS:HA	1:A:1058:ILE:HG22	2.00	0.43
1:A:861:TRP:CE2	1:A:1009:LEU:HB2	2.53	0.43
1:B:473:VAL:HG22	1:B:548:ILE:HD11	2.00	0.43
1:B:937:LEU:HB2	1:B:938:PRO:HD2	2.00	0.43
1:B:1055:LYS:HA	1:B:1058:ILE:HG22	2.01	0.43
1:B:848:TYR:CE2	1:B:940:ARG:CG	3.01	0.43
1:B:436:ALA:HB3	1:B:536:ALA:HA	2.00	0.43
1:B:523:GLN:OE1	1:B:526:ARG:NH1	2.52	0.42
1:B:727:LYS:HE2	1:B:727:LYS:HB3	1.85	0.42
1:B:1115:GLN:HA	1:B:1118:LEU:HD23	2.01	0.42
1:A:454:LYS:NZ	1:B:973:GLU:OE2	2.33	0.42
1:A:1132:GLU:OE1	1:A:1141:ARG:NH2	2.53	0.42
1:B:780:ILE:HD11	1:B:1190:LYS:HA	2.00	0.42
1:A:487:ASP:OD1	1:A:487:ASP:N	2.53	0.42
1:A:715:THR:HG22	1:A:717:ALA:H	1.84	0.42
1:B:675:GLN:O	1:B:679:ILE:HG12	2.19	0.42
1:A:682:VAL:HG22	1:A:684:ARG:HG2	2.00	0.42
1:B:876:VAL:HG11	1:B:1170:TRP:HB3	2.02	0.42
1:A:1182:ILE:HD13	1:A:1182:ILE:HA	1.95	0.42
1:B:782:ILE:O	1:B:802:SER:HB2	2.20	0.42
1:A:850:ALA:HA	1:A:858:TYR:O	2.19	0.42
1:B:545:CYS:O	1:B:545:CYS:SG	2.78	0.42
1:B:1047:LEU:HD23	1:B:1047:LEU:HA	1.84	0.42
3:D:4:DG:C4	5:D:101:EVP:H12	2.55	0.42
1:A:961:GLU:O	1:A:962:GLU:CG	2.68	0.41
1:B:480:LYS:HA	1:B:480:LYS:HD3	1.87	0.41
1:B:526:ARG:HA	1:B:529:LEU:HD23	2.02	0.41
1:B:542:LEU:HD11	1:B:701:ASP:HB2	2.01	0.41
3:F:12:DG:H2'	3:F:13:DT:C5	2.55	0.41
1:A:1071:SER:HA	1:A:1074:LEU:HB3	2.03	0.41
1:B:817:GLU:HA	1:B:820:TRP:CD1	2.55	0.41
1:B:856:TRP:CE2	1:B:1022:ILE:HG21	2.56	0.41
1:A:547:LYS:O	1:A:551:LEU:HD12	2.20	0.41
1:A:569:VAL:HG12	1:A:639:PHE:HD2	1.86	0.41
1:A:817:GLU:HA	1:A:820:TRP:CD1	2.56	0.41
1:B:487:ASP:OD1	1:B:487:ASP:N	2.53	0.41

Continued on next page...

For Manuscript Review

Continued from previous page...

Atom-1	Atom-2	Interatomic distance (Å)	Clash overlap (Å)
1:A:521:LYS:HG2	1:A:562:GLN:HG3	2.03	0.41
1:A:876:VAL:HG11	1:A:1170:TRP:HB3	2.02	0.41
1:B:1069:ASN:OD1	1:B:1138:LYS:NZ	2.40	0.41
1:A:675:GLN:O	1:A:679:ILE:HG12	2.20	0.41
1:A:908:GLY:N	1:A:936:GLU:HG2	2.36	0.41
1:A:992:GLU:HB3	1:A:998:SER:N	2.36	0.41
1:A:1097:LEU:HA	1:A:1120:GLY:HA3	2.03	0.41
1:B:584:LYS:HD2	1:B:586:MET:H	1.87	0.40
3:D:3:DT:H2"	3:D:4:DG:C8	2.56	0.40
1:A:421:ALA:HB1	1:A:464:PHE:H	1.86	0.40
1:B:1098:ASN:O	1:B:1102:ILE:HG12	2.21	0.40
1:B:1098:ASN:OD1	1:B:1098:ASN:N	2.54	0.40
1:B:443:LEU:O	1:B:447:ARG:HG2	2.21	0.40
1:B:579:VAL:H	1:B:588:VAL:HG22	1.86	0.40
1:B:542:LEU:HD23	1:B:542:LEU:O	2.22	0.40
2:E:3:DG:H2"	2:E:4:DA:C8	2.57	0.40
1:A:476:ASN:HB3	1:A:551:LEU:HD23	2.04	0.40
1:A:1131:ARG:HB3	1:B:1126:LEU:HB3	2.04	0.40
1:B:751:TYR:CZ	1:B:753:HIS:HB2	2.57	0.40
1:B:817:GLU:HA	1:B:820:TRP:HD1	1.86	0.40

There are no symmetry-related clashes.

5.3 Torsion angles (i)

5.3.1 Protein backbone (i)

In the following table, the Percentiles column shows the percent Ramachandran outliers of the chain as a percentile score with respect to all PDB entries followed by that with respect to all EM entries.

The Analysed column shows the number of residues for which the backbone conformation was analysed, and the total number of residues.

Mol	Chain	Analysed	Favoured	Allowed	Outliers	Percentiles	
1	A	773/1197 (65%)	737 (95%)	36 (5%)	0	100	100
1	B	773/1197 (65%)	743 (96%)	30 (4%)	0	100	100
All	All	1546/2394 (65%)	1480 (96%)	66 (4%)	0	100	100

There are no Ramachandran outliers to report.

For Manuscript Review

5.3.2 Protein sidechains (i)

In the following table, the Percentiles column shows the percent sidechain outliers of the chain as a percentile score with respect to all PDB entries followed by that with respect to all EM entries.

The Analysed column shows the number of residues for which the sidechain conformation was analysed, and the total number of residues.

Mol	Chain	Analysed	Rotameric	Outliers	Percentiles	
1	A	680/1048 (65%)	680 (100%)	0	100	100
1	B	680/1048 (65%)	680 (100%)	0	100	100
All	All	1360/2096 (65%)	1360 (100%)	0	100	100

There are no protein residues with a non-rotameric sidechain to report.

Sometimes sidechains can be flipped to improve hydrogen bonding and reduce clashes. All (9) such sidechains are listed below:

Mol	Chain	Res	Type
1	A	540	GLN
1	A	740	GLN
1	A	752	HIS
1	A	1014	ASN
1	A	1192	HIS
1	B	752	HIS
1	B	804	GLN
1	B	1014	ASN
1	B	1186	ASN

5.3.3 RNA (i)

There are no RNA molecules in this entry.

5.4 Non-standard residues in protein, DNA, RNA chains (i)

There are no non-standard protein/DNA/RNA residues in this entry.

5.5 Carbohydrates (i)

There are no monosaccharides in this entry.

For Manuscript Review

5.6 Ligand geometry (i)

Of 4 ligands modelled in this entry, 2 are monoatomic - leaving 2 for Mogul analysis.

In the following table, the Counts columns list the number of bonds (or angles) for which Mogul statistics could be retrieved, the number of bonds (or angles) that are observed in the model and the number of bonds (or angles) that are defined in the Chemical Component Dictionary. The Link column lists molecule types, if any, to which the group is linked. The Z score for a bond length (or angle) is the number of standard deviations the observed value is removed from the expected value. A bond length (or angle) with $|Z| > 2$ is considered an outlier worth inspection. RMSZ is the root-mean-square of all Z scores of the bond lengths (or angles).

Mol	Type	Chain	Res	Link	Bond lengths			Bond angles		
					Counts	RMSZ	$\#|Z| > 2$	Counts	RMSZ	$\#|Z| > 2$
5	EVP	D	102	-	48,48,48	0.60	0	72,73,73	0.90	2 (2%)
5	EVP	D	101	-	48,48,48	0.72	0	72,73,73	1.17	7 (9%)

In the following table, the Chirals column lists the number of chiral outliers, the number of chiral centers analysed, the number of these observed in the model and the number defined in the Chemical Component Dictionary. Similar counts are reported in the Torsion and Rings columns. '-' means no outliers of that kind were identified.

Mol	Type	Chain	Res	Link	Chirals	Torsions	Rings
5	EVP	D	102	-	-	5/12/76/76	0/7/7/7
5	EVP	D	101	-	-	6/12/76/76	1/7/7/7

There are no bond length outliers.

All (9) bond angle outliers are listed below:

Mol	Chain	Res	Type	Atoms	Z	Observed(^{o})	Ideal(^{o})
5	D	102	EVP	C22-O8-C10	4.49	122.30	114.99
5	D	101	EVP	C22-O8-C10	4.38	122.12	114.99
5	D	102	EVP	C11-C4-C5	3.64	124.00	114.40
5	D	101	EVP	C6-C9-C10	-3.63	103.63	110.87
5	D	101	EVP	C11-C4-C5	3.41	123.41	114.40
5	D	101	EVP	C11-C10-C9	-2.71	108.21	111.51
5	D	101	EVP	C22-C28-C27	2.53	115.26	110.00
5	D	101	EVP	O9-C22-C28	2.27	115.16	110.35
5	D	101	EVP	C14-C5-C6	2.15	116.74	113.31

There are no chirality outliers.

All (11) torsion outliers are listed below:

For Manuscript Review

Mol	Chain	Res	Type	Atoms
5	D	102	EVP	C11-C10-O8-C22
5	D	102	EVP	C17-C16-O5-C20
5	D	102	EVP	C17-C18-O7-C21
5	D	101	EVP	C17-C16-O5-C20
5	D	102	EVP	C15-C16-O5-C20
5	D	101	EVP	C19-C18-O7-C21
5	D	101	EVP	C15-C16-O5-C20
5	D	102	EVP	C19-C18-O7-C21
5	D	101	EVP	C17-C18-O7-C21
5	D	101	EVP	C19-C14-C5-C6
5	D	101	EVP	C15-C14-C5-C6

All (1) ring outliers are listed below:

Mol	Chain	Res	Type	Atoms
5	D	101	EVP	C23-C24-C25-C26-O10-O11

2 monomers are involved in 7 short contacts:

Mol	Chain	Res	Type	Clashes	Symm-Clashes
5	D	102	EVP	2	0
5	D	101	EVP	5	0

The following is a two-dimensional graphical depiction of Mogul quality analysis of bond lengths, bond angles, torsion angles, and ring geometry for all instances of the Ligand of Interest. In addition, ligands with molecular weight > 250 and outliers as shown on the validation Tables will also be included. For torsion angles, if less then 5% of the Mogul distribution of torsion angles is within 10 degrees of the torsion angle in question, then that torsion angle is considered an outlier. Any bond that is central to one or more torsion angles identified as an outlier by Mogul will be highlighted in the graph. For rings, the root-mean-square deviation (RMSD) between the ring in question and similar rings identified by Mogul is calculated over all ring torsion angles. If the average RMSD is greater than 60 degrees and the minimal RMSD between the ring in question and any Mogul-identified rings is also greater than 60 degrees, then that ring is considered an outlier. The outliers are highlighted in purple. The color gray indicates Mogul did not find sufficient equivalents in the CSD to analyse the geometry.

For Manuscript Review

Ligand EVP D 102

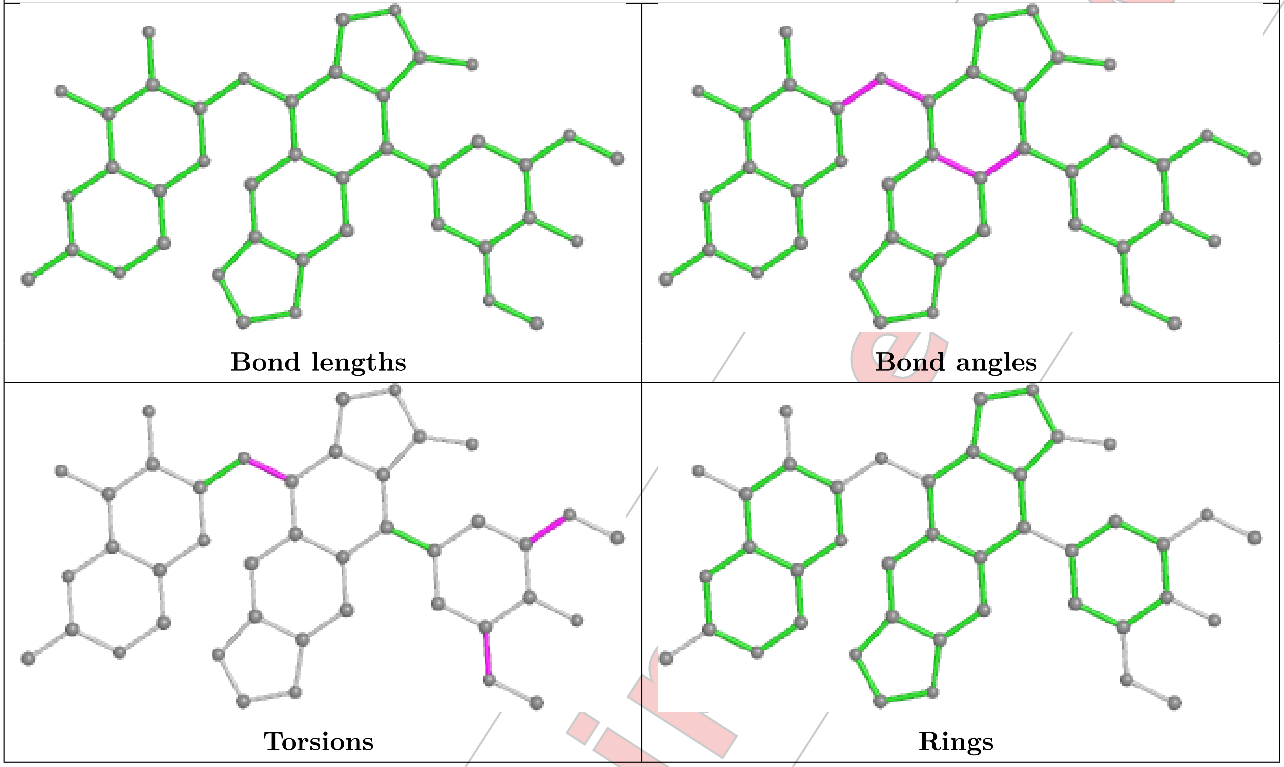

Ligand EVP D 101

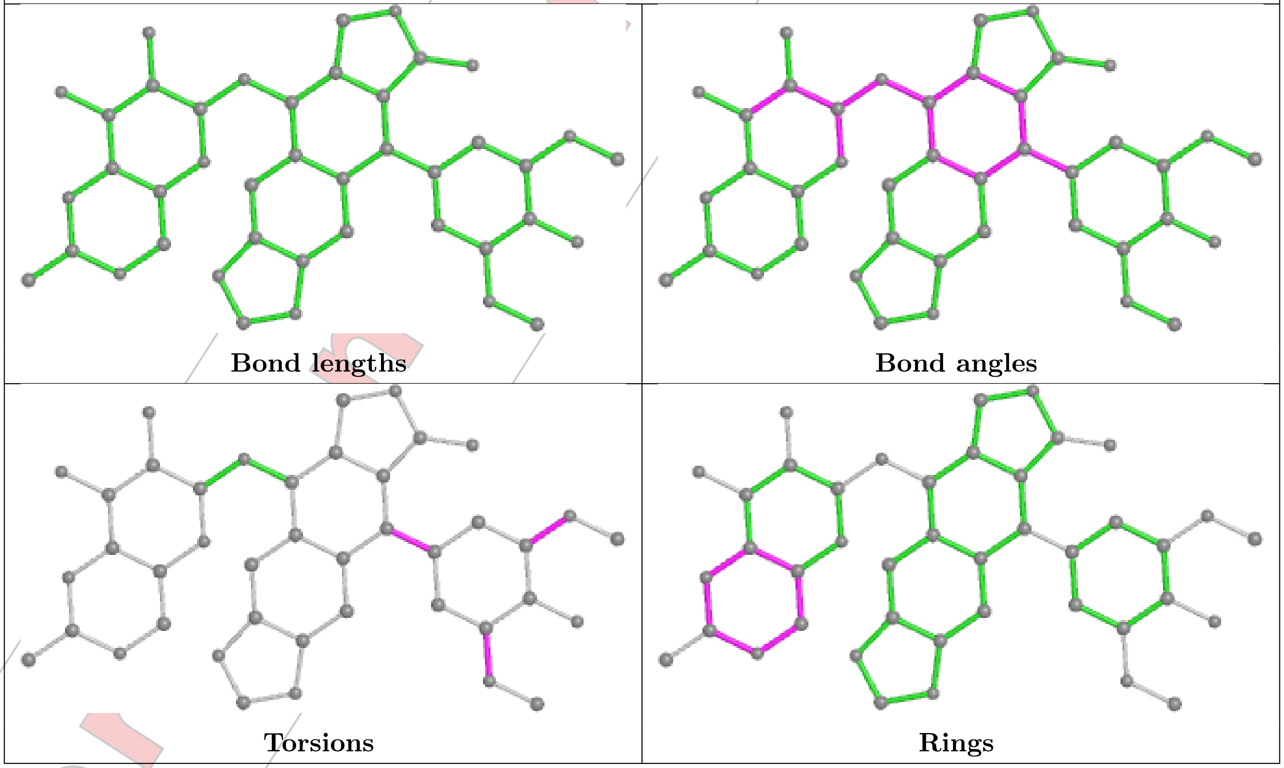

5.7 Other polymers (i)

There are no such residues in this entry.

5.8 Polymer linkage issues (i)

There are no chain breaks in this entry.

For Manuscript Review

6 Map visualisation ⓘ

This section contains visualisations of the EMDB entry EMD-36117. These allow visual inspection of the internal detail of the map and identification of artifacts.

Images derived from a raw map, generated by summing the deposited half-maps, are presented below the corresponding image components of the primary map to allow further visual inspection and comparison with those of the primary map.

6.1 Orthogonal projections ⓘ

6.1.1 Primary map

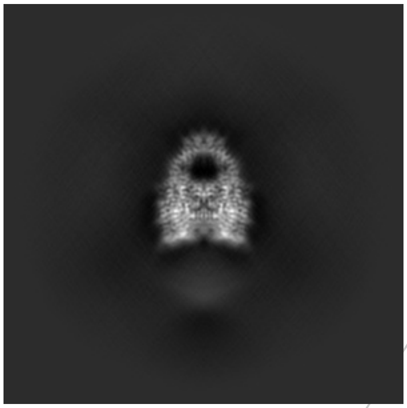

X

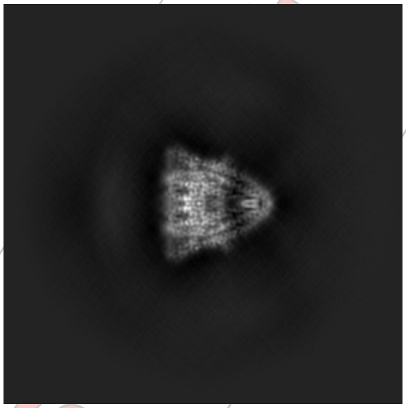

Y

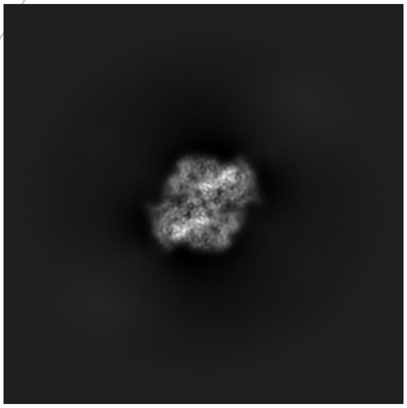

Z

6.1.2 Raw map

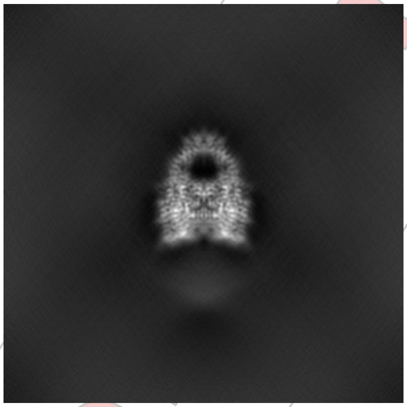

X

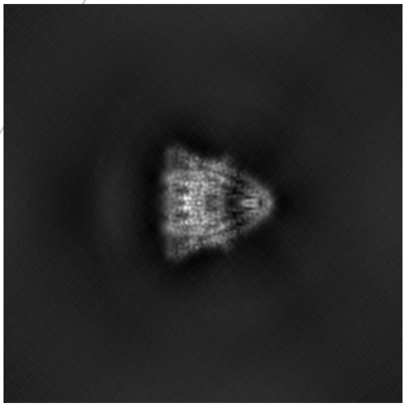

Y

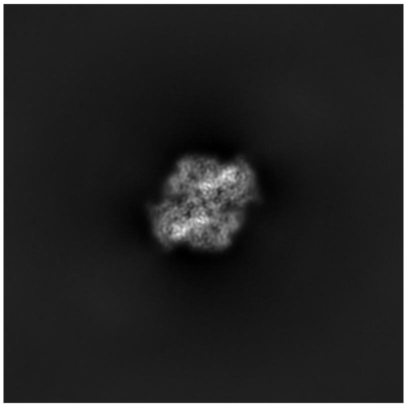

Z

The images above show the map projected in three orthogonal directions.

For Manuscript Review

6.2 Central slices (i)

6.2.1 Primary map

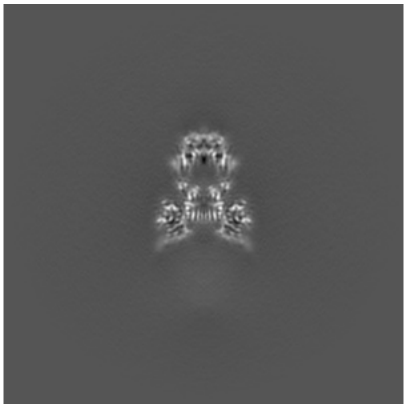
X Index: 216

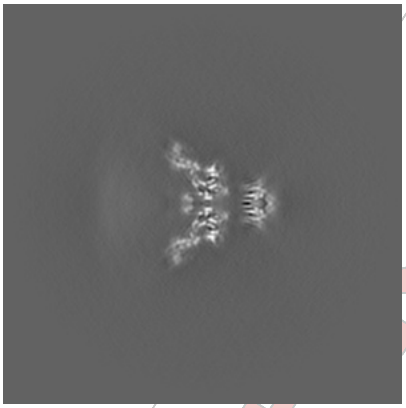
Y Index: 216

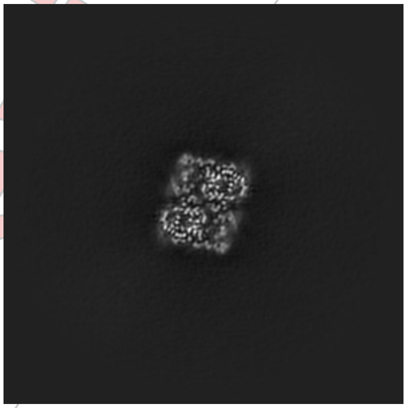
Z Index: 216

6.2.2 Raw map

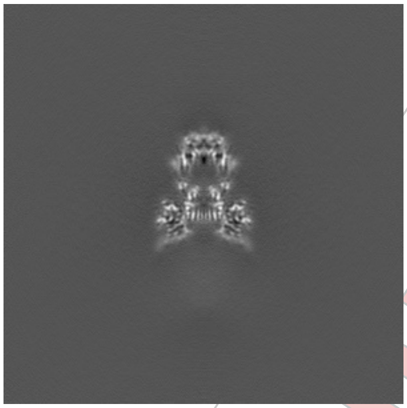
X Index: 216

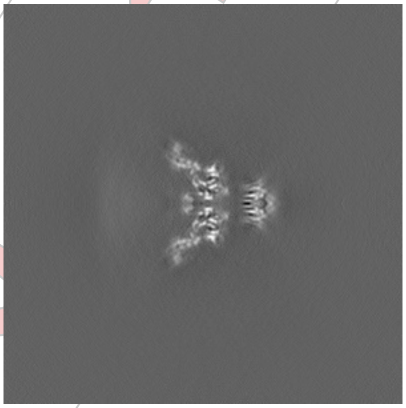
Y Index: 216

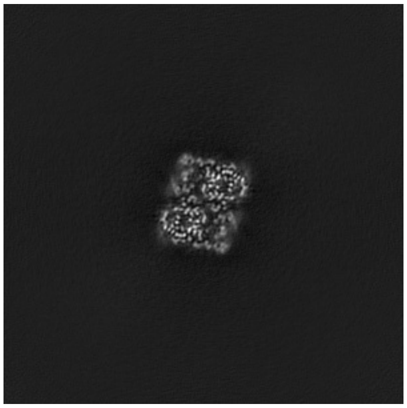
Z Index: 216

The images above show central slices of the map in three orthogonal directions.

For Manuscript Review

6.3 Largest variance slices (i)

6.3.1 Primary map

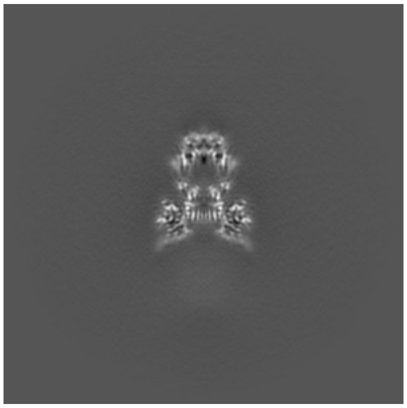

X Index: 216

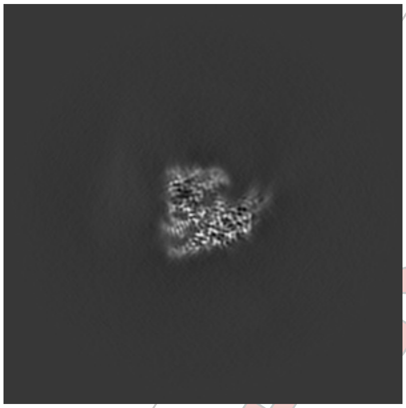

Y Index: 191

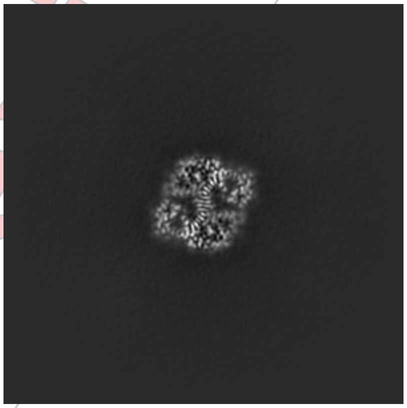

Z Index: 202

6.3.2 Raw map

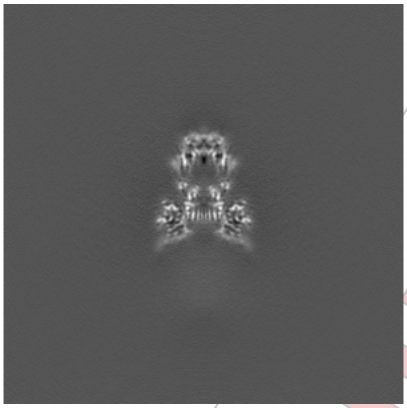

X Index: 216

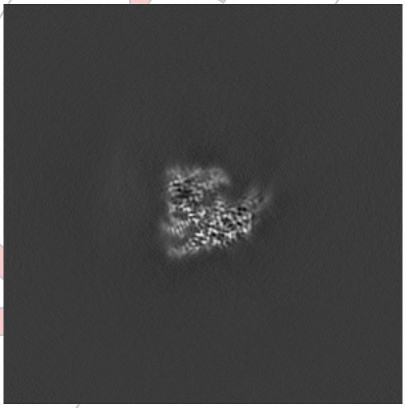

Y Index: 191

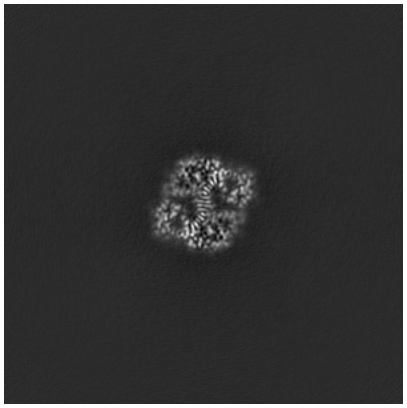

Z Index: 202

The images above show the largest variance slices of the map in three orthogonal directions.

For Manuscript Review

6.4 Orthogonal standard-deviation projections (False-color) (i)

6.4.1 Primary map

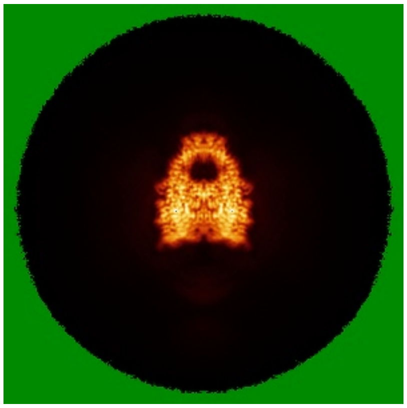
X

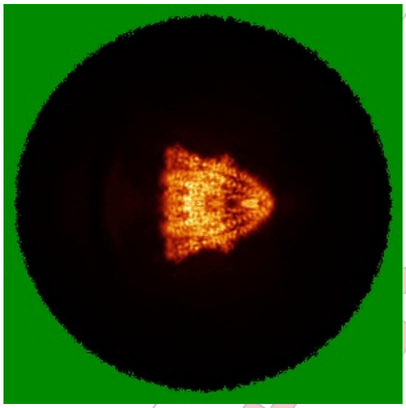
Y

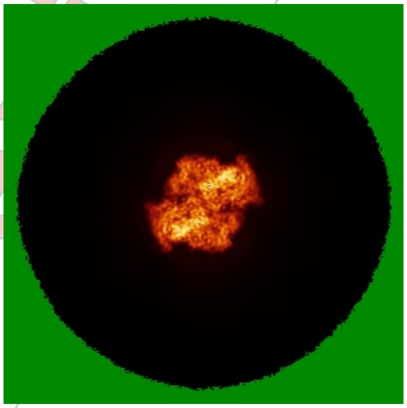
Z

6.4.2 Raw map

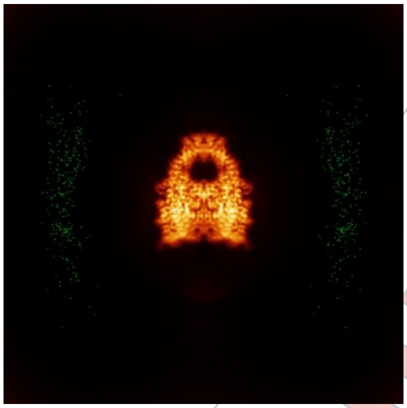
X

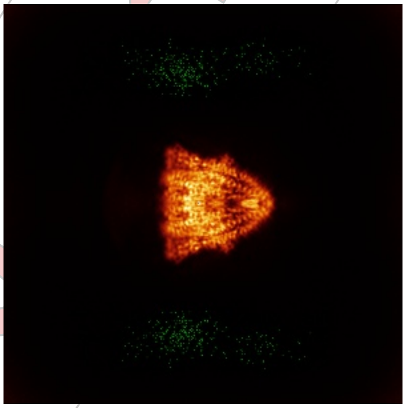
Y

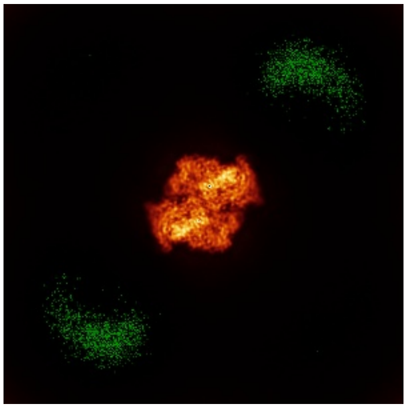
Z

The images above show the map standard deviation projections with false color in three orthogonal directions. Minimum values are shown in green, max in blue, and dark to light orange shades represent small to large values respectively.

For Manuscript Review

6.5 Orthogonal surface views (i)

6.5.1 Primary map

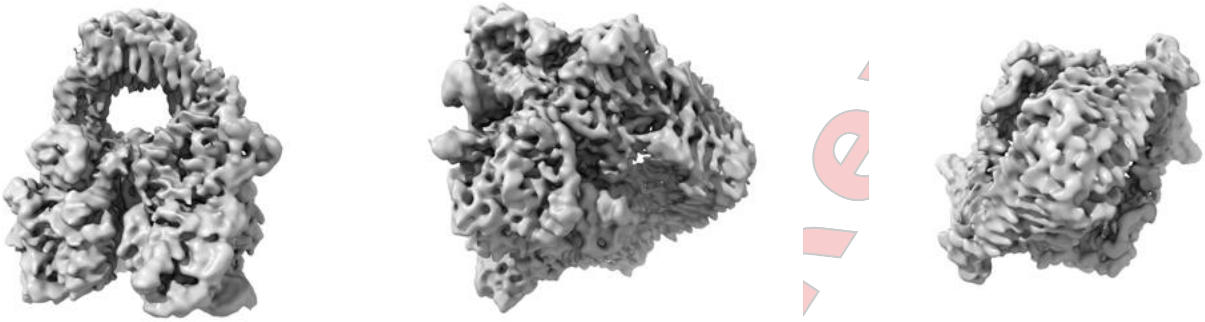

X Y Z

The images above show the 3D surface view of the map at the recommended contour level 0.195. These images, in conjunction with the slice images, may facilitate assessment of whether an appropriate contour level has been provided.

6.5.2 Raw map

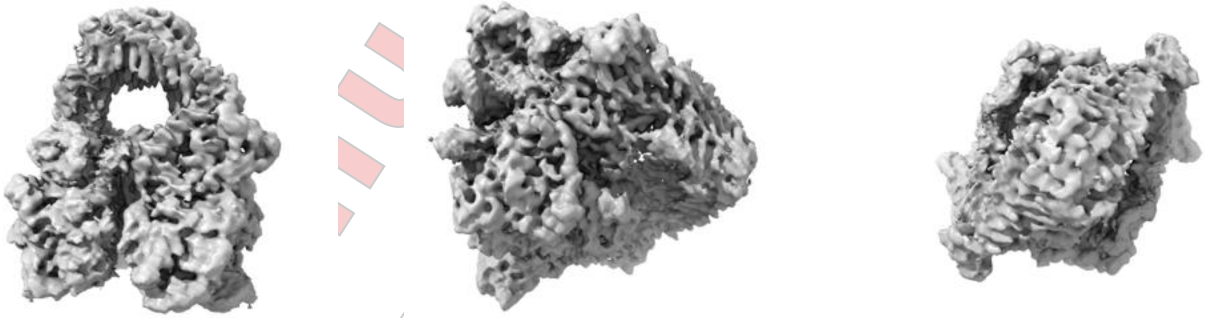

X Y Z

These images show the 3D surface of the raw map. The raw map's contour level was selected so that its surface encloses the same volume as the primary map does at its recommended contour level.

6.6 Mask visualisation (i)

This section was not generated. No masks/segmentation were deposited.

For Manuscript Review

7 Map analysis (i)

This section contains the results of statistical analysis of the map.

7.1 Map-value distribution (i)

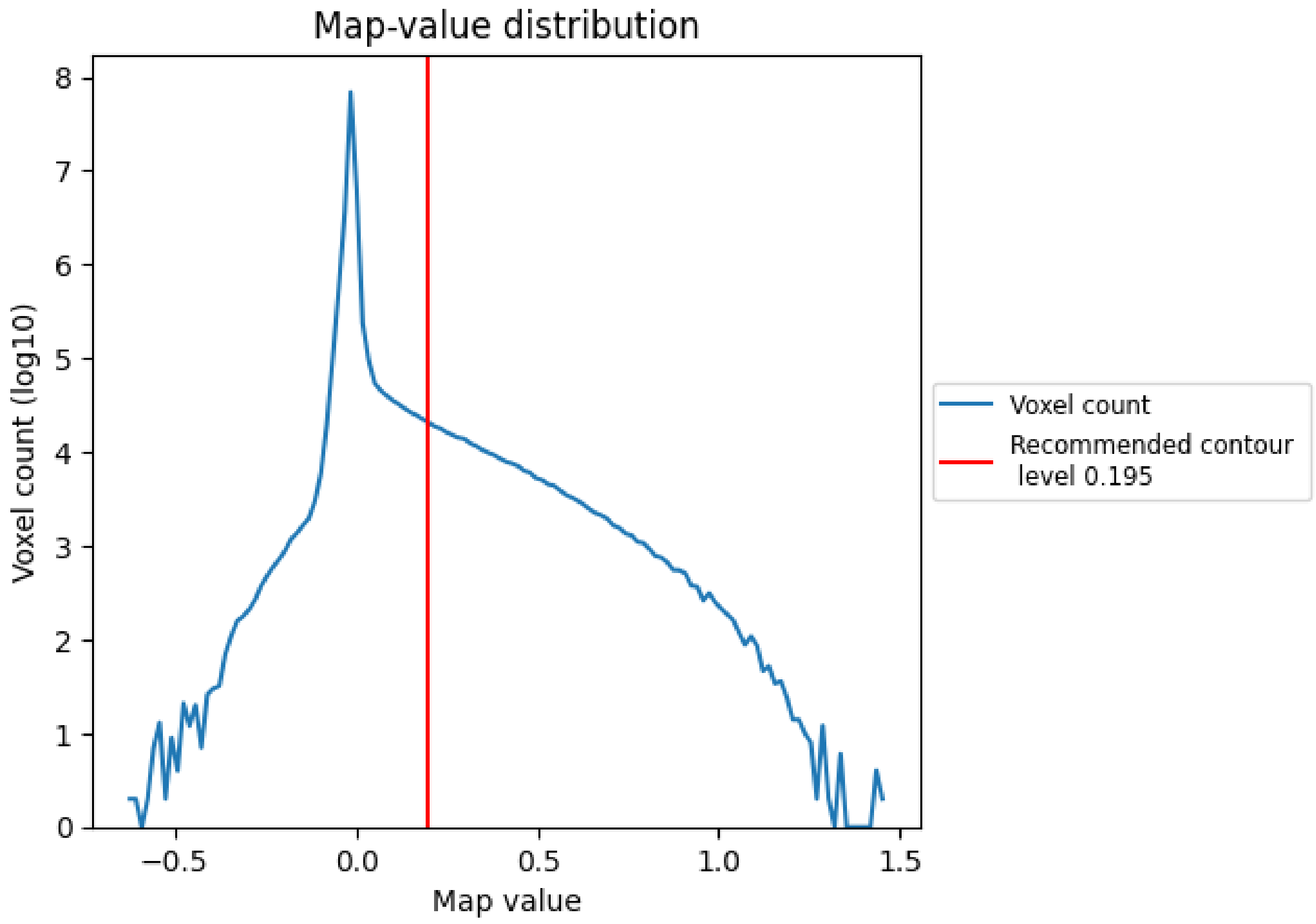

The map-value distribution is plotted in 128 intervals along the x-axis. The y-axis is logarithmic. A spike in this graph at zero usually indicates that the volume has been masked.

For Manuscript Review

7.2 Volume estimate (i)

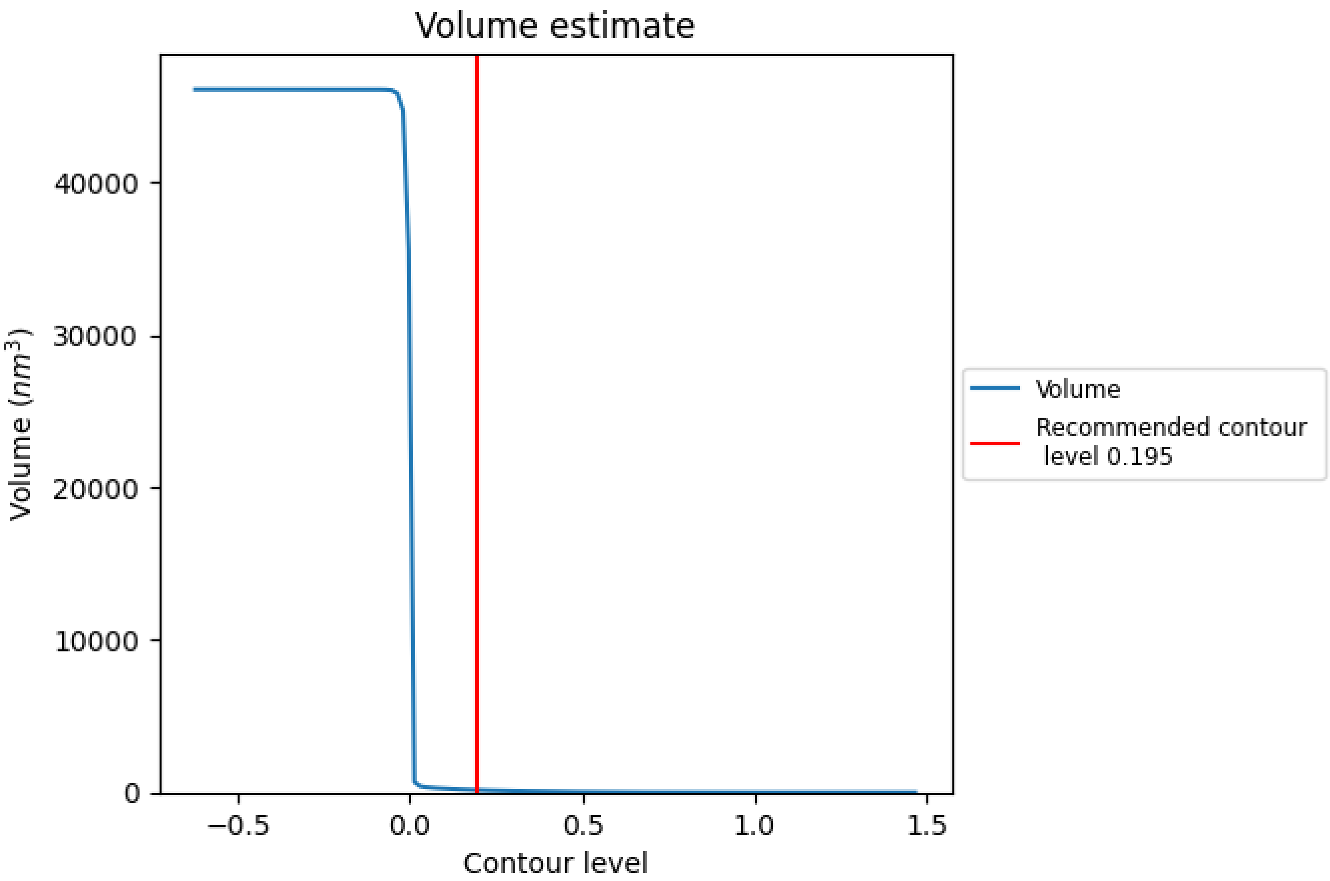

The volume at the recommended contour level is 160 nm^3; this corresponds to an approximate mass of 145 kDa.

The volume estimate graph shows how the enclosed volume varies with the contour level. The recommended contour level is shown as a vertical line and the intersection between the line and the curve gives the volume of the enclosed surface at the given level.

For Manuscript Review

7.3 Rotationally averaged power spectrum (i)

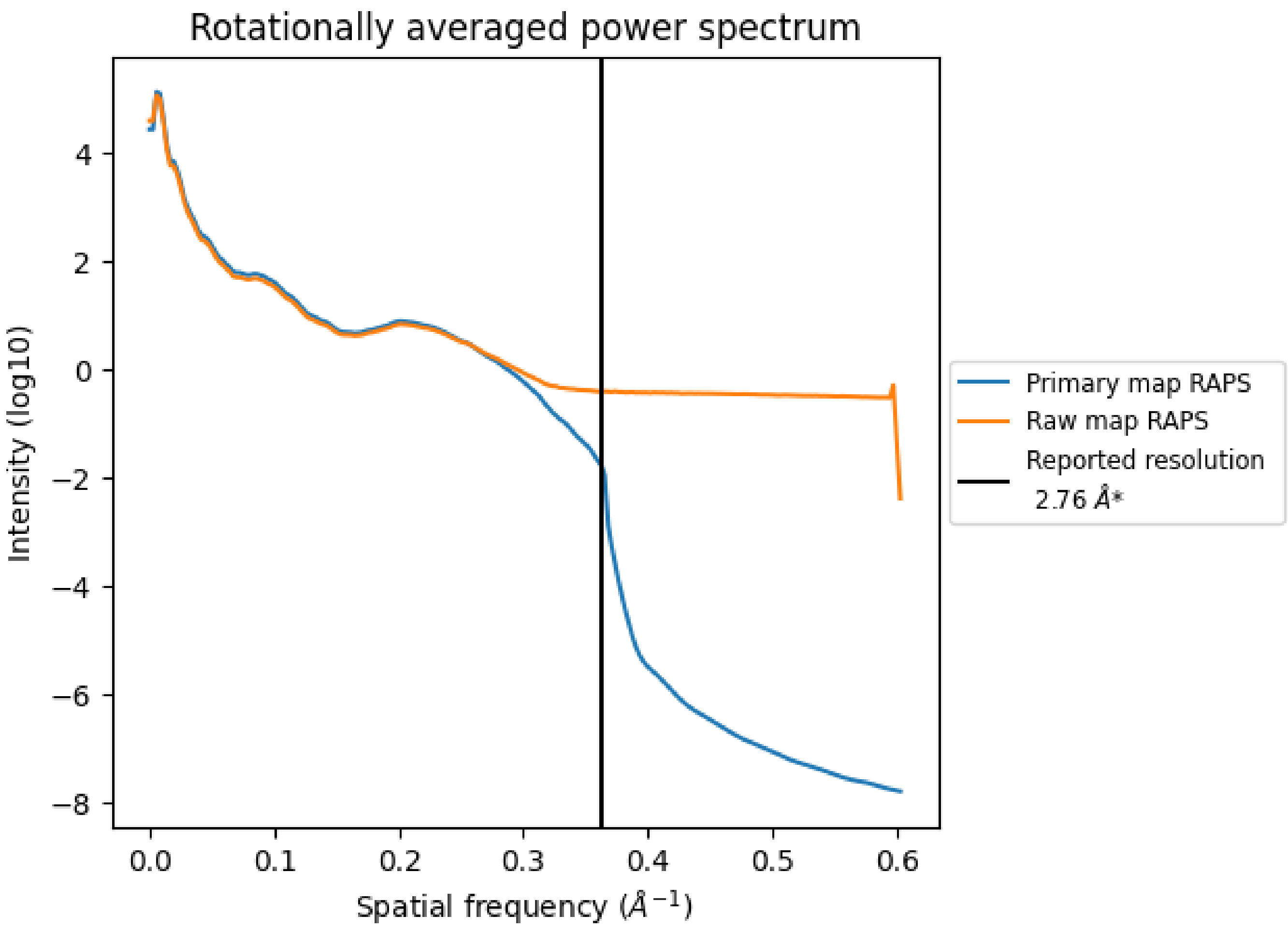

*Reported resolution corresponds to spatial frequency of 0.362 Å^{-1}

For Manuscript Review

8 Fourier-Shell correlation (i)

Fourier-Shell Correlation (FSC) is the most commonly used method to estimate the resolution of single-particle and subtomogram-averaged maps. The shape of the curve depends on the imposed symmetry, mask and whether or not the two 3D reconstructions used were processed from a common reference. The reported resolution is shown as a black line. A curve is displayed for the half-bit criterion in addition to lines showing the 0.143 gold standard cut-off and 0.5 cut-off.

8.1 FSC (i)

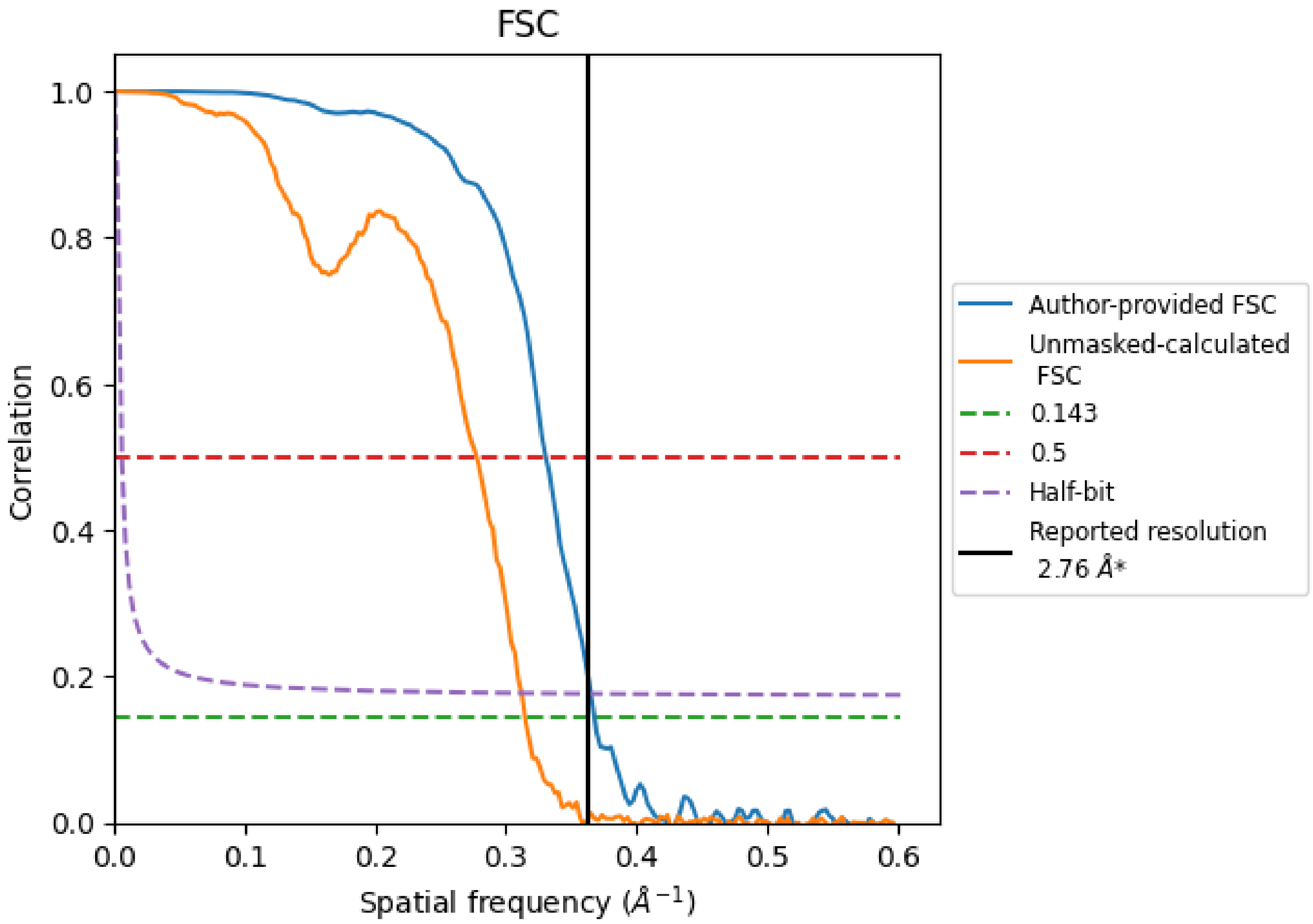

*Reported resolution corresponds to spatial frequency of 0.362 Å^{-1}

For Manuscript Review

8.2 Resolution estimates (i)

Resolution estimate (Å)	Estimation criterion (FSC cut-off)		
	0.143	0.5	Half-bit
Reported by author	2.76	-	-
Author-provided FSC curve	2.72	3.02	2.74
Unmasked-calculated*	3.18	3.60	3.21

*Resolution estimate based on FSC curve calculated by comparison of deposited half-maps. The value from deposited half-maps intersecting FSC 0.143 CUT-OFF 3.18 differs from the reported value 2.76 by more than 10 %

For Manuscript Review

9 Map-model fit (i)

This section contains information regarding the fit between EMDB map EMD-36117 and PDB model 8J9W. Per-residue inclusion information can be found in section 3 on page 5.

9.1 Map-model overlay (i)

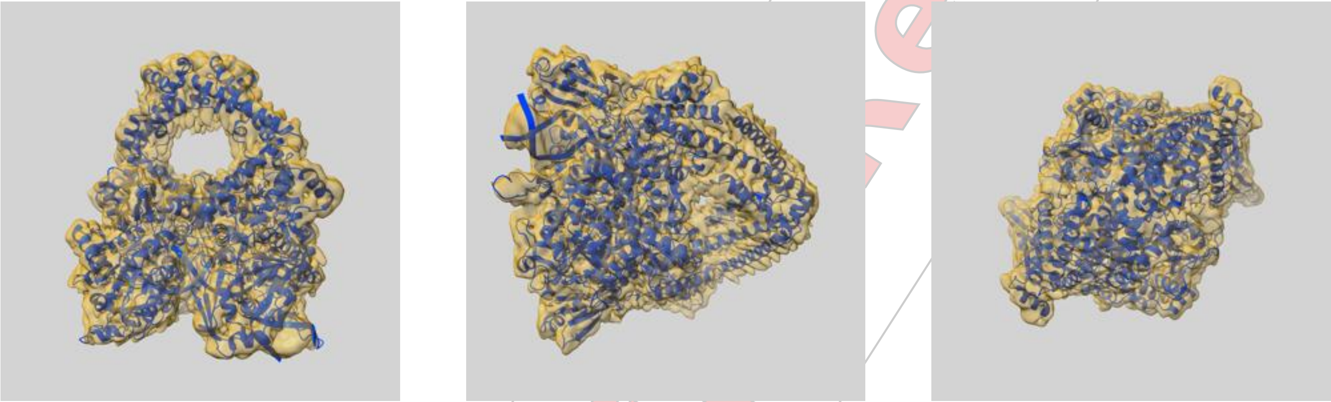

X Y Z

The images above show the 3D surface view of the map at the recommended contour level 0.195 at 50% transparency in yellow overlaid with a ribbon representation of the model coloured in blue. These images allow for the visual assessment of the quality of fit between the atomic model and the map.

For Manuscript Review

9.2 Q-score mapped to coordinate model (i)

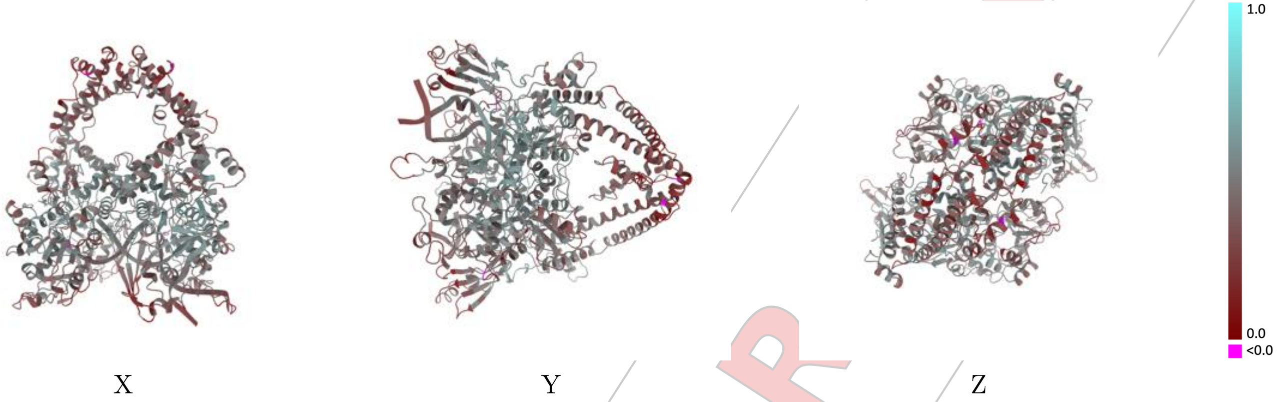

The images above show the model with each residue coloured according its Q-score. This shows their resolvability in the map with higher Q-score values reflecting better resolvability. Please note: Q-score is calculating the resolvability of atoms, and thus high values are only expected at resolutions at which atoms can be resolved. Low Q-score values may therefore be expected for many entries.

9.3 Atom inclusion mapped to coordinate model (i)

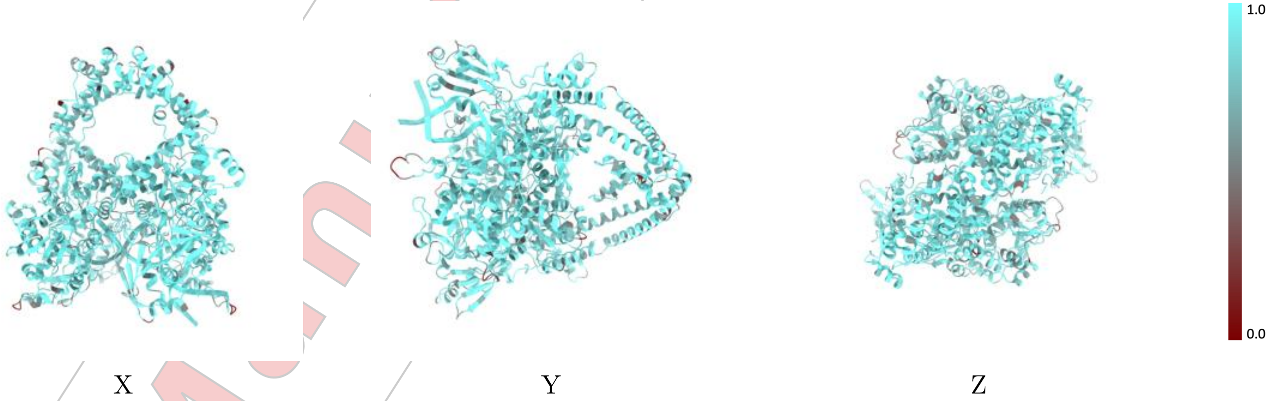

The images above show the model with each residue coloured according to its atom inclusion. This shows to what extent they are inside the map at the recommended contour level (0.195).

For Manuscript Review

9.4 Atom inclusion (i)

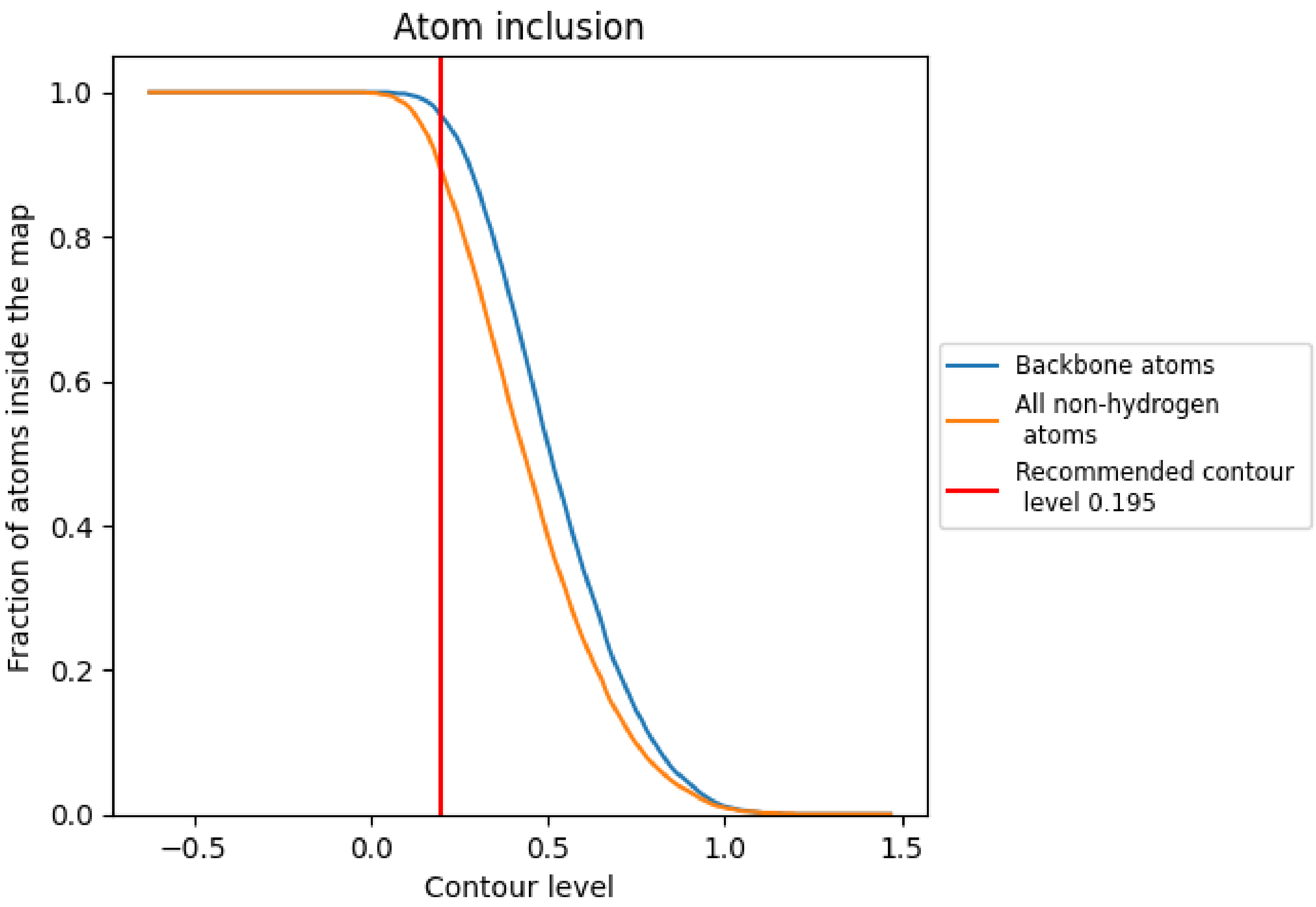

At the recommended contour level, 97% of all backbone atoms, 90% of all non-hydrogen atoms, are inside the map.

For Manuscript Review

9.5 Map-model fit summary (i)

The table lists the average atom inclusion at the recommended contour level (0.195) and Q-score for the entire model and for each chain.

Chain	Atom inclusion	Q-score
All	0.8970	0.4270
A	0.8930	0.4230
B	0.8940	0.4250
C	0.9510	0.4510
D	0.9280	0.4690
E	0.9250	0.4530
F	0.9250	0.4640

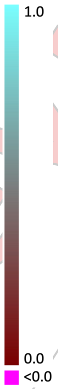

For Manuscript Review
