# Supplementary material for: A unified view on enzyme catalysis by cryo-EM study of a DNA topoisomerase
Source: Commun Chem. 2024 Feb 28;7:45. doi: 10.1038/s42004-024-01129-y (PMC10901890; doi:10.1038/s42004-024-01129-y)
Supplement: Supplementary file 1 — Peer Review File [file 42004_2024_1129_MOESM1_ESM.pdf]

nature portfolio

Peer Review File

A unified view on enzyme catalysis by cryo-EM study of a DNA topoisomerase

Open Access This file is licensed under a Creative Commons Attribution 4.0 International License, which permits use, sharing, adaptation, distribution and reproduction in any medium or format, as long as you give appropriate credit to the original author(s) and the source, provide a link to the Creative Commons license, and indicate if changes were made. In the cases where the authors are anonymous, such as is the case for the reports of anonymous peer reviewers, author attribution should be to 'Anonymous Referee' followed by a clear attribution to the source work. The images or other third party material in this file are included in the article's Creative Commons license, unless indicated otherwise in a credit line to the material. If material is not included in the article's Creative Commons license and your intended use is not permitted by statutory regulation or exceeds the permitted use, you will need to obtain permission directly from the copyright holder. To view a copy of this license, visit http://creativecommons.org/licenses/by/4.0/.

This manuscript has been previously reviewed at another Nature Portfolio journal. This document only contains reviewer comments and rebuttal letters for versions considered at Communications Chemistry.

REVIEWERS' COMMENTS:

Reviewer #1 (Remarks to the Author):

I am satisfied with the authors' response to my previous comments on the manuscript. The revisions have improved the presentation of the results, hypotheses and provided further support of the conclusions. The observation and characterization of multiple conformational states for AsfvTop2 by cryo-EM at the same time is a significant finding and provide new insights into the catalytic mechanism. The proposed catalytic selection, induced fit mechanism and regulation by redox are interesting hypotheses that should be of interest to many scientists.

Reviewer #2 (Remarks to the Author):

The authors have addressed all reviewers' concerns. The data presented in the manuscript support the conclusion and provide useful information for anti-ASFV drug development. I only have a suggestion listed below.

Three globally distinct conformational states from one single dataset of apo-ASFVTop2 have been reported in previous study (Zhao et al., mBio 2023, e0122823). The authors should explicitly cite this study.
